# Metabolomics, Molecular Networking and Phytochemical Investigation of *Psiadia dentata* (Cass.) DC., Endemic to Reunion Island: Discovery of Novel Bioactive Molecules

**DOI:** 10.3390/molecules31060973

**Published:** 2026-03-13

**Authors:** Lantomalala Elsa Razafindrabenja, Keshika Mahadeo, Gaëtan Herbette, Lúcia Mamede, Michel Frederich, Carole Di Giorgio, Béatrice Baghdikian, Patricia Clerc, Hippolyte Kodja, Isabelle Grondin, Anne Gauvin-Bialecki

**Affiliations:** 1 Laboratoire de Chimie et de Biotechnologie des Produits Naturels (ChemBioPro), Université de La Réunion, 15 Avenue René Cassin, CS 92 003, 97744 Saint-Denis Cedex 9, La Réunion, France; lantomalala.razafindrabenja@univ-reunion.fr (L.E.R.); keshika.mahadeo@univ-reunion.fr (K.M.); isabelle.grondin@univ-reunion.fr (I.G.); 2 SPO, University of Montpellier, INRAE, Institut Agro, 34060 Montpellier, France; 3 Aix-Marseille Université, CNRS, Centrale Méditerranée, FSCM, Spectropole, Campus de St Jérôme-Service 511, 13397 Marseille, France; gaetan.herbette@univ-amu.fr; 4 Université Paris-Saclay, CNRS, Institut de Chimie des Substances Naturelles, UPR 2301, 91198 Gif-sur-Yvette, France; 5 Pharmacognosy Laboratory, Department of Pharmacy, Centre Interfacultaire de Recherche sur le Médicament (CIRM), University of Liège, Campus du Sart-Tilman, Quartier Hôpital, Avenue Hippocrate, 15, B36, 4000 Liège, Belgium; lucia.c.mamede@outlook.pt (L.M.); m.frederich@uliege.be (M.F.); 6 IMBE, Aix Marseille Université, Avignon Université, CNRS 7263, IRD 237, 27 Bd Jean Moulin, Service of Environmental Mutagenesis, Faculty of Pharmacy, 13385 Marseille, France; carole.di-giorgio@univ-amu.fr; 7 IMBE, Aix Marseille Université, Avignon Université, CNRS 7263, IRD 237, 27 Bd Jean Moulin, Service of Pharmacognosy, Faculty of Pharmacy, 13385 Marseille, France; beatrice.baghdikian@univ-amu.fr; 8 Qualisud, University Montpellier, CIRAD, Institut Agro, Avignon Université, Université de La Réunion, 34093 Montpellier, France; hippolyte.kodja@univ-reunion.fr

**Keywords:** *Psiadia dentata*, metabolomics, PLS-DA, molecular networking, diterpenes, coumarin, antiplasmodial, anti-inflammatory, cytotoxic activities

## Abstract

The genus *Psiadia* (Asteraceae), widely distributed in Madagascar and the Mascarene Islands (Mauritius, La Réunion, Rodrigues), is traditionally used to treat bronchitis, asthma, colds, abdominal pain, and other inflammatory disorders. However, few studies have scientifically validated these traditional medicinal uses. To assess *P. dentata* as a valuable source of bioactive natural products, a combined ^1^H NMR-based metabolomic, molecular networking, and phytochemical study was conducted. Multivariate analysis (PLS-DA) of crude extracts from *Psiadia* species collected on Reunion Island enabled rapid discrimination of active extracts from *P. dentata* and revealed two methoxylated flavonoids and one coumarin as metabolites correlated with its antiplasmodial and anti-inflammatory activities. Additionally, UHPLC-DAD-ESI-QTOF-MS/MS molecular networking approach enabled detailed chemical profiling of this species, allowing the annotation of 25 compounds (**1**–**25**) in this species. Subsequent phytochemical investigation of *P. dentata* leaves led to the isolation and identification of 25 metabolites, including nine new diterpenes (**26**–**34**), one new coumarin (**35**), and 15 known compounds (**1**–**8**, **11**, **18**, **19** and **36**–**39**) from the diterpenoid, flavonoid, and coumarin families. The structures of the new compounds were elucidated using spectroscopic methods, including extensive 1D and 2D NMR and HRESIMS analyses. Biological evaluation of the isolated compounds showed that compounds **1**, **7**, **26** and **27** showed antiplasmodial activity against *Plasmodium falciparum* (3D7 strain, IC_50_ = 7.25–13.46 μM). Compounds **7**, **26**, **27**, **31** and **32** inhibited nitric oxide production (IC_50_ = 0.87–27.71 μM), indicating potential anti-inflammatory effects. Only compound **1** displayed moderate cytotoxicity against HepG2 and HT29 cancer cell lines (IC_50_ = 25.67 and 18.35 μM, respectively).

## 1. Introduction

The importance of plant-specialized metabolites in medicine has encouraged many researchers to investigate the phytochemistry of diverse plants. Indeed, medicines derived from natural sources constitute a significant proportion of our therapeutic drugs. Over the past four decades, more than 400 naturally occurring products and their structural derivatives have been approved as therapeutic agents [1]. Their structural diversity and biological relevance have led to major advances in the treatment of cancer, infectious diseases, and inflammatory disorders.

Advances in phytochemistry and analytical chemistry have significantly enhanced our ability to explore plant composition in depth and to identify bioactive constituents through high-throughput, integrative approaches, such as metabolomics or molecular networking. These advanced strategies allow rapid profiling of complex mixtures and offer an improved expedited route for drug discovery.

Metabolomics is the comprehensive, large-scale study of primary and specialized metabolites in an organism at a given time. The development of analytical tools for metabolomics, such as proton nuclear magnetic resonance (^1^H NMR) spectroscopy and mass spectrometry (MS), has led to rapid progress in this field. ^1^H NMR analysis is currently used for the simultaneous detection and quantification of multiple metabolites in a single, non-destructive measurement with minimal sample preparation [2]. This approach has been widely applied in clinical research to elucidate disease mechanisms or identify biomarkers for disease diagnosis [3]. ^1^H NMR-based metabolomic fingerprinting, combined with statistical analysis methods, has received considerable attention in the quality control of medicinal plants used in herbal preparations, particularly for detecting adulteration and identifying adulterants [4,5,6,7]. Recently, metabolomics has emerged as a rapid, alternative screening method to correlate chemical and biological data of natural products. Several studies have demonstrated the ability of NMR spectroscopy, when coupled with multivariate analysis, to predict the bioactivity of plant extracts. For instance, Heyman et al. showed that metabolomics could be used to discriminate bioactive fractions and identify bioactive compounds in crude extracts without requiring bioassay-guided fractionation and purification steps [8,9,10].

Another powerful and emerging approach to the dereplication of complex natural products is liquid chromatography-tandem mass spectrometry (LC-MS/MS)-based molecular networking. This approach organizes and visualizes MS/MS data through a spectral similarity map, revealing clusters of compounds that share similar fragmentation patterns [11]. Using this tool, comprehensive metabolic profiling can be achieved through a single analysis of the plant extract. The introduction of Global Natural Product Social molecular networking (GNPS) web platform (http://gnps.ucsd.edu; accessed on 22 May 2020) has enabled automatic spectral mining [12,13].

**Figure 1:**
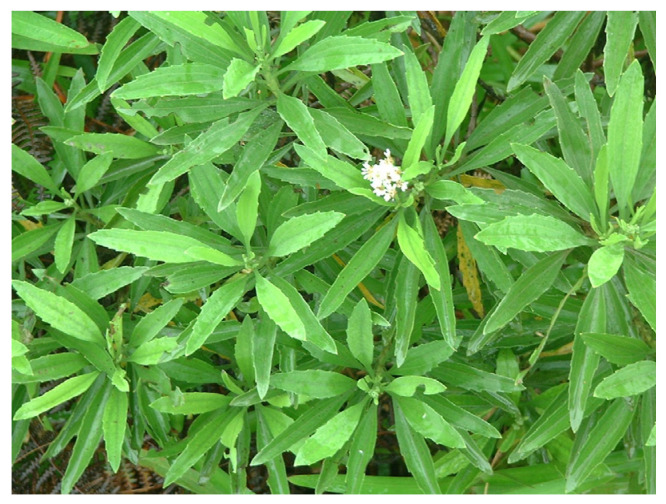
Exterior view of *P. dentata* (Cass.) DC. (Photo by the author, 2015).

In the present work, the contributions of these approaches to identify molecular families and putative bioactive candidate molecules in natural product research are explored. As part of our ongoing efforts to discover bioactive natural products within the genus *Psiadia*, and to apply this integrated metabolomic and phytochemical strategy, *Psiadia dentata* (Cass.) DC. (Figure 1), an endemic species of Reunion Island with recognized ethnobotanical and pharmacological relevance, was selected for this study.

Species of the genus *Psiadia*, belonging to the (Asteraceae) family, are native to both Madagascar and the Mascarene Islands (Reunion, Mauritius and Rodrigues). This genus comprises approximately 28 species in Madagascar and 26 species in the Mascarene Islands [14]. The leaves of *Psiadia* plants have long been used in ethnomedicine to treat a variety of ailments such as abdominal pains, fever, bronchitis, and asthma [15]. *Psiadia dentata* (Cass.) DC. commonly known as *Ti-mangue*, *Bois collant*, *Bois de marron*, *Bois de reinette blanc*, or *Herbe trois jours* [14], is traditionally used in the form of cataplasm to treat abscesses and skin infections caused by fungi or insect bites [16,17]. In previous studies, *P. dentata* has demonstrated a range of interesting biological activities, including antiviral [16,17,18,19,20], anti-inflammatory [21], antiplasmodial [21,22] and cytotoxic effects [21].

Despite this therapeutic promise and the preliminary phytochemical and bioactivity studies reported for *P. dentata*, the chemical diversity and pharmacological potential of this species remain largely underexplored. Only a limited number of metabolites have been identified to date, including five flavonoids [16,23] and one coumarin [17,23]. Furthermore, few studies have scientifically validated its traditional medicinal uses, leaving most of its metabolome uncharacterized and structure–activity relationships entirely unexplored [15].

In this context, the present work was undertaken to address this gap through an integrative investigation of the leaf metabolites of *P. dentata* using a combination of NMR metabolomics, molecular networking (MN), and phytochemical analysis. To the best of our knowledge, no comprehensive study combining these complementary approaches has yet been conducted on this species. The integration of NMR metabolomics and molecular networking provides a more holistic view of the metabolome. The MN approach, implemented using LC–MS/MS, facilitates the identification of structurally related molecules and the visualization of their relationships. In parallel, NMR-based metabolomics provides complementary relative quantitative information on metabolite abundance within a sample and reveals signatures linking metabolite profiles to biological activity. Finally, the phytochemical investigation, involving fractionation, isolation, and structure elucidation, confirmed the results obtained from the metabolomic study and the molecular networking analysis.

As a first step, anti-inflammatory and antiplasmodial activities of 11 species endemics to Reunion Island were evaluated. In the second step, a UHPLC-DAD-ESI-QTOF-MS/MS molecular networking approach was applied to *P. dentata* in order to explore its chemical diversity. The relationships between the metabolites and the biological activity of each species extracts were also evaluated separately within the NMR datasets, coupled with multivariate analysis. Following these analyses, eight new diterpenes, one new coumarin, and 15 known compounds were isolated and identified from the EtOAc (ethyl acetate) extract of *P. dentata* leaves. These compounds were tested for their *in vitro* antiplasmodial, anti-inflammatory and cytotoxicity properties. All the obtained results are discussed below.

## 2. Results and Discussion

### 2.1. Biological Activities of Psiadia Species

**Figure 2:**
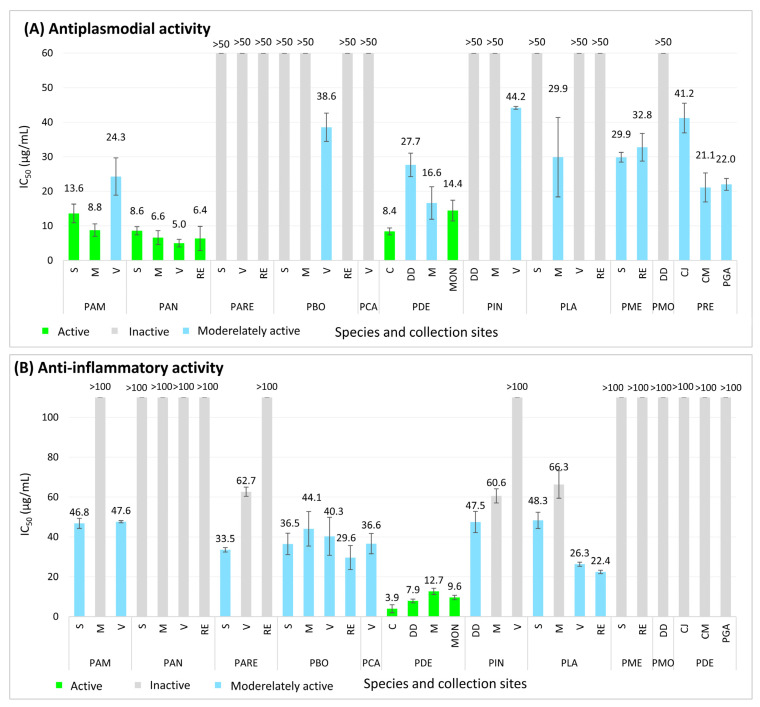
(**A**) *In vitro* antiplasmodial activity (3D7 strain) of *Psiadia* species crude extracts (IC_50_ in μg/mL). Positive control: artemisinin (IC_50_ = 0.004 ± 0.001 μg/mL). Inactive extract (IC_50_ ≥ 50 μg/mL). (**B**) *In vitro* anti-inflammatory activity (HRP enzyme) of *Psiadia* species crude extracts (IC_50_ in μg/mL). Positive control: quercetin (IC_50_ = 10.8 ± 1.9 μg/mL). Inactive extract (IC_50_ ≥ 100 μg/mL). Plant codes: PAM (*P. amygdalina*), PAN (*P. anchusifolia*), PARE (*P. argentea*), PBO (*P. boivinii*), PCA (*P. callocephala*), PDE (*P. dentata*), PIN (*P. insignis*), PLA (*P. laurifolia*), PME (*P. melastomatoides*), PMO (*P. montana*), PRE (*P. retusa*). Location codes: S (Salazie), M (Maïdo), V (Volcan), RE (Roche-Ecrite), C (Colorado), DD (Dos d’Ane), MON (Montauban), CJ (Cap Jaune), CM (Cap Méchant), PGA (Piton Grande Anse). Active compound: <15 µg/mL, moderately active compound: 15–50 µg/mL, inactive compound (IC_50_ ≥ 50 μg/mL) [24,25].

As part of our ongoing search for bioactive natural products within the genus *Psiadia*, eleven endemic species from Reunion Island were selected. To assess the geographical variability of their chemical profiles, several collection sites were analyzed for each species during the summer season. All ethyl acetate crude extracts were tested *in vitro* for their antiplasmodial activity against the chloroquine-sensitive (3D7) strain of *Plasmodium falciparum* and for their anti-inflammatory properties using the horseradish peroxidase (HRP) enzyme assay (Figure 2).

Among the tested extracts, three species exhibited significant *in vitro* antiplasmodial activity (IC_50_ < 15 µg/mL, according to the classification criteria of [24,25]): *P. amygdalina* (PAM), *P. anchusifolia* (PAN) and *P. dentata* (PDE). The activity of *P. amygdalina* and *P. dentata* extracts varied depending on the collection site, indicating chemical profile. In contrast, the antiplasmodial activity of *P. anchusifolia* crude extracts was only slightly affected by geographical location. Although the IC_50_ values of all tested crude extracts were higher than that of the positive control, artemisinin (IC_50_ = 0.004 ± 0.001 µg/mL), these extracts nevertheless remain promising for the discovery of antiplasmodial compounds.

With regard to anti-inflammatory activity, *P. dentata* was the only species to display significant *in vitro* activity against the HRP enzyme (IC_50_ < 15 µg/mL, according to [24,25]), with minimal variation across collection sites. Moreover, the anti-inflammatory activity of *Psiadia dentata* crude extracts, while lower than that of the positive control quercetin (IC_50_ = 10.8 ± 1.9 µg/mL), suggests the presence of highly active compounds in these extracts.

Crude extracts are complex mixtures containing hundreds of molecules, some of which may exhibit high biological activity when isolated. Based on the biological activity results, *Psiadia dentata* was selected as the most promising species due to its potent anti-inflammatory and antiplasmodial properties. Consequently, to identify the compound families or specific molecules that are responsible for these activities, a metabolomic analysis was conducted on the plant extracts using ^1^H NMR spectroscopy.

### 2.2. Molecular Networking

**Figure 3:**
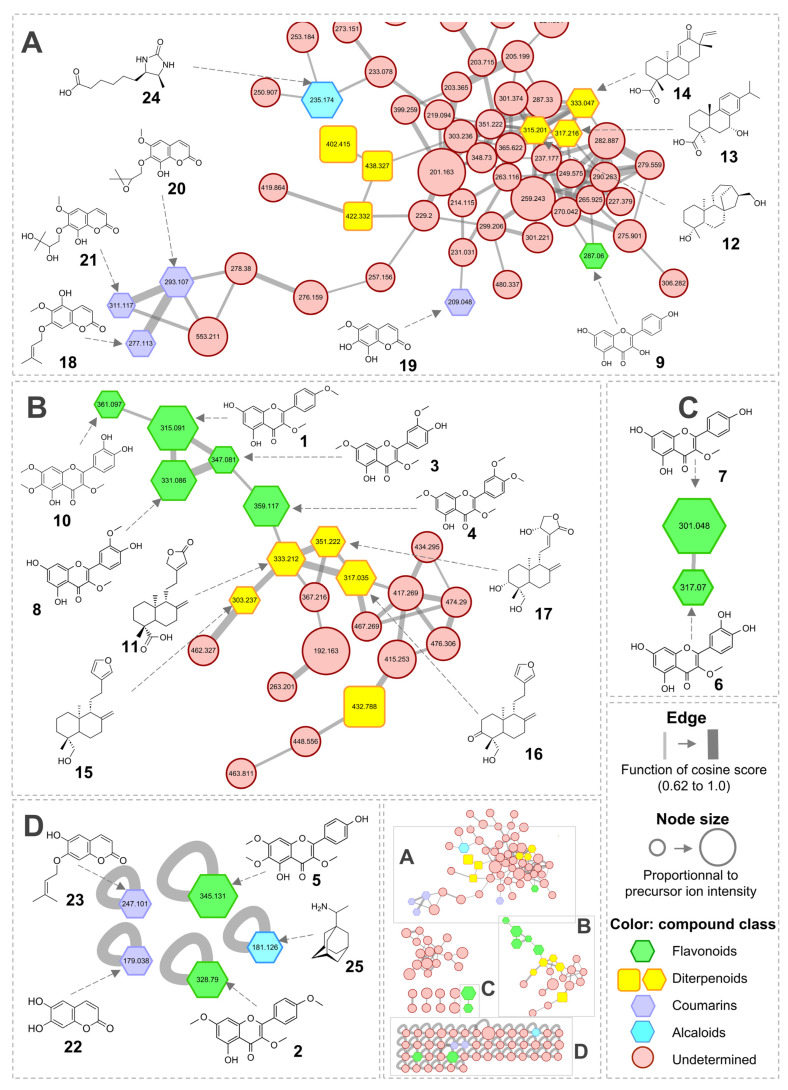
Annotation of the molecular network of isohexane and methanolic fractions derived from *P. dentata*.

To obtain the first molecular fingerprint of *P. dentata* and gain insights about the chemodiversity of the species, by highlighting distinct metabolite features, a MN approach was performed. Isohexane and methanolic fractions of the crude extract were analyzed by UHPLC-HRMS-ESI-QTOF in positive mode. The resulting data were subsequently processed on the GNPS platform, where tandem mass spectrometry (MS/MS) spectra were compared, organized and visualized through spectral similarity mapping, revealing clusters of structurally related metabolites [26]. The generated MS/MS molecular network is shown in Figure 3. It consists of 154 nodes, forming 8 clusters (connected nodes), and 48 self-loops (unlinked nodes), using a cosine score threshold of 0.62. Dereplication was performed on GNPS using a large, community-acquired spectral library. Additional in silico databases, such as MetFrag [27] and CFM-ID [28], were employed to support the compound annotation complemented by manual inspection of the MS/MS.

Annotation of molecular networks revealed a rich chemical diversity, with 25 compounds distributed among four major molecular families: flavonoids, diterpenoids, coumarins, and alkaloids (Table 1).

The first family, flavonoids (Figure 3A–D) included 10 nodes (green) corresponding to known flavonoids previously reported in several *Psiadia* species [15], such as ermanin (**1**), kaempferol 3,7,4′-trimethylether (**1**), patchypodol (**3**), retusin (**4**), penduletin (**5**), quercetin 3-methylether (**6**), isokaempferide (**7**), quercetin 3,3′-dimethylether (**8**), kaempferol (**9**) and chrysosplenol D (**10**). Most of these compounds have been previously isolated from the leaves of *P. trinervia* [29], *P. terebinthina* [30], and *P. dentata* [16,23].

The second family observed consists of diterpenoids (yellow nodes) (Figure 3A,B). A total of 7 diterpenes were revealed, including *ent*-labda-8(17),13-dien-15,16-olide-19-oic acid (**11**), annosquamosine C (**12**), 7-hydroxycallitrisic acid (**13**), methyl-*ent*-12-oxopimara-9(11),15-dien-19-oic acid (**14**), 6-deoxypsiadiol (**15**), 3-oxo-19-hydroxy-13-furyl-ent-labda-8(17)-ene (**16**), and andrographolide (**17**). All of these compounds share a labdane-type structure, with bicyclic carbon skeletons and various oxygenated functional groups. Several labdane- and kaurane-type diterpenes in *Psiadia* species such as psiadiol and 6-deoxypsiadiol (**15**) [31], as well as *ent*-16β,17-dihydroxykauran-20-oic acid, 2-oxotrachyloban-18,19-diol, and trachyloban-2β,6β,19-triol [32] have previously been reported. Their occurrence in the MN is therefore consistent with these previous findings. In addition to the 7 annotated diterpenes, the MN also revealed seven other diterpenes that could not be annotated. However, based on their connectivity within the network and their fragmentation patterns, these compounds are also likely to share a labdane-type skeleton.

Another class of molecular compounds in MN was annotated as coumarins (purple nodes) (Figure 3A,D). Six coumarins were suggested: isoobtusitin (**18**), and fraxetin (**19**), 7-(2′,3′-epoxy-3′-methylbutoxy)-8-hydroxy-6-methoxycoumarin (**20**), 7-(2′,3′-dihydroxy-3′-methylbutoxy)-8-hydroxy-6-methoxycoumarin (**21**), esculetin (**22**) and prenyletin (**23**). Isoobtusitin has also been previously reported from *P. dentata* [17], confirming its consistent occurrence, whereas esculetin and prenyletin have been documented in several Asteraceae species [33,34].

Finally, two nodes potentially corresponded to alkaloids (light blue nodes) identified as desthiobiotin (**24**) and rimantadine (**25**) (Figure 3A,D). To date, no alkaloid has been isolated from species of the genus *Psiadia*, although many Asteraceae, especially *Senecio* species, are known sources of pyrrolizidine alkaloids [35].

Nodes that could not be annotated suggest the presence of compounds with a certain structural originality.

### 2.3. ^1^H NMR Metabolomic Analysis

#### 2.3.1. PLS-DA of ^1^H NMR Data of *P. dentata* Crude Extracts and Correlation with the Antiplasmodial Activity

**Figure 4:**
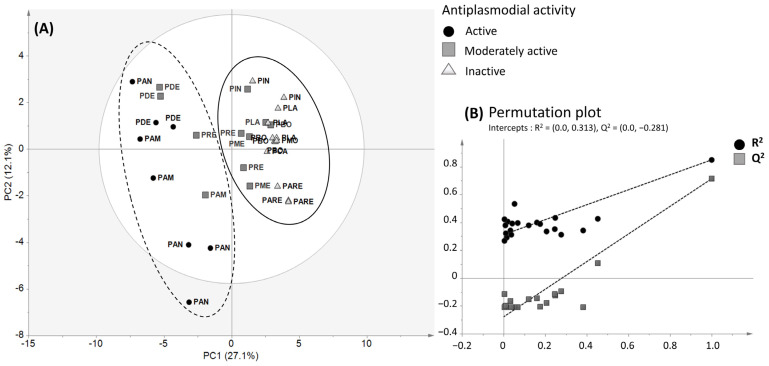
(**A**) PLS score plot derived from ^1^H NMR spectra (600 MHz) of crude extracts from *Psiadia* species. (**B**) Permutation plot. Plant codes: PAM (*P. amygdalina*), PAN (*P. anchusifolia*), PARE (*P. argentea*), PBO (*P. boivinii*), PCA (*P. callocephala*), PDE (*P. dentata*), PIN (*P. insignis*), PLA (*P. laurifolia*), PMO (*P. montana*), PME (*P. melastomatoides*), PRE (*P. retusa*).

The ^1^H NMR spectra of all crude extracts were analyzed using a partial least square (PLS) regression (Figure 4A) to evaluate the correlation between the antiplasmodial activity and the detected metabolites. The model was generated using a single quantitative Y variable corresponding to the IC_50_ values of each crude extract. The resulting PLS model exhibited the following statistical parameters: R^2^ (X) = 0.39, R^2^ (Y) = 0.84 and Q^2^ = 0.71. The R^2^, Q^2^ values, and the permutation test confirmed the model’s validity. From a general point of view, the samples tended to cluster into two distinct groups along the first principal component, which accounted for 27.1% of the total variation. On the PLS score plot, the distribution of samples within each cluster suggested distinct metabolic profiles. Interestingly, some moderately active extracts (IC_50_: 22 ≤ IC_50_ ≤ 44 µg/mL) were grouped with either active or inactive extracts, which may reflect a shared chemical composition and the presence of low levels of active compounds in the moderately active samples. In contrast, the moderately active extracts grouped with the active ones displayed lower IC_50_ (16 ≤ IC_50_ ≤ 22 μg/mL).

Therefore, to highlight molecules or compound families responsible for the antiplasmodial activity of *P. dentata* crude extracts, multivariate data analysis comparing the active crude extracts *versus* all inactive extracts was performed. Since the four *P. dentata* extracts (both active and moderately active extracts) are grouped, they seem to have a similar composition.

**Figure 5:**
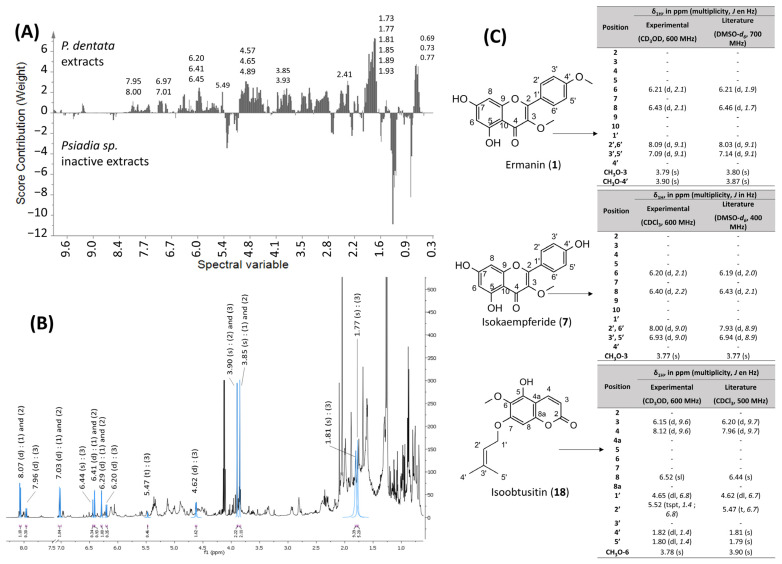
(**A**) Contribution plot of the PLS model comparing *P. dentata* active crude extracts with inactive extracts from other *Psiadia* species; (**B**) ^1^H NMR spectrum (CDCl_3_, 600 MHz) of an active crude extract of *P. dentata*, highlighting the assignments of ermanin (**1**), isokaempferide (**7**), and isoobtusitin (**18**); (**C**) molecular structures of ermanin (**1**), isokaempferide (**7**), and isoobtusitin (**18**), along with their experimental and literature ^1^H NMR data [17,40,41].

Therefore, a contribution plot was generated, with the comparison of all inactive extracts from *Psiadia* species with those to all *P. dentata* extracts (Figure 5A). A similar analysis was performed by comparing all moderately active and inactive extracts to *P. dentata* extracts (Figure S1).

Examination of the contribution plot highlighted distinct signals in the following regions of the ^1^H NMR spectrum: the aliphatic region (0.69–1.93 ppm), the sugar-based/N-containing region (3.83–4.65 ppm), the olefinic region (5.49 ppm), and the aromatic region (6.4–8.0 ppm) of the ^1^H NMR spectrum. The ^1^H NMR spectra of the active ethyl acetate crude extracts of *P. dentata* were analyzed, and the chemical shifts of interest were compared with all compounds previously identified within the genus *Psiadia*, based on our earlier review of this genus [15]. This comparison enabled the assignment of specific spectral variables (1.77, 1.81, 3.93, 4.65, 5.49, and 6.45 ppm) to the chemical shifts of the compound isoobtusitin (**18**) (Figure 5B,C). All the chemical shifts corresponding to isoobtusitin were identified in the ^1^H NMR spectrum of the most active extract of *P. dentata*.

Further analysis of the ^1^H NMR spectrum revealed additional signals at δ_H_ 6.29 (d, *J* = 2.1 Hz), δ_H_ 6.41 (d, *J* = 2.1 Hz), δ_H_ 6.93 (d, *J* = 9.0 Hz) and δ_H_ 8.00 (d, *J* = 9.0 Hz) which may correspond to flavonols. In addition, the latter signals at δ_H_ 3.85 (s) and 3.90 (s) suggested the presence of two specific flavonols in the active crude extract: ermanin (**1**) and isokaempferide (**7**). These three molecules had previously been isolated from *P. dentata* crude extract [16,17]. Their higher abundance in the most active extract correlates with the observed antiplasmodial activity, suggesting a potential contribution, although the participation of other minor or as-yet unidentified compounds cannot be ruled out. Moreover, these molecules were also detected in the moderately active extract, at lower concentrations. Indeed, the concentrations of ermanin and isokaempferide are 5 times lower in the moderately active extract (collected at Dos d’Ane, IC_50_ = 27.7 µg/mL), while the concentration of isoobtusitin is 8 times lower (Figure S2). Several studies have reported that flavonoids and coumarins exhibit antimalarial activity [36,37,38,39]. Furthermore, signals observed in the aliphatic region also appeared to play a significant role in the discrimination between active and inactive extracts. However, precise identification of these was not possible due to overlapping peaks in this spectral region, suggesting that additional, yet unidentified, compounds may contribute to the observed bioactivities.

#### 2.3.2. PLS-DA of ^1^H NMR Data of *P. dentata* Crude Extracts and Correlation with the Anti-Inflammatory Activity

**Figure 6:**
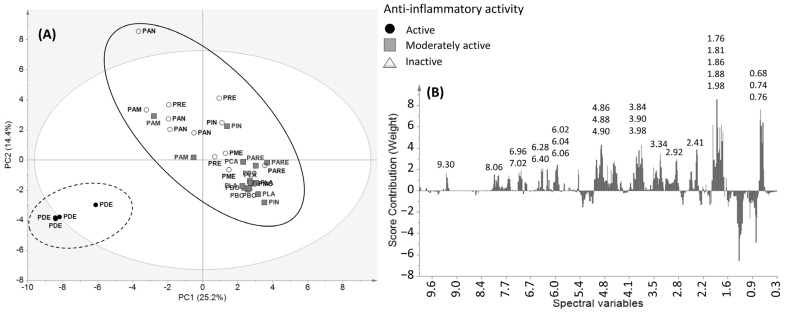
(**A**) Score plot of PLS-DA model generated from the ^1^H NMR spectra (600 MHz) of *Psiadia* species crude extracts for the anti-inflammatory activity. (**B**) Contribution plot of the PLS-DA model of *P. dentata* active crude extracts against *Psiadia* sp. inactive and moderately active extracts. Plant codes: PAM (*P. amygdalina*), PAN (*P. anchusifolia*), PARE (*P. argentea*), PBO (*P. boivinii*), PCA (*P. callocephala*), PDE (*P. dentata*), PIN (*P. insignis*), PLA (*P. laurifolia*), PMO (*P. montana*), PME (*P. melastomatoides*), PRE (*P. retusa*).

The correlation between the anti-inflammatory activity of *P. dentata* crude extracts and their chemical composition was analyzed using an OPLS-DA model. The model exhibited the following statistical parameters: R^2^ (X) = 0.39, R^2^ (Y) = 0.56, and Q^2^ = 0.38. The model revealed two distinct groups: the first group consisted of active crude extracts of *P. dentata*, while the second group included all inactive and moderately active extracts (Figure 6). Therefore, a contribution plot was generated by comparing the spectral variables of all active extracts (group 1) with those of inactive and moderately active extracts (group 2). Examination of the contribution plot highlighted signals in the following regions of the ^1^H NMR spectrum: the aliphatic region (0.69–1.93 ppm), the sugar-based/N-containing (3.83–4.65), and the aromatic region (6.4–8.0 ppm). Comparison of the chemical shifts with all compounds previously identified within the genus *Psiadia* allowed the attribution of spectral variables at 1.81, 3.84, 6.28, 6.40, 7.02, and 8.06 ppm to the compound isokaempferide (**7**). However, other spectral variables highlighted by the contribution plot could not be attributed to known compounds detected in *Psiadia* species. A more comprehensive study on the chemical composition of *P. dentata* active crude extract is required to identify the other potential anti-inflammatory compounds.

### 2.4. Phytochemical Investigation

**Figure 7:**
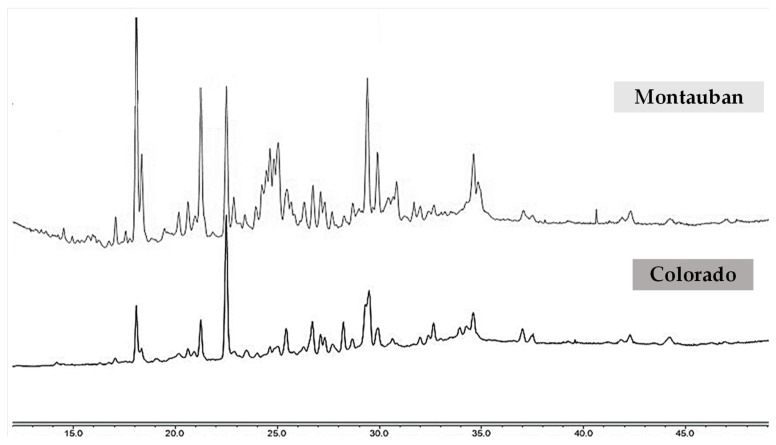
HPLC-CAD chromatograms of two bioactive extracts of *P. dentata*.

Among the four EtOAc extracts of *P. dentata* collected in summer and evaluated for their biological activities (Section 2.1), two extracts from Colorado and Montauban exhibited the highest antiplasmodial (IC_50_ = 8.4 and 14.4 μg/mL respectively) and anti-inflammatory activities (IC_50_ = 3.9 and 9.6 μg/mL respectively). Comparative chemical profiling using HPLC-CAD (high-performance liquid chromatography coupled with charged aerosol detection) revealed that both extracts shared broadly similar chromatographic profiles, suggesting the presence of related compounds. However, Montauban extract exhibited a slightly richer chromatographic profile (Figure 7) and was selected for subsequent fractionation and purification.

The selected EtOAc leaf extract of *P. dentata* was subjected to liquid–liquid partitioning using methanol (MeOH) and isohexane, yielding two fractions: a hydrocarbon fraction and an oxygenated fraction. These two fractions were then evaluated for their biological activities, including anti-inflammatory and cytotoxic properties. Both fractions exhibited anti-inflammatory effects, with IC_50_ values for NO release of 0.04 and 0.05 µg/mL, respectively. They also showed cytotoxic effects against HepG2 hepatocarcinoma cells (IC_50_ = 18.65 and 15.94 µg/mL, respectively) and HT-29 colorectal cancer cells (IC_50_ = 18.63 and 10.38 µg/mL, respectively). Interestingly, the crude EtOAc extract was more cytotoxic than either fraction, with IC_50_ values of 8.64 and 6.34 µg/mL, against HepG2 and HT-29 cells, respectively. This difference may be attributed to synergistic interactions between compounds in the crude extract that are lost after fractionation.

**Figure 8:**
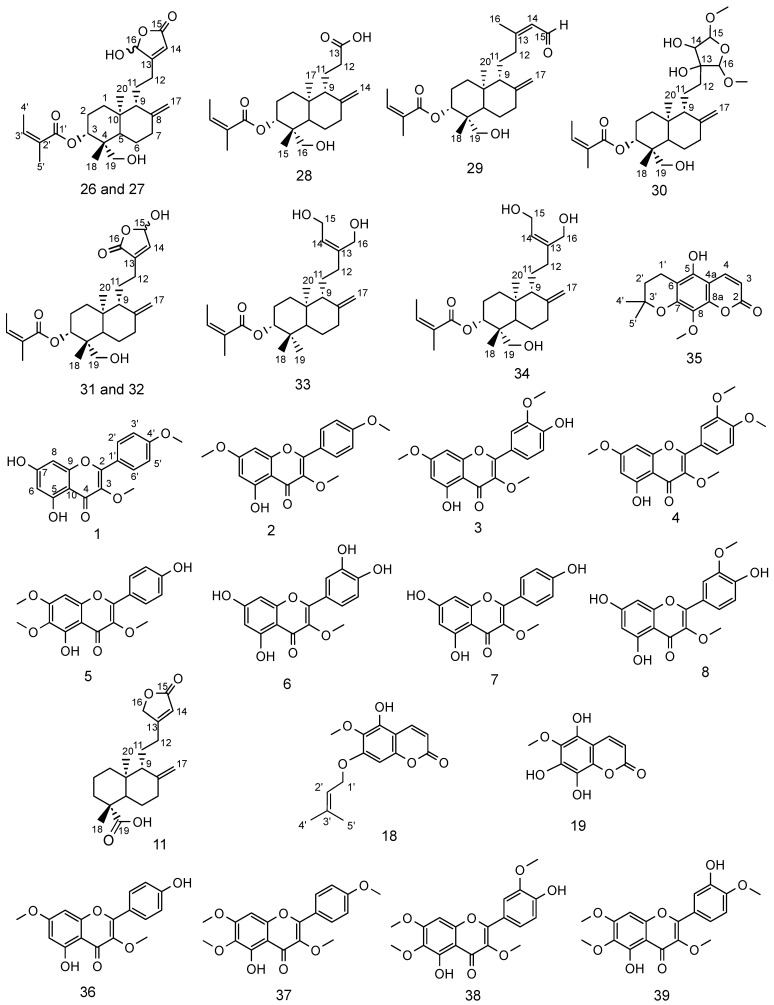
Structures of new compounds **26**–**35** and known compounds **1**–**8**, **11**, **18**, **19** and **36**–**39** isolated from *P. dentata*.

Both fractions were further subjected to purification of their compounds using a combination of normal-phase medium-pressure liquid chromatography (MPLC) and repeated reversed-phase semi-preparative HPLC. This process led to the isolation of nine new diterpenes (**26**–**34**), one coumarin (**35**), and fifteen known compounds (**1**–**8**, **11**, **18**, **19** and **36**–**39**) (Figure 8). The latter were identified by comparison with previously reported spectroscopic data as ermanin (**1**) [41], kaempferol 3,7,4′-trimethylether (**2**) [42], patchypodol (**3**) [43], retusin (**4**) [44], penduletin (**5**) [45], quercetin 3-methylether (**6**) [46], isokaempferide (**7**) [40], quercetin 3,3′-dimethylether (**8**) [47], *ent*-8(17),13-labdadien-15,16-olid-18-oic acid (**11**) [48], isoobtusitin (**18**) [17], fratexin (**19**) [49], kumatakilin (**36**) [50], penduletin 4-methylether (**37**) [51], chrysosplenetin (**38**) [52] and casticin (**39**) [53]. The structure of the new compounds **26**–**35** was established by the analysis of their UV, IR, NMR (^1^H and ^13^C NMR, COSY, HSQC, HMBC and NOESY) spectroscopic data, together with HRESIMS spectrometric data.

Interestingly, the isolated metabolites are consistent with the molecular network analysis, which had already highlighted the presence of flavonoids, coumarins, and diterpenoids in this species. Particularly, the identification of known diterpene *ent*-8(17),13-labdadien-15,16-olid-18-oic acid (**11**), together with the coumarins isoobtusitin (**18**), and fratexin (**19**), as well as several flavonoids including ermanin (**1**), kaempferol 3,7,4′-trimethylether (**2**), patchypodol (**3**), retusin (**4**), penduletin (**5**), quercetin 3-methylether (**6**), isokaempferide (**7**) and quercetin 3,3′-dimethylether (**8**), provides experimental confirmation of the chemical diversity predicted by the MN approach.

#### 2.4.1. Structural Elucidation of Compounds **26**–**35**

**Figure 9:**
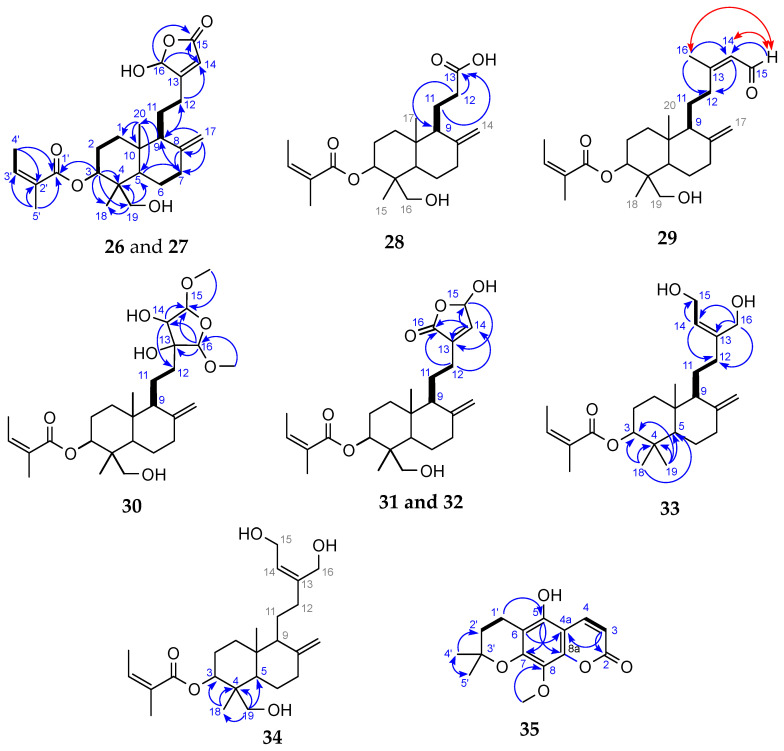
Key COSY (**bold** chain indicates vicinal proton correlations only), NOE (red arrows) and HMBC (blue arrows, H → C) correlations for compounds **26**–**35**.

Compounds **26** and **27** were isolated as yellow powders in 1:1 mixture. Their molecular formula C_25_H_36_O_6_ was determined by HRESIMS, which showed a [M + H]^+^ ion at *m*/*z* 433.2587 (calcd. for C_25_H_37_O_6_ [M + H]^+^, 433.2585), corresponding to 8 degrees of unsaturation. The UV spectrum displayed an absorption band typical of conjugated double bonds at λ_max_ 225 nm. In the IR spectrum, characteristic vibration bands were observed at 3371 cm^−1^ (O–H stretching) 2925 cm^−1^ and 2853 cm^−1^ (CH2 asymmetric and symmetric stretching); 1757 cm^−1^ (conjugated C=O), 1646 cm^−1^ (conjugated C=C) and 1260 cm^−1^ (C–C stretching) (Figures S3–S5). Analysis of the ^13^C NMR spectrum (Figure S7) revealed 10 characteristic carbon signals of a labdane-type diterpenoid: C-1 (δc 36.8), C-2 (δ_C_ 24.4), C-3 (δ_C_ 74.3), C-4 (δ_C_ 42.9), C-5 (δ_C_ 46.0 and 46.1), C-6 (δ_C_ 23.3), C-7 (δ_C_ 37.6), C-8 (δ_C_ 147.2 and 147.3), C-9 (δ_C_ 55.9 and 56.1) and C-10 (δ_C_ 39.2). The duplication of several carbon signals confirmed that the sample contained two diastereoisomers in a 1:1 ratio, as further supported by their relative peak intensities. The ^1^H NMR data (Table 2 and Figure S6) corroborated this structure framework, showing characteristic resonances for H-1 (δ_H_ 1.29 and 1.82), H-2 (δ_H_ 1.73 and 1.91), H-3 (δ_H_ 4.97), H-5 (δ_H_ 1.74), H-6 (δ_H_ 1.37 and 1.75), H-7 (δ_H_ 2.08 and 2.39) and H-9 (δ_H_ 1.75). The deshielded signal of H-3 (δ_H_ 4.97) indicated its attachment to an oxygen atom. The diterpene was methyl-substituted at C-10 and disubstituted at C-4 [54]. Moreover, broad singlets at δ_H_ 4.51 and 4.89 (H-17) indicated a methylidene group. Additional resonances at δ_H_ 1.98 (H-4′), 1.87 (H-5′), and 6.12 (H-3′) supported the presence of an angeloyl group [55,56]. COSY and HMBC experiments confirmed this assignment (Figures S8 and S10): COSY correlations were observed between H-4′ and H-3′, while HMBC correlations of H-5′ with C-1′ (δ_C_ 169.3), C-2′ (δ_C_ 127.7), and C-3′ (δ_C_ 139.5), and similarly for H-4′ indicated that the angeloyl group was esterified at C-3, by the presence of HMBC correlations of H-3 with C-1′ (δ_C_ 169.3). Further HMBC correlations of H-3 with C-18 (δ_C_ 13.0) and C-19 (δ_C_ 64.5), and of H-18 (δ_H_ 0.68) and H-19 (δ_H_ 2.91, 3.36) with C-4 (δ_C_ 42.9) indicated their spatial proximity. The deshielding of C-19 (δ_C_ 64.5) and its attached protons (δ_H_ 2.91; 3.36) suggested the presence of a neighbouring oxygen atom (Figure 9). HMBC correlations of H-17 with C-7 (δ_C_ 37.6), C-8 (δ_C_ 147.3), and C-9 (δ_C_ 56.1) confirmed the attachment of the methylidene group at C-8. HMBC correlations between H-14 (δ_H_ 5.86) and C-15 (δ_C_ 170.2)/C-16 (δ_C_ 99.4), as well as between H-16 (δ_H_ 5.97) and C-14 (δ_C_ 117.3)/C-15 (δ_C_ 170.2), established the presence of a *β*-substituted butenolide ring. The chemical shift of C-16 further supported the presence of a hydroxyl group at this position. The ^1^H NMR spectrum also showed methylene signals at δ_H_ 1.68–1.80 (H-11) and δ_H_ 2.34–2.52 (H-12). COSY correlations between H-9 (δ_H_ 1.75) and H-11, as well as between H-11 and H-12, indicated the sequential connectivity of C-9, C-11, and C-12. Moreover, HMBC correlations between H-12 and C-14 (δ_C_ 117.3) confirmed the hydroxybutenolide ring was attached to C-12 (Figure 9). The duplicated ^13^C signals were mainly associated with the butenolide moiety, suggesting that compounds **26** and **27** are diastereoisomers differing only at the chiral centre C-16 (*R* or *S* hydroxyl) (Figure S7). Altogether, these data allowed the identification of compounds **26** and **27** as 3α-angeloyloxy-19,16(*R*/*S*)-dihydroxy-*ent*-labda-8(17),13-dien-15,16-olide. Their ^1^H and ^13^C NMR data closely resembled those of 3α-angeloyloxy-19-hydroxy-*ent*-labda-8(17),13-dien-15,16-olide, previously isolated from *Gutierrezia solbrigii* (*Asteraceae*) [57]. However, the main difference lies at the C-16 position, where compounds **26** and **27** bear a hydroxyl group, whereas the reported diterpene contains a methylene group. The stereochemistry of these compounds and of the remaining eight new diterpenes, is discussed further in Section 2.4.2.

Compound **28** was obtained as a white paste. Its molecular formula C_22_H_34_O_5_ was determined by HRESIMS, which showed a [M + H]^+^ ion at *m*/*z* 379.2486 (calcd. for C_22_H_35_O_5_^+^, 379.2400), corresponding to 6 degrees of unsaturation. The UV spectrum displayed an absorption band at λ_max_ 199 nm, attributable to a carbonyl group. The IR spectrum showed absorption bands at 3305 cm^−1^ (O–H stretching), 2963 cm^−1^ (CH_3_ asymmetric vibrations), 2923 cm^−1^ and 2853 cm^−1^ (CH2 asymmetric and symmetric vibrations), 1715 cm^−1^ (C=O), and 1261 cm^−1^ (C–C stretching) (Figures S12–S14). The ^1^H, COSY, HSQC and HMBC NMR data (Figures S15–S18) of **28** were highly similar to those of compounds **26** and **27** (Table 2 and Table 4), indicating a labdane skeleton substituted at C-3 by an angeloyl group, at C-4 (δ_C_ 43.5) by a methyl and a hydroxymethyl group, at C-8 (δ_C_ 147.4) by an exocyclic methylene, and at C-10 (δ_C_ 39.2) by a methyl group. The main difference between compound **28** and the two diastereoisomers **26** and **27** was located at C-12. Specifically, the chemical shift of C-13 (δ_C_ 177.2) in compound **28** indicated the presence of a carbonyl function. In the HMBC spectrum, correlations of H-11 (δ_H_ 1.68, 1.90) and H-12 (δ_H_ 2.32, 2.53) with C-13 supported the attachment of a carboxylic acid group at C-12, as depicted in Figure 9. Accordingly, compound **28** was identified as 3α-angeloyloxy-16-hydroxy-*ent*-labda-8(17)-en-13-oic acid.

Compound **29** was isolated as a white oil with HRESIMS analysis indicating an [M + NH_4_]^+^ peak at *m*/*z* 420.3108 (calcd. for C_25_H_42_O_4_N^+^, 420.3109), corresponding to 7 degrees of unsaturation. The UV spectrum of compound **29** revealed the same absorption maximum at λ_max_ 227 nm as observed for compounds **26** and **27**. Its IR spectrum showed close similarities to those of **26** and **27**: absorption bands were observed at 3394 cm^−1^ (O–H stretching), 2961.09 cm^−1^ (CH_3_ asymmetric vibrations), 2924 cm^−1^ and 2854 cm^−1^ (CH2 asymmetric and symmetric vibrations), 1733 cm^−1^ (conjugated C=O), 1673 cm^−1^ (conjugated C=C), 1260 cm^−1^ (C–C stretching), 1022 cm^−1^ (C–O), and 798 cm^−1^ (aromatic C–H) (Figures S20–S22). The ^1^H and ^13^C NMR spectroscopic data determined with edited HSQC and HMBC spectra (Table 2 and Table 4) were closely related to those of compounds **26** and **27**, except for the moiety attached to carbon C-12. The ^1^H NMR spectrum (Figure S23) showed additional signals corresponding to the methyl protons H-16 (δ_H_ 2.22), a deshielded aldehydic proton H-15 (δ_H_ 9.96), and an olefinic proton H-14 (δ_H_ 5.83, *J* = 8.2 Hz) coupled to the aldehydic proton H-15. HMBC correlations of H-16 (δ_H_ 2.22) with C-12 (δ_C_ 40.5), C-14 (δ_C_ 128.1), and C-15 (δ_C_ 193.6), as well as correlations of H-14 (δ_H_ 5.83) with C-12 (δ_C_ 40.5) and C-16 (δ_C_ 17.6); and of H-15 (δ_H_ 9.96) with C-14 (δ_C_ 128.1) established the carbon chain motif (Figure 9). Thus, compound **29** was identified as 3α-angeloyloxy-19-hydroxy-*ent*-labda-8(17),13*E*-dien-15-al.

Compound **30** was purified as a white paste with HRESIMS analysis indicating [M – H]^−^ ion peak at *m*/*z* 495.2049 (calcd. for C_27_H_43_O_8_^−^, 495.2963), accounting for 6 degrees of unsaturation. Inspection of its UV spectrum indicated an absorption band at λ_max_ 197 nm, attributable to a carbonyl group. The IR spectrum displayed absorption bands at 3467 cm^−1^ (O–H stretching), 2953 cm^−1^ (asymmetric CH_3_ vibrations), 2929 cm^−1^ and 2854 cm^−1^ (asymmetric and symmetric CH_2_ vibrations), 1692 cm^−1^ (conjugated C=O), 1644 cm^−1^ (conjugated C=C), 1260 cm^−1^ (C–C stretching), and 1022 cm^−1^ (C–O) (Figures S28–S30). The ^1^H and ^13^C NMR spectral data (Table 2 and Table 4) of compound **30** closely resembled those of compounds **26**–**29**, consistent with a labdane skeleton substituted at C-4 by a methyl and hydroxymethyl group, at C-3 by an angeloyl group, at C-8 by an exocyclic methylene, and with a linear connectivity between C-9, C-11 and C-12. Moreover, three deshielded signals H-14 (δ_H_ 3.95), H-15 (δ_H_ 4.90), and H-16 (δ_H_ 4.75) suggested proximity to oxygenated functionalities. The chemical shift of C-14 (δ_C_ 80.4) further indicated a hydroxyl substitution. Two methoxy groups at δ_H_ 3.47 and δ_H_ 3.40 were attached to carbons C-15 and C-16, respectively. Thus, compound **30** was characterized as 3α-angeoyloxy-19-hydroxy-15,16-dimethoxy-*ent*-labda-8(17)-en-furan-13,14-diol.

Compounds **31** and **32** were isolated as white paste in 1:1 mixture with HRESIMS analysis indicating [M + H]^+^ peak at *m*/*z* 433.2589 (calcd. for C_25_H_37_O_6_^+^, *m*/*z* 433.2585), accounting for 8 degrees of unsaturation. The UV spectrum displayed a maximum absorption at λ_max_ 197 nm, identical to that of **30**. The IR spectrum and NMR spectra indicated marked structural similarities with compounds **26** and **27** (Figures S37–S39), with the main differences observed in the butenolide moiety. In fact, HMBC correlations between the olefinic proton H-14 (δ_H_ 6.85) and carbons C-13 (δ_C_ 138.6), C-15 (δ_C_ 96.9), and C-16 (δ_C_ 171.8), together with the correlations between H-15 (δ_H_ 6.08) and carbons C-13 (δ_C_ 138.6) and C-16 (δ_C_ 171.8), supported the presence of an *α*,*β*-unsaturated *γ*-lactone ring [58]. In addition, HMBC correlations from protons H-12 (δ_H_ 2.16 and 2.46) to carbons C-14 (δ_C_ 143.6) and C-16 (δ_C_ 171.8) confirmed the attachment of this lactone to C-12 (Figure 9). The downfield chemical shift of C-15 (δ_C_ 96.9) further suggested a hydroxyl substitution at this position. The duplicated signals observed in the ^13^C NMR spectrum in the lactone moiety supported the presence of two diastereoisomers, differing solely in the stereochemical configuration of C-15 (*R*/*S* hydroxyl) (Figure S41). Based on these combined spectroscopic data of compounds **31** and **32** were thus identified as 3*α*-angeloyloxy-19,15(*R*/*S*)-dihydroxy-*ent*-andrograpanin.

Compound **33** was isolated as white crystals. Its molecular formula was determined by HRESIMS, which showed a [M + H]^+^ ion at *m*/*z* 427.2815 (calcd. for C_25_H_41_O_4_^+^, 427.2819), corresponding to 6 degrees of unsaturation. Inspection of its UV spectrum indicated similarities with those of **26** and **27**. Its IR spectrum exhibited similarities to that of compound **28**, with additional absorption bands at 1603 cm^−1^ (asymmetric C=C), 1456 cm^−1^ (asymmetric CH_3_), and 1384 cm^−1^ (symmetric CH_3_) (Figures S37–S39). The ^1^H and ^13^C NMR data from edited HSQC and HMBC spectra (Table 3 and Table 4) of compound **33** displayed the characteristic features in the other compounds: a labdane skeleton with an angeloyl group at C-3, an exocyclic methylene group at C-8, and a linear connectivity of C-9, C-11 and C-12. However, in the ^1^H NMR spectrum (Figure S49), several deshielded signals were observed: H-14 (δ_H_ 5.62), H-15 (δ_H_ 4.22), and H-16 (δ_H_ 4.16 and 4.20). The COSY correlation between H-14 and H-15 indicated that these protons are attached to vicinal carbons (Figure 9). Furthermore, the chemical shifts of C-15 (δ_C_ 59.1) and C-16 (δ_C_ 61.5) suggest that these carbons are bonded to oxygen atoms. In the HMBC spectrum (Figure S52), correlations were observed between H-14 and carbons C-12 (δ_C_ 35.3) and C-15 (δ_C_ 59.1), as well as between H-16 and carbons C-12, C-13 (δ_C_ 144.5), and C-14 (δ_C_ 126.6). Based on these data, compound **33** was identified as 3*α*-angeloyloxy-15,16-dihydroxy-*ent*-labda-8(17),13*E*-diene.

Compound **34** was isolated as white crystals. Its HRESIMS spectrum displayed a pseudomolecular ion [M + Na]^+^ at *m*/*z* 443.2764, consistent with the molecular formula (calcd. for C_25_H_40_O_5_Na^+^, 443.2768), corresponding to 6 degrees of unsaturation. The UV spectrum displayed an absorption band at λ_max_ 194 nm, similar to that of compound **28**, which may be attributed to the carbonyl group (Figures S53 and S54). Its IR and ^1^H NMR spectra (Figures S55 and S56) were very similar to those of compound **33**, except at the C-19 position. In compound **33**, H-19 appeared as a methyl singlet (δ_H_ 0.90), whereas in compound **34** two doublets (δ_H_ 2.92, 3.35) indicated the presence of a hydroxymethyl group. HMBC correlations between protons H-19 and carbons C-3 (δ_C_ 74.3), C-4 (δ_C_ 42.9), C-5 (δ_C_ 46.1), and C-18 (δ_C_ 13.1) confirmed the assignment of the hydroxymethyl group at C-4 (Figure 9). Compound **34** was therefore assigned as 3*α*-angeloyloxy-15,16,19-trihydroxy-*ent*-labda-8(17),13*E*-diene.

Compound **35** was obtained as a mixture with fraxetin (**19**) [49] as pale yellow powders. Its molecular formula was established by HRESIMS, which showed a [M + H]^+^ ion at *m*/*z* 277.1083 (calcd. for C_15_H_17_O_5_^+^, 277.1071), corresponding to 8 degrees of unsaturation. Based on the relative intensities of the split signals observed in the ^1^H NMR spectrum, the mixture ratio was estimated to be 65:35. The UV spectrum displayed an absorption band at λ_max_ 211 nm, which may be related to an aromatic or conjugated system. The IR spectrum showed characteristic absorption bands at 3293 cm^−1^ (O–H stretching), 2968 cm^−1^ (asymmetric CH_3_), 2933 cm^−1^ 2857 cm^−1^ (asymmetric and symmetric CH_2_ stretching), 1700 cm^−1^ (conjugated C=O), 1620 cm^−1^ (conjugated C=C), 1503 cm^−1^ (aromatic C=C), 1467 cm^−1^ (asymmetric CH_3_), 1377 cm^−1^ (symmetric CH_3_), 1181 cm^−1^ (isopropyl C–C), 1029–1143 cm^−1^ (C–O), and 804–927 cm^−1^ (aromatic C–H) (Figures S60–S62). The ^1^H NMR spectrum (Figure S63) revealed two doublets, H-3 (δ_H_ 6.13) and H-4 (δ_H_ 8.13), coupled with *J* = 9.6 Hz, indicating a 1,2-benzopyrone nucleus substituted at C-5 by an oxygen [59]. Additional signals included a singlet at δ_H_ 1.41 (H-4′ and H-5′) corresponding to two methyl groups, two triplets at δ_H_ 1.88 (H-2′) and δ_H_ 2.82 (H-1′) corresponding to two methylene groups, and a singlet at δ_H_ 3.78 representing a methoxy group, suggesting that compound **35** is a pyranocoumarin [60]. COSY correlations between protons H-3 (δ_H_ 6.13) and H-4 (δ_H_ 8.13), and between protons H-1′ (δ_H_ 2.82) and H-2′ (δ_H_ 1.88), indicated vicinal coupling. Furthermore, HMBC correlations between H-4′/H-5′ (δ_H_ 1.41) and C-2′ (δ_C_ 32.6), as well as between the proton signal at δ_H_ 3.78 and C-8 (δ_C_ 133.6), allowed the assignment of the two methyl groups at C-3′ and the methoxy group at C-8, respectively (Figure 9). Compound **35** was thus unambiguously identified as 5-hydroxydihydroluvangetin.

**Table 2:** ^1^H NMR data of compounds **26**–**30** (600 MHz, CDCl_3_, 300 K).

Position	26 and 27		28		29		30	
	δ_H_	(*J* in Hz)	δ_H_	(*J* in Hz)	δ_H_	(*J* in Hz)	δ_H_	(*J* in Hz)
1	1.29; 1.82	m; m	1.86; 1.34	m; m	1.29; 1.85	m; m	1.28; 1.82	m; m
2	1.73; 1.91	m; m	1.73; 1.90	m; m	1.75; 1.80	m; m	1.70; 1.90	m; m
3	4.97	-	5.01	dd (12.2, 4.3)	4.97	dd (12.0, 4.5)	5	dd (12.1, 4.5)
4	-	-	-	-	-	-	-	-
5	1.74	m	1.76	m	1.73 (m)	m	1.74	m
6	1.37; 1.75	m; m	1.37; 1.75	m; m	1.75 (m)	m	1.37; 1.74	m; m
7	2.08; 2.39	m; m	2.07; 2.39	m; m	2.07; 2.40	m; m	2.06; 2.38	brtd (12.9, 4.6); ddd (12.9, 4.2, 2.3)
8	-	-	-	-	-	-	-	-
9	1.75	m	1.73	m	1.71	m	1.66	m
10	-	-	-	-	-	-	-	-
11	1.68; 1.80	m; m	1.68; 1.90	m; m	1.62; 1.76	m; m	1.73; 1.90	m; m
12	2.34; 2.52	m; m	2.32; 2.53	m; m	2.13; 2.41	m; m	1.46; 1.82	m; m
13	-	-	-	-	-	-	-	-
14	5.86	s	4.52; 4.88	brs; brs	5.83	dsxt (8.2, 1.2)	3.95	brd (3.6)
15	-	-	0.69	s	9.96	d (8.2)	4.9	brd (3.6)
16	5.97	s	2.92; 3.35	d (12.6); d (12.6)	2.22	d (2.2)	4.75	s
17	4.51; 4.89	brs; brs	0.77	s	4.57; 4.92	brd (1.2); brs	4.58; 4.84	brs; brs
18	0.68	s	-	-	0.76	s	0.68	s
19	2.91; 3.36	d (12.5); d (12.7)	-	-	3.13; 3.30	brd (11.6); brd (11.6)	2.92; 3.34	brd (12.5); brd (12.5)
20	0.77	s	-	-	0.81	s	0.76	s
1′	-	-	-	-	-	-	-	-
2′	-	-	-	-	-	-	-	-
3′	6.12	ddq (14.7, 7.3, 1.5)	6.12	qq (12.1, 1.5)	6.09	brqq (7.2, 1.4)	6.11	brqq (7.2, 1.4)
4′	1.98	dq (7.3, 1.4)	1.99	dq (7.0, 1.5)	1.96	brdq (7.2, 1.4)	1.99	brdq (7.2, 1.4)
5′	1.87	m	1.88	m	1.87	brqt (1.4)	1.88	brqt (1.4)
HO-13	-	-	-	-	-	-	2.44	brs
HO-14	-	-	-	-	-	-	2.56	brs
CH_3_O-15	-	-	-	-	-	-	3.47	s
CH_3_O-16	-	-	-	-	-	-	3.4	s

br: broad (large signal).

**Table 3:** ^1^H NMR data of compounds **31**–**35** (600 MHz, CDCl_3_, 300 K).

Position	31 and 32		33		34		35	
	δ_H_	(*J* in Hz)	δ_H_	(*J* in Hz)	δ_H_	(*J* in Hz)	δ_H_	(*J* in Hz)
1	1.26; 1.82	m; m	1.25; 1.81	m; m	1.28; 1.84	m; m	-	-
2	1.71; 1.89	m; m	1.65; 1.80	m; m	1.72; 1.91	m; m	6.13	d (9.6)
3	4.97	dt (11.9, 4.5)	4.99	dd (11.9, 4.0)	5.00	dd (12.0, 4.4)	8.13	d (9.6)
4	-	-	-	-	-	-	-	-
5	1.74	m	1.21	m	1.75	m	-	-
6	1.36; 1.73	m; m	1.41; 1.75	dd (12.9, 4.2); m	1.35; 1.76	dd (13.1, 4.2); m	-	-
7	2.08; 2.39	m; dm (12.4)	2.02; 2.41	m; m	2.08; 2.39	m; m	-	-
8	-	-	-	-	-	-	-	-
9	1.73	m	1.61	m	1.71	m	-	-
10	-	-	-	-	-	-	-	-
11	1.64; 1.74	m; m	1.52; 1.61	m; m	1.51; 1.64	m; m	-	-
12	2.16; 2.46	m; m	1.92; 2.33	m; m	1.92; 2.32	m; m	-	-
13	-	-	-	-	-	-	-	-
14	6.85	m	5.62	t (6.8)	5.62	t (7.1)	-	-
15	6.08	dt (6.3; 1.9)	4.22	dd (6.7, 2.9)	4.22	dd (6.8, 3.2)	-	-
16	-	-	4.16; 4.20	d (12.5); d (12.3)	4.16; 4.19	d (12.3); d (12.4)	-	-
17	4.58; 4.89	d (3.5); brs	4.56; 4.87	brs; brs	4.56; 4.87	brs; brs	-	-
18	0.68	s	0.88	s	0.68	s	-	-
19	2.91; 3.36	brd (12.6); brd (12.6)	0.9	s	2.92; 3.35	d (12.7); d (12.7)	-	-
20	0.75	s	0.72	s	0.76	s	-	-
1′	-	-	-	-	-	-	2.82	t (6.8)
2′	-	-	-	-	-	-	1.88	t (6.8)
3′	6.11	ddq (14.2, 7.1, 1.3)	6.03	qq (12.0, 1.5)	6.12	qq (12.1, 1.4)	-	
4′	1.98	dq (7.4; 1.6)	1.96	brdq (7.2, 1.5)	1.96	brdq (7.3, 1.4)	1.41	s
5′	1.88	m	1.89	m	1.88	m	1.41	s
CH_3_O-8	-	-	-	-	-	-	3.78	s

br: broad (large signal).

**Table 4:** ^13^C NMR data of compounds **26**–**35** (150 MHz, CDCl_3_, 300 K).

Position	26 and 27		28		29		30		31 and 32		33		34		35	
	δ_C_	Type	δ_C_ *	Type	δ_C_ *	Type	δ_C_	Type	δ_C_	Type	δ_C_ *	Type	δ_C_ *	Type	δ_C_ *	Type
1	36.8	CH_2_	36.9	CH_2_	37.6	CH_2_	36.8	CH_2_	36.8	CH_2_	37.3	CH_2_	36.1	CH_2_	-	-
2	24.4	CH_2_	24.3	CH_2_	24.8	CH_2_	24.5	CH_2_	23.3	CH_2_	25	CH_2_	24.4	CH_2_	163.9	C
3	74.3	CH	74.3	CH	75.5	CH	74.4	CH	74.4	CH	80.7	CH	74.3	CH	110.0	CH
4	42.9	C	43.5	C	43.1	C	43	C	42.9	C	38.5	C	42.9	C	141.6	CH
5	46.0; 46.1	CH	46.6	CH	47.3	CH	46.3	CH	46.1	CH	55.1	CH	46.1	CH	152.6	C
6	23.3	CH_2_	23.3	CH_2_	24.7	CH_2_	23.4	CH_2_	23.1	CH_2_	24.3	CH_2_	23.4	CH_2_	102.1	C
7	37.6	CH_2_	37.8	CH_2_	38.6	CH_2_	37.8	CH_2_	37.6	CH_2_	38.2	CH_2_	37.7	CH_2_	150.5	C
8	147.2; 147.3	C	147.4	C	148.9	C	147.8	C	147.2	C	148.2	C	147.2	C	133.6	C
9	55.9; 56.1	CH	55.8	CH	57.2	CH	57	CH	55.6; 55.7	CH	56.4	CH	56.1	CH	-	-
10	39.2	C	39.2	C	40.1	C	39.3	C	39.1	C	39.8	C	39.1	C	-	-
11	21.0; 21.2	CH_2_	19.1	CH_2_	22.6	CH_2_	24.5	CH_2_	21.6; 21.7	CH_2_	22.4	CH_2_	22.5	CH_2_	-	-
12	26.7; 26.9	CH_2_	32.4	CH_2_	40.5	CH_2_	32.8	CH_2_	24.3; 24.4	CH_2_	35.2	CH_2_	34.8	CH_2_	-	-
13	171.4	C	177.2	C	168	C	81.5	C	138.5; 138.6	C	144.5	C	144.5	C	-	-
14	117.3	CH	107.1	CH_2_	128.1	CH	80.4	CH	143.6	CH	126.6	CH	126.3	CH	-	-
15	170.1; 170.2	C	12.6	CH_3_	193.6	C	110.7	CH	96.9; 97.0	CH	59.1	CH_2_	58.8	CH_2_	-	-
16	99.0; 99.4	CH	64.5	CH_2_	17.6	CH_3_	109	CH	171.8; 172.0	C	61.5	CH_2_	61.2	CH_2_	-	-
17	107.1; 107.3	CH_2_	15.2	CH_3_	107.5	CH_2_	107.3	CH_2_	107.3	CH_2_	107.3	CH_2_	107	CH_2_	-	-
18	13	CH_3_	-	-	14	CH_3_	13	CH_3_	13	CH_3_	17.3	CH_3_	13.1	CH_3_	-	-
19	64.5	CH_2_	-	-	64.6	CH_2_	64.9	CH_2_	64.5	CH_2_	28.7	CH_3_	64.6	CH_2_	-	-
20	15.3	CH_3_	-	-	15.5	CH_3_	15.3	CH_3_	15.3	CH_3_	15.1	CH_3_	15.4	CH_3_	-	-
1′	169.3	C	169.3	C	169.1	C	169.2	C	169.3	C	168.2	C	169.1	C	17.1	CH_2_
2′	127.7	C	127.7	C	129.5	C	127.9	C	127.7	C	128.5	C	127.5	C	32.6	CH_2_
3′	139.5	CH	139.2	CH	138.8	CH	138.9	CH	139.3	CH	138.1	CH	139.1	CH	77.0	C
4′	16.1	CH_3_	16.1	CH_3_	16.1	CH_3_	16	CH_3_	16.1	CH_3_	16.2	CH_3_	16.2	CH_3_	27.0	CH_3_
5′	20.8	CH_3_	20.9	CH_3_	20.9	CH_3_	20.8	CH_3_	20.8	CH_3_	21.1	CH_3_	20.9	CH_3_	27.0	CH_3_
4a	-	-	-	-	-	-	-	-	-	-	-	-	-	-	103.9	C
8a	-	-	-	-	-	-	-	-	-	-	-	-	-	-	-	C
CH_3_O-8	-	-	-	-	-	-	-	-	-	-	-	-	-	-	61.4	CH_3_
CH_3_O-15	-	-	-	-	-	-	56.5	CH_3_	-	-	-	-	-	-	-	-
CH_3_O-16	-	-	-	-	-	-	55.4	CH_3_	-	-	-	-	-	-	-	-

* ^13^C chemical shifts determined by 2D NMR (edited-HSQC, HMBC spectra).

#### 2.4.2. Relative Configuration of Diterpenes **26**–**34**

The absolute configurations of the new diterpenes could not be determined due to the limited quantities available. Nevertheless, their relative configurations were established using Carman’s method [61]. Labdanes occur in nature as two antipodal groups of bicyclic molecules, commonly referred to as the “normal” and “*ent*” series, in which C-9 and C-10 adopt a *cis* configuration. Typically, compounds from the same series are found together, although co-occurrence of *normal*- and *ent*-labdanes has also been reported [61]. Optical rotation provides valuable insights into stereochemical assignment [62]. In labdane diterpenes, *normal*-labdanes typically show positive specific rotations [63,64], whereas *ent*-labdanes display negative values [64,65,66]. All newly isolated diterpenes reported herein exhibited negative rotations ([α]^25^_D_ −145 to −30), indicating that these compounds belong to the *ent*-labdane series, consistent with the known *ent*-labdane (**11**), *ent*-8(17),13-labdadien-15,16-olide-19-oic acid [48].

**Figure 10:**
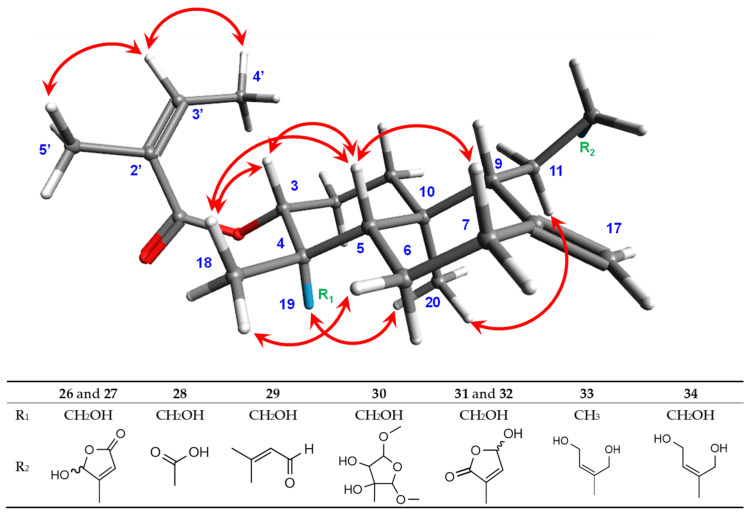
Key NOESY (red arrows) correlations observed for the decalin nucleus of compounds **26**–**34**.

The relative stereochemistry of these new diterpenes was further corroborated by NOESY analysis (Figures S11, S19, S27, S36 and S45). Within the bicyclic ring system, characteristic NOE correlations between H-3/H-5, H-18/H-5, and H-18/H-6 established the orientation of the C-18 methyl group, the C-6 methylene, and the axial protons H-3 and H-5. In derivatives bearing a hydroxymethyl group at C-19, correlations between H-19 and H-20 confirmed their co-facial arrangement, further supported by the H-11/H-20 interaction. Altogether, these data unambiguously establish the *ent*-configuration for the new labdanes (Figure 10). Additionally, NOE correlations between H-3′/H-4′ and H-3′/H-5′ in the angeloyl moiety allowed assignment of a (*Z*)-configuration for the double bond. However, the configurations of chiral centres C-13, C-15, and C-13′ located on the lateral chains could not be determined.

### 2.5. Biological Activities of the Isolated Compounds

#### 2.5.1. Antiplasmodial Activity of the Isolated Compounds

**Figure 11:**
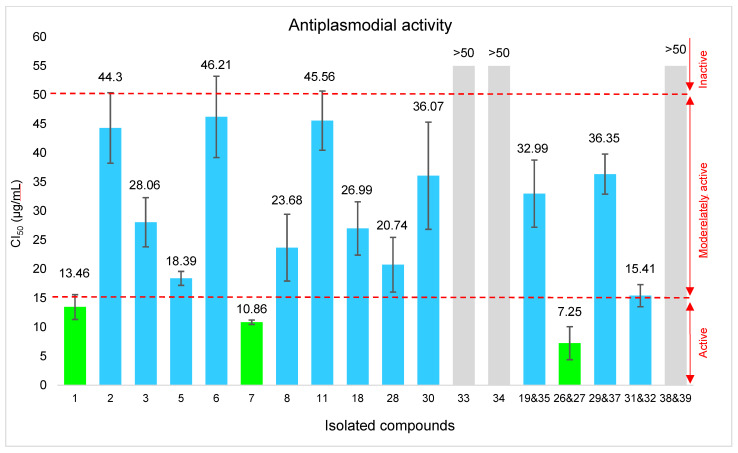
*In vitro* antiplasmodial activity (3D7 strain) of isolated compounds (IC_50_ in μg/mL). Positive control: artemisinin (IC_50_ = 0.003 ± 0.001 μg/mL). Active compound: 5–15 µg/mL, moderately active compound: 15–50 µg/mL, inactive compound (IC_50_ ≥ 50 μg/mL) [24,25].

The *in vitro* antiplasmodial activity of isolated compounds was evaluated, for the first time, against the *Plasmodium falciparum* 3D7 strain. The results are presented in Figure 11, showing the concentrations required to inhibit 50% of parasite growth (IC_50_). Three samples could not be tested due to limited availability: kumatakilin (**36**) (isolated in mixture with ermanin (**1**)), and the mixture of retusin (**4**) and penduletin 4-methylether (**37**).

Among the tested samples, according to classification [24,25] (active compound: 5–15 µg/mL), the mixture containing the newly identified compounds **26** and **27**, 3α-angeloyloxy-19,16(*R*/*S*)-dihydroxy-*ent*-labda-8(17),13-dien-15,16-olide, together with the known metabolites ermanin (**1**) and isokaempferide (**7**), displayed the highest inhibitory activity against *Plasmodium falciparum* within the series, with IC_50_ values ranging from 7.25 to 13.46 μg/mL. According to the same classification [24,25]), several other compounds (**2**, **3**, **5**, **6**, **8**, **11**, **18**, **28**, **30**, **19** and **35**, **29** and **37**, **31** and **32**) showed moderate activity, with IC_50_ values ranging from 15.41 to 46.21 μg/mL. Notably, the antiplasmodial effects of ermanin (**1**) (IC_50_ = 13.46 μg/mL) and quercetin 3-methylether (**6**) (IC_50_ = 46.21 μg/mL), have been previously reported [67,68], consistent with the current observations.

These results indicate that both flavonoid derivatives and some diterpenoid constituents may contribute to the antiplasmodial activity of *P. dentata*. However, although their IC_50_ values indicate inhibition of parasite growth, their potency remains considerably lower than that of the reference drug artemisinin (IC_50_ = 0.003 µg/mL). Therefore, they can be considered weak to moderately active and are not by themselves sufficient for high anti-malarial activity. Nevertheless, they may provide useful starting points for further optimization or structure–activity relationship studies.

Regarding the new compounds, labdane diterpenoids have been reported to exhibit moderate antiplasmodial activity *in vitro* [69,70,71]. The isolated diterpenes are quite similar, sharing a common *ent*-labdane skeleton featuring an angeloyl group at C-3 (except compound **11**), an exocyclic methylene group at C-8, and a linear connectivity between C-9, C-11, and C-12. They mainly differ in the functional groups extending from C-13. Taking their antiplasmodial activities into account, it is suggested that the hydroxyfuranone group in the mixtures of compounds **26** and **27** as well as **31** and **32** may improve the antiplasmodial activity, as these derivatives showed the highest activity within the tested series. These observations align with prior reports on angeloyloxylabdanes, where hydroxyfuranone substitution at C-13 enhances antiplasmodial potency [72]. In contrast, compounds **33** and **34** were inactive (IC_50_ ≥ 50 μg/mL), likely due to the presence of identical non-conjugated allylic diol functionalities at C-13. Compound **30** showed only weak activity, further supporting the contribution of the furanone moiety to the observed activity.

These results complement the metabolomic findings. Specifically, the antiplasmodial activities detected for isolated compounds such as ermanin (**1**), isokaempferide (**7**), and isoobtusitin (**18**), are consistent with the statistical correlations from metabolomic analysis, suggesting that these compounds, particularly isokaempferide (**7**), the major constituent of the extract may contribute to the antiplasmodial potential of *P. dentata*. Nevertheless, these compounds alone are unlikely to fully account for the activity of the crude extract. Minor active compounds could not be clearly identified due to signal overlap in the NMR spectra. In addition, signals detected in the aliphatic region, possibly corresponding to diterpenoids or other constituents, may also contribute to the overall antiplasmodial effect. The phytochemical investigation therefore complemented the metabolomic approach by enabling the isolation and identification of additional active constituents. Furthermore, the possible presence of chrysosplenol D (**10**), annotated in the molecular network and previously reported to possess antiplasmodial activity [73], may also play a role in the overall activity of the crude extract.

#### 2.5.2. Anti-Inflammatory Activity of the Isolated Compounds

**Figure 12:**
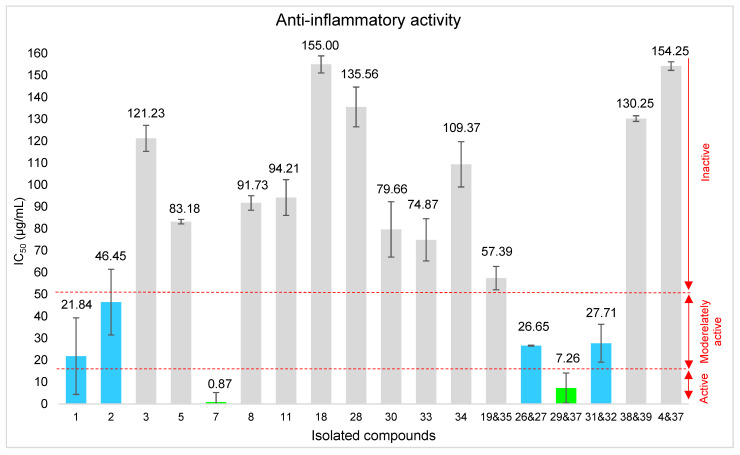
*In vitro* anti-inflammatory activity (RAW 264.7 cell line) of isolated compounds (IC_50_ in μg/mL). Positive control: dexamethasone (IC_50_ = 3.36 ± 1.04 μg/mL). Active compound: 5–15 µg/mL, moderately active compound: 15–50 µg/mL, inactive compound (IC_50_ ≥ 50 μg/mL) [24,25].

Isolated compounds were tested for *in vitro* anti-inflammatory activity by measuring the capacity of RAW 264.7 macrophages to generate an inflammatory response when stimulated with antigens, inducing nitric oxide (NO) release [74]. Due to limited availability, quercetin 3-methylether (**6**) and the sample with kumatakilin (**36**) were not evaluated. The results, presented in Figure 12, show the concentrations required to inhibit 50% of NO production (CI_50_).

Three tested compounds displayed a significant anti-inflammatory activity: the mixture containing the newly identified compound 3α-angeloyloxy-19-hydroxy-*ent*-labda-8(17),13*E*-dien-15-al (**29**) and penduletin 4-methylether (**37**), together with isokaempferide (**7**), inhibited NO production, with IC_50_ values of 7.26 and 0.87 μg/mL respectively (IC_50_ < 15 µg/mL, classification according to [24,25]). Meanwhile, the mixture containing the newly identified compounds **26** and **27**, 3α-angeloyloxy-19,16(*R*/*S*)-dihydroxy-*ent*-labda-8(17),13-dien-15,16-olide, compounds **31** and **32**, 3*α*-angeloyloxy-19,15(*R*/*S*)-dihydroxy-*ent*-andrograpanin, as well as ermanin (**1**) and kaempferol 3,7,4′-trimethylether (**2**) displayed moderate activity, with IC_50_ values ranging from 21.84 to 46.45 μg/mL (IC_50_ < 50 µg/mL, classification according to [24,25]). Their cytotoxicity (IC_50_) towards mouse macrophages was also evaluated. Among the active compounds, **29** and **37** showed significant cytotoxicity with IC_50_ of 22.27 μg/mL, whereas the remaining compounds exhibited IC_50_ values above 250 μg/mL, indicating low toxicity. Consequently, the mixture containing **29** and **37** cannot be considered a promising anti-inflammatory agent due to its cell cytotoxicity. The observed activities of ermanin (**11**) (IC_50_ = 21.84 μg/mL) and isokaempferide (**21**) (IC_50_ = 0.87 μg/mL) are consistent with previous reports [75,76], supporting their contribution to the anti-inflammatory potential of *P. dentata*.

Several labdane diterpenoids have been shown to inhibit NO production in RAW264.7 cells [77,78,79], which supports the present findings. However, the underlying mechanisms remain only partially elucidated. Analysis of the diterpenoid tested in the present study suggests that the anti-inflammatory effects of epimeric mixtures **26** and **27** as well **31** and **32** might be associated with the presence of a hydroxyfuranone moiety at C-13. This hypothesis is further supported by previous studies [77,78], in which several labdane-type diterpenoids featuring furanone derivative showed significant NO inhibition.

The metabolomic analysis revealed signals corresponding to isokaempferide that may be positively correlated with the anti-inflammatory activity of the crude extract. These results illustrate the ability of metabolomic profiling to link chemical composition with biological activity and the subsequent isolation and biological evaluation of isokaempferide provide experimental support for this prediction. However, since most isolated compounds were inactive or only moderately active, the bioactivity of the crude extract may be influenced by synergistic or matrix effects that are lost upon isolation, or by other compounds that were not isolated but may contribute to this activity.

#### 2.5.3. Cytotoxic Activity of the Isolated Compounds

**Figure 13:**
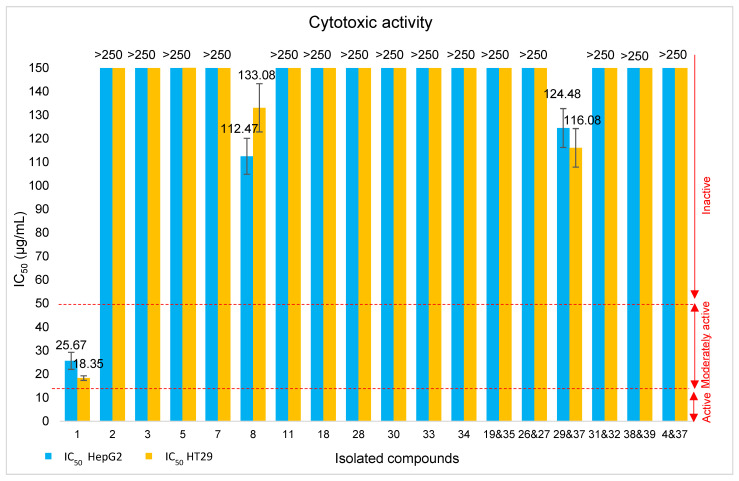
*In vitro* cytotoxic activity (HepG2 and HT29 cancer cell lines) of isolated compounds (IC_50_ in μg/mL). Active compound: 5–15 µg/mL, moderately active compound: 15–50 µg/mL, inactive compound (IC_50_ ≥ 50 μg/mL) [24,25].

The isolated compounds were tested for cytotoxic activity against human liver carcinoma (HepG2) and human colon and colorectal adenocarcinoma (HT29) cell lines, using the red dye assay. Due to limited availability, quercetin 3-methylether (**6**) and the mixture with kumatakilin (**36**) were not tested. The results shown in Figure 13 indicate the concentrations required to inhibit 50% of cell viability (IC_50_). Among the tested compounds, only ermanin (**1**) exhibited moderate cytotoxicity, with IC_50_ values of 25.67 and 18.35 μg/mL against the HepG2 and HT29 cell lines, respectively. This activity is consistent with previous reports, where the compound also exhibited cytotoxic effects against breast cancer (MCF-7) and oral cancer (BHY) cell lines [80,81].

## 3. Materials and Methods

### 3.1. General Experimental Procedures

Optical rotations [α]_D_ were carried out on an Anton Paar MCP 200 polarimeter (Anton Paar, Graz, Austria) at 25 °C, at 589 nm wavelength, with MeOH used as solvent, *c* (g/100 mL), in a 10 × 5 mm i.d., 0.2 mL sample cell. UV spectra were acquired on a Thermo Scientific DAD spectrophotometer (Thermo Scientific, Waltham, MA, USA). IR spectra were collected on an ATR-FTIR Bruker Tensor 27 spectrometer (Bruker, Billerica, MA, USA) with OPUS 7.2 software. The ^1^H NMR spectra for metabolomic study were acquired at 298 K on a Bruker Avance III 600 MHz NMR spectrometer (Bruker, Billerica, MA, USA) equipped with a TCI Cryoprobe and operated at 600.13 MHz for ^1^H. The 1D (^1^H and ^13^C) and 2D (COSY, HSQC, HMBC and NOESY) NMR spectra for compound identification were acquired at 300 K on a Bruker Avance II+ 600 MHz NMR spectrometer (Bruker, Billerica, MA, USA) equipped with a TCI Cryoprobe and operated at 600.13 MHz for ^1^H and 150 MHz for ^13^C. MS/MS spectra were recorded on a UHPLC system (Dionex Ultimate 3000, Thermo Scientific, Carlsbad, CA, USA) coupled to a high-resolution mass spectrometer HRMS-QTOF Impact II equipped with an electrospray ionization source (Bruker Daltonics, Bremen, Germany) in positive and negative ionization modes (20 eV and 40 eV). The following parameters were used: dry gas (N_2_): 4.00 L.min^−1^; nebulizer (N_2_) 3.5 bars; full-scan mode: *m*/*z* 50–1200; capillary voltage: 3.0 kV, Luna C18 reversed phase column, 1.6 μm, 150 × 2.1 mm i.d. (Phenomenex, Torrance, CA, USA). MPLC separations were carried out in normal phase on Buchi Sepacore flash systems C-605/C-615/C-660. A glass column (230 × 15 mm i.d.) packed with silica gel (40–63 µm). TLC was performed in normal phase on aluminum analytical plates precoated with 60 Å silica gel impregnated with a UV 254 nm fluorescence indicator. Spots were visualized on the basis of the UV absorbance at 254 nm and by heating silica gel plates sprayed with vanillin-sulphuric acid reagent. Analytical HPLC was carried out using a Gemini C18 (150 × 4.6 mm i.d., 3 µm) column (Phenomenex, Torrance, CA, USA) and was performed on a Thermo Scientific Dionex Ultimate 3000 system (Thermo Scientific, Waltham, MA, USA) equipped with a DAD photodiode array detector and a charged aerosol detector (CAD) with Chromeleon 7.2.8 software. Semi-preparative HPLC was carried out using a Gemini C18 (250 × 4.6 mm i.d., 5 µm) column (Phenomenex, Torrance, CA, USA) and was performed on a Thermo Scientific Dionex Ultimate 3000 system equipped with a DAD photodiode array detector or a Waters 2545 autopurification system process via MassLynx 4.2 software and coupled in series with a DAD photodiode array detector (190–800 nm, Waters 2998) and a DEDL light-scattering evaporative detector (Waters 2424) or a Agilent Technologies 1100 series (Agilent, Waldbronn, Germany) equipped with a DAD photodiode array detector and processed by Chemstation B.04.03 software. All solvents were analytical or HPLC grade and were used without further purification.

### 3.2. Plant Material

*Psiadia* species leaves (phylum Tracheophyta, class Magnoliopsida, order Asterales, family *Asteraceae*) were collected in 2015 in several collection sites on Reunion Island. Each species was collected in summer. A voucher specimen was deposited at the Herbarium of the University of Reunion Island for identification (identification performed by Jacques Fournel, Herbarium of the University of La Réunion). The collection sites and voucher numbers of all species are reported in Table 5.

### 3.3. Extraction

Prior to extraction, the leaves collected were dried at 40 °C. Dried and powdered leaves of each species were extracted using an accelerated solvent extractor (ASE 300 Thermo Scientific Dionex, Sunnyvale, CA, USA). It was operated at 40 °C and 100 bar, in 5 cycles with a purge time of 120 s and a pause time of 5 min. Four successive extractions were carried out with ethyl acetate (analytical grade, Carlo Erba, Val de Reuil, France) to exhaust the plant material. The extracts were pooled and evaporated under reduced pressure and kept at 4 °C until analysis.

### 3.4. Molecular Networking Parameters

Molecular networking permits the visualization of related molecules based on the comparison of their MS/MS data [12,26]. The network was created from isohexane and methanolic fractions of AcOEt extract of *Psiadia dentata* leaves using the online workflow at the GNPS web platform (http://gnps.ucsd.edu; accessed on 22 May 2020). LC-MS/MS data were acquired from UHPLC-HRMS-ESI-QTOF in positive ionization mode (20 eV and 40 eV). Analytical HPLC was performed using a Luna C_18_ column (150 mm × 2.1 mm i.d., 1.6 μm) (Phenomenex, Torrance, CA, USA) with 0.5 mL·min^−1^ gradient elution with 10% ACN-H_2_O (+0.1% formic acid) to 100% ACN-H_2_O (+0.1% formic acid) over 15 min. The MS/MS data were converted to the .mzXML format using MS-Convert 3.0.24 software, part of the ProteoWizard package [82] and then uploaded to the GNPS web platform for MN. The data were clustered with a parent mass tolerance of 0.02 Da and an MS/MS fragment ion tolerance of 0.02 Da to create consensus spectra. A network was then created where edges were filtered to have a cosine score above 0.62 and more than six matched peaks. The following parameters were also specified: network TopK: 10, minimum cluster size: 2, and maximum connected component size: 100. The spectral library matching was performed with cosine score above 0.7 and six matched fragment ions. The generated molecular network was visualized using Cytoscape 3.8.2 software [83].

### 3.5. Metabolomic Analysis

#### 3.5.1. ^1^H NMR Spectroscopy of Plant Extracts for Metabolomics

^1^H NMR metabolomic analysis was adapted from previously described protocols [84]. For each sample, 15 ± 0.2 mg of ethyl acetate crude extract was transferred to a 2 mL Eppendorf tube. A volume of 1.5 mL of deuterated chloroform (CDCl_3_, D 99.9 atom %, Eurisotop, Saint-Aubin, France) containing 0.03% TMS (tetramethylsilane) was added to each sample. The mixture was vortexed at room temperature for 1 min. An aliquot of 0.6 mL was transferred into 5 mm NMR tubes for NMR analysis. ^1^H NMR spectra were recorded in CDCl_3_ and consisted of 64 scans. CDCl_3_ was used as the internal lock. The acquisition consisted of 64 scans and the spectra had a spectral width of 12 ppm (7211 Hz). Each ^1^H-NMR spectrum was recorded with the following parameters: a standard one-pulse sequence with 30° flip angle and a relaxation delay (RD) = 1.0 s. Free induction decays (FIDs) were Fourier-transformed with line broadening (LB) = 0.3 Hz. The resulting spectra were manually phased, baseline-corrected, and calibrated to TMS at 0.00 ppm using MestReNova 10.0 (Mestrelab Research S.L.). Spectral intensities were scaled to total intensity and bucketed (bin width of 0.02 ppm) from the spectral region of 0.32–10.02 ppm. The region between δ_H_ 7.05 and 7.49 ppm was removed before further analysis due to the residual solvent signal.

#### 3.5.2. Multivariate Data Analysis

Chemometric analysis was performed with SIMCA version 12.0 (Umetrics, Umea, Sweden) in the form of a supervised orthogonal partial least squares discriminant analysis. The scaling was based on the Pareto method. Rx^2^ and Q^2^ values described the quality of the model. Rx^2^ indicated goodness of fit and is defined as the proportion of the variance in the data observed in the model. Q^2^ is defined as the proportion of variance in the data that was predictable by the model.

### 3.6. Phytochemical Investigation and Compound Isolation

Eight grams of extract was partitioned with isohexane and MeOH, yielding 4.53 and 3.36 g, respectively of isohexane and methanolic fractions. The hydrocarbon fraction (4.25 g) was separated by MPLC on normal phase (Buchi, Singapore, 230 × 15 mm i.d.) 15 mL. min^−1^ using a gradient of isohexane and EtOAc of increasing polarity. The eluates were monitored by TLC. Fractions with the same chromatographic profile were combined. Altogether, 8 fractions, F1–F8, were obtained. HPLC analysis of these 8 fractions allowed the selection of 4 fractions, F2, F4, F7 and F8, containing the majority of prevalent compounds for further fractionation.

Fraction F2 (50 mg) was subjected to semipreparative HPLC (Gemini C_18_ prep column, 5 µm, 250 × 10 mm i.d.), 4.5 mL·min^−1^ gradient elution with 60% ACN−H_2_O (+0.1% formic acid) to 75% ACN−H_2_O (+0.1% formic acid) over 35 min (UV 210 nm) to give pure compounds **1** (0.3 mg), **2** (0.8 mg), **18** (0.2 mg) in mixture with another flavonoid, **26** and **27** (mixture 50:50, 0.6 mg) and **28** (0.3 mg).

Purification of fraction F4 (45 mg) was performed by semipreparative HPLC (Gemini C_18_ prep column, 5 µm, 250 × 10 mm i.d.), 4.5 mL·min^−1^ gradient elution with 50% ACN−H_2_O (+0.1% formic acid) to 85% ACN−H_2_O (+0.1% formic acid) over 35 min (UV 210 nm) to give compounds **1** (mixture 50:50 with compound **36**, 1.3 mg), **3** (0.8 mg), **4** and **37** (mixture 50:50, 0.9 mg), and **29** (mixture 43:39:13:5 with **37**, 0.3 mg and another non-identified derived diterpene *ent*-labdane and a flavonoid).

Fraction F7 (70 mg) was purified by semipreparative HPLC (Gemini C_18_ prep column, 5 µm, 250 × 10 mm i.d.), 4.5 mL·min^−1^ isocratic elution with 50% ACN−H_2_O (+0.1% formic acid) over 15 min and elution gradient to 95% ACN−H_2_O (+0.1% formic acid) over 15 min and maintained at 95% ACN−H_2_O (+0.1% formic acid) over 15 min (UV 210 nm). Purification yielded compounds **5** (0.5 mg), **11** (0.6 mg), **30** (1.3 mg), **31** and **32** (mixture 50:50, 3.8 mg), **38** and **39** (mixture 67:33, 1.1 mg), and the compounds **26** and **27** (mixture 50:50, 2.1 mg), which had already been isolated from fraction F2.

Fraction F8 was first submitted to MPLC on normal phase (Buchi, 230 × 15 mm i.d.), 15 mL·min^−1^ using a gradient of isohexane and EtOAc of increasing polarity. Seven sub-fractions SF1–SF7 (4.4 mg to 165 mg) were obtained based on TLC profiles. After HPLC analysis, SF4 and SF5 (83.5 g) were grouped and re-purified by MPLC on normale phase (Buchi, 100 × 15 mm i.d.) 10 mL·min^−1^ using a gradient of isohexane and EtOAc of increasing polarity to yield ten subfractions. Separation of subfraction SF4 + 5.6 (6.7 mg) was performed by semipreparative HPLC (Gemini C_18_ prep column, 5 µm, 250 × 10 mm i.d.), 4.5 mL·min^−1^ isocratic elution with 50% ACN−H_2_O (+0.1% formic acid) over 38 min, then a gradient to 65% ACN−H_2_O (+0.1% formic acid) over 15 min and maintained at 65% ACN−H_2_O (+0.1% formic acid) over 15 min (UV 210 nm) to give pure compound **33** (0.4 mg). Similarly, SF4 + 5.8 (11.1 mg) was purified by semi-preparative HPLC (50% ACN−H_2_O (+0.1% formic acid) over 25 min, elution gradient to 100% ACN−H_2_O (+0.1% formic acid) over 1 min and maintained at 100% ACN−H_2_O (+0.1% formic acid) over 10 min (UV 210 nm), giving pure compound **34** (0.5 mg).

The MeOH-soluble fraction (60 mg) was subjected to semipreparative HPLC (Gemini C_18_ prep column, 5 µm, 250 × 10 mm i.d.), 4.5 mL·min^−1^ gradient elution with 90% ACN−H_2_O (+0.1% formic acid) to 98% ACN−H_2_O (+0.1% formic acid) over 15 min, 98% ACN−H_2_O (+0.1% formic acid) to 100% ACN−H_2_O (+0.1% formic acid) over 15 min and isocratic elution with 100% ACN−H_2_O (+0.1% formic acid) over 10 min to produce compounds **5** (0.8 mg), **6** (1.1 mg), **7** (5.0 mg), **8** (2.3 mg), **18** (0.6 mg) and compounds **19** and **35** (mixture 50:50, 2.9 mg).

### 3.7. NMR Sample Preparation and Spectroscopic Analysis

NMR analyses were conducted on compounds obtained following chromatographic isolation. Individual compounds were analyzed in their pure form when available, while other samples were characterized as mixtures of 2 or 3 compounds, as described in the purification section. Each sample was dissolved in an appropriate deuterated solvent according to its solubility properties, using either chloroform-d (CDCl_3_, 99.96% D, Eurisotop, ref. D029T) or methanol-d_4_ (CD_3_OD, 99.95% D, Eurisotop, ref. D048T). Compounds isolated from the isohexane fraction (**1**–**4**, **11**, **26**–**34**, and **36**–**39**) were dissolved in CDCl_3_, whereas those obtained from the methanolic fraction (**5**–**8**, **18**, **19**, and **35**) were dissolved in CD_3_OD. Approximately 0.2–3.8 mg of isolated compound was dissolved in 85 µL of the selected deuterated solvent. The solution was vortexed to ensure complete dissolution and then transferred into 2.0 mm NMR tubes (final volume = 85 µL). Prior to analysis, samples were centrifuged to remove any insoluble particles. Tubes were mounted on a spinner turbine and directly introduced into the spectrometer for data acquisition. The deuterated solvent used for each compound is specified in the corresponding NMR spectrum provided in the Supplementary Materials.

The 1D (^1^H and ^13^C) and 2D (COSY, HSQC, HMBC and NOESY) NMR spectra were acquired at 300 K with the high-field NMR spectrometer previously described, operating at 600.13 MHz for ^1^H and 150 MHz for ^13^C. NMR spectra were recorded using standard Bruker pulse sequences and processed with TopSpin 4.1.1 software (Bruker Biospin, Billerica, MA, USA). Chemical shifts (δ) are expressed in parts per million (ppm), using the residual CDCl_3_ signal (δ_H_ 7.26; δ_C_ 77.16) or CD_3_OD signal (δ_H_ 3.31; δ_C_ 49.0) as internal references for ^1^H and ^13^C NMR experiments. Coupling constants (*J*) are reported in Hertz (Hz).

### 3.8. Compound Characterization

**3α-angeloyloxy-19-hydroxy-*ent*-labda-8(17),13-dien-15,16-olide** (**26** and **27**): yellow powders; [α]_D_^25^: −145 (*c* 0.02, CHCl_3_); λ_max_ 225 nm; IR (ATR) *υ* 2925, 2853, 1757, 1646, 1260 cm^−1^; ^1^H and ^13^C NMR data, see Table 2 and Table 4; HREIMS [M + H]^+^ *m*/*z* 433.2587 (calcd. for C_25_H_37_O_6_ [M + H]^+^, *m*/*z* 433.2585).

**3α-angeloyloxy-16-hydroxy-*ent*-labda-8(17)-en-13-oic acid** (**28**): white paste; [α]_D_^25^: −50 (*c* 0.02, MeOH); λ_max_ 199 nm; IR (ATR) *υ* 3305, 2963, 2923, 2853, 1715, 1261 cm^−1^; ^1^H and ^13^C NMR data, Table 2 and Table 4; HREIMS [M + H]^+^ *m*/*z* 379.2486 (calcd. for C_22_H_35_O_5_^+^, *m*/*z* 379.2400).

**3α-angeloyloxy-19-hydroxy-*ent*-labda-8(17),13*E*-dien-15-al** (**29**): white oil; [α]_D_^25^: −30 (*c* 0.04, MeOH); λ_max_ 227 nm, IR (ATR) *υ* 2924, 2854, 1733, 1673, 1260, 1022, 798 cm^−1^; ^1^H and ^13^C NMR data, see Table 2 and Table 4; HREIMS [M + NH_4_]^+^ *m*/*z* 420.3108 (calcd. for C_25_H_42_O_4_N^+^, *m*/*z* 420.3109).

**3α-angeoyloxy-19-hydroxy-15,16-dimethoxy-*ent*-labda-8(17)-en-furan-13,14-diol** (**30**): white paste; [α]_D_^25^: −51 (*c* 0.04, MeOH); λ_max_ 197 nm; IR (ATR) *υ* 2953, 2929, 2854, 1692, 1644, 1260, 1022 cm^−1^; ^1^H and ^13^C NMR data, Table 2 and Table 4; HREIMS [M − H]^−^ *m*/*z* 495.2049 (calcd. for C_27_H_43_O_8_^−^, *m*/*z* 495.2963).

**3*α*-angeloyloxy-19,15(*R*/*S*)-dihydroxy-*ent*-andrograpanin** (**31** and **32**): white paste; [α]_D_^25^: −145 (*c* 0.02, MeOH); λ_max_ 197 nm; IR (ATR) *υ* 3384, 2936, 2855, 1761, 1709, 1643, 1603, 1440, 1386, 1237–1261, 1041 cm^−1^; ^1^H and ^13^C NMR data, see Table 3 and Table 4; HREIMS [M + H]^+^ *m*/*z* 433.2589 (calcd. for C_25_H_37_O_6_^+^, *m*/*z* 433.2585).

**3*α*-angeloyloxy-15,16-dihydroxy-*ent*-labda-8(17),13*E*-diene** (**33**): white crystals; [α]_D_^25^: −54 (*c* 0.04, MeOH); IR (ATR) *υ* 3354, 2961, 2926, 2855, 1713, 1603, 1456, 1384, 1261, 1027–1093 cm^−1^; ^1^H and ^13^C NMR data, see Table 3 and Table 4; HRESIMS [M + H]^+^ *m*/*z* 427.2815 (calcd. for C_25_H_41_O_4_^+^, *m*/*z* 427.2819).

**3*α*-angeloyloxy-15,16,19-trihydroxy-*ent*-labda-8(17),13*E*-diene** (**34**): white crystals; [α]_D_^25^: −135 (*c* 0.02, MeOH); λ_max_ 194 nm; IR (ATR) *υ* 3297, 2965, 2931, 2827, 1700, 1621, 1466, 1357, 1270, 1028–1115 cm^−1^; ^1^H and ^13^C NMR data, see Table 3 and Table 4; HRESIMS [M + Na]^+^ *m*/*z* 443.2764 (calcd. for C_25_H_40_O_5_Na^+^, *m*/*z* 443.2768).

**5-hydroxydihydroluvangetin** (**35**): pale yellow powders; [α]_D_^25^: −63 (*c* 0.04, MeOH); λ_max_ 211 nm; IR (ATR) *υ* 3293, 2968, 2933, 2857, 1700, 1620, 1503, 1467, 1377, 1181, 1029–1143, 804–927 cm^−1^; ^1^H and ^13^C NMR data, see Table 3 and Table 4; HRESIMS [M + H]^+^ *m*/*z* 277.1083 (calcd. for C_15_H_17_O_5_^+^, *m*/*z* 277.1071).

### 3.9. Biological Assays

#### 3.9.1. *In Vitro* Antiplasmodial Assay

The *P. falciparum* strains utilized and details of the assay protocols have been previously reported [69,85].

#### 3.9.2. *In Vitro* HRP Inhibition Assay

The HRP (horseradish peroxidase) anti-peroxidase activity was evaluated using chemiluminescence technique according to the method previously reported [86].

#### 3.9.3. *In Vitro* NO Inhibition Assay

It was determined in mouse immortalized macrophages (RAW 264.7 cell line, Sigma-Aldrich) as described previously [74,87].

#### 3.9.4. *In Vitro* Cytotoxic Assay

HepG2 human liver hepatocellular carcinoma and HT29 human colorectal adenocarcinoma cells, provided by ATCC HTB-38 were used to assess the toxicity of crude extracts, fractions and pure compounds. The protocol used was previously reported [88,89].

## 4. Conclusions

In this study, ^1^H NMR-based metabolomics was applied to investigate the biological potential of eleven *Psiadia* species in Reunion Island. The ^1^H NMR fingerprints revealed distinct chemical profiles among all studied species of the genus *Psiadia*. All *Psiadia* ethyl acetate crude extracts were evaluated for antiplasmodial and anti-inflammatory activities with three species (*P. amygdalina*, *P. anchusifolia*, and *P. dentata*) showing particularly notable activity. Among the most active species, *P. dentata*, a particularly promising candidate, was selected for subsequent studies because of its notable antiplasmodial and anti-inflammatory activities. Correlation of the ^1^H NMR data and the IC_50_ values using PLS-DA modelling highlighted three distinct groups based on activity classes. This multivariate analysis identified the flavonoids ermanin (**1**) and isokaempferide (**7**), as well as the coumarin isoobtusitin (**18**) as key contributors to the antiplasmodial activity of *P. dentata*, while minor compounds present in trace amounts may also contribute.

The MN approach, applied to *P. dentata*, revealed 25 specialized metabolites (**1**–**25**) from diverse chemical families, including diterpenoids, flavonoids, coumarins, and alkaloids, highlighting the chemodiversity of this species.

Phytochemical investigation of *P. dentata* led to the isolation and structural identification of 25 metabolites. To our knowledge, compounds **26**–**35** are reported here for the first time, nine new diterpenes (**26**–**34**) and one new coumarin (**35**). Compounds **1**, **7**, **26** and **27** displayed moderate antiplasmodial activity against *Plasmodium falciparum* (IC_50_ = 7.25–13.46 μM). Five compounds, **7**, **26**, **27**, **31** and **32** (IC_50_ = 0.87–27.71 μM), showed inhibition of nitric oxide production, consistent with the traditional medicinal uses of this species. Only compound **1** showed cytotoxicity against HepG2 and HT29 cell lines (IC_50_ = 25.67 and 18.35 μM, respectively).

Overall, this study demonstrates that the combination of ^1^H NMR-based metabolomics, molecular networking and phytochemical investigation constitutes a powerful strategy for the identification of bioactive metabolites. The chemical composition of *P. dentata*, underscores its richness in specialized metabolites, representing a promising source of therapeutic agents.

## Figures and Tables

**Table 1:** Summary table of molecular network annotations for *Psiadia dentata* leaf extract.

Natural Product Family	Compound	*m*/*z*	Precursor Ion	Raw Formula	Structural Analogue
Flavonoids	**1**	315.091	[M + H]^+^	C_17_H_14_O_6_	ermanin
	**2**	328.79	[M + H]^+^	C_18_H_16_O_6_	kaempferol 3,7,4′-trimethylether
	**3**	347.081	[M + H]^+^	C_18_H_16_O_7_	patchypodol
	**4**	359.117	[M + H]^+^	C_19_H_18_O_7_	retusin
	**5**	345.131	[M + H]^+^	C_18_H_16_O_7_	penduletin
	**6**	317.070	[M + H]^+^	C_16_H_12_O_7_	quercetin 3-methylether
	**7**	301.048	[M + H]^+^	C_16_H_12_O_6_	isokaempferide
	**8**	331.086	[M + H]^+^	C_17_H_14_O_7_	quercetin 3,3′-dimethylether
	**9**	287.060	[M + H]^+^	C_15_H_10_O_6_	kaempferol
	**10**	361.097	[M + H]^+^	C_18_H_16_O_8_	chrysosplenol D
Diterpenoids	**11**	333.212	[M + H]^+^	C_20_H_28_O_4_	*ent*-labda-8(17),13-dien-15,16-olide-19-oic acid
	**12**	315.201	[M + H]^+^	C_19_H_32_O_2_	annosquamosine C
	**13**	317.216	[M + H]^+^	C_20_H_38_O_3_	7-hydroxycallitrisic acid
	**14**	333.047	[M + NH_4_]^+^	C_20_H_28_O_3_	methyl-*ent*-12-oxopimara-9(11),15-dien-19-oic acid
	**15**	303.237	[M + H]^+^	C_20_H_30_O_2_	6-deoxypsiadiol
	**16**	317.035	[M + H]^+^	C_20_H_28_O_3_	3-oxo-19-hydroxy-13-furyl-ent-labda-8(17)-ene
	**17**	351.222	[M + H]^+^	C_20_H_30_O_5_	andrographolide
Coumarins	**18**	277.113	[M + H]^+^	C_15_H_16_O_5_	isoobtusitin
	**19**	209.048	[M + H]^+^	C_10_H_8_O_5_	fraxetin
	**20**	293.107	[M + H]^+^	C_15_H_16_O_6_	7-(2′,3′-epoxy-3′-methylbutoxy)-8-hydroxy-6-methoxycoumarin
	**21**	311.117	[M + H]^+^	C_15_H_18_O_7_	7-(2′,3′-dihydroxy-3′-methylbutoxy)-8-hydroxy-6-methoxycoumarin
	**22**	179.038	[M + H]^+^	C_9_H_6_O_4_	esculetin
	**23**	247.101	[M + H]^+^	C_14_H_14_O_4_	prenyletin
Alkaloids	**24**	235.174	[M + Na]^+^	C_10_H_18_N_2_O_3_	desthiobiotin
	**25**	181.126	[M + H]^+^	C_12_H_21_N	rimantadine

**Table 5:** Area of collects and voucher number of each *Psiadia* species.

*Psiadia* Species	Collection Site	Voucher Number
***P. amygdalina***	Salazie	REU13109
	Maïdo	REU13115
	Volcan	REU13130
***P. anchusifolia***	Salazie	REU13111
	Maïdo	REU13117
	Volcan	REU13130
	Roche-Ecrite	REU13131
***P. argentea***	Salazie	REU13112
	Volcan	REU13143
	Roche-Ecrite	REU13135
***P. boivinii***	Salazie	REU13113
	Maïdo	REU13116
	Volcan	REU13142
	Roche-Ecrite	REU13133
***P. callocephala***	Volcan	REU13140
***P. dentata***	Maïdo	REU13119
	Colorado	REU13139
	Dos d’Ane	REU13128
	Montauban	REU13487
***P. insignis***	Maïdo	REU13484
	Volcan	REU13483
	Dos d’Ane	REU13125
***P. laurifolia***	Salazie	REU13114
	Maïdo	REU13118
	Volcan	REU13141
	Roche-Ecrite	REU13132
***P. melastomatoides***	Salazie	REU13110
	Roche-Ecrite	REU13134
***P. montana***	Dos d’Ane	REU13127
***P. retusa***	Cap Jaune	REU13129
	Cap Méchant	REU13486
	Piton Grande Anse	REU13485

## Data Availability

NMR raw data (^1^H, ^13^C, gCOSY, gHSQC, gHMBC and NOESY) of compounds **26**–**35** are made freely available at https://doi.org/10.5281/zenodo.18294328. Raw data from this analysis data were deposited in the MassIVE Public GNPS data set (http://massive.ucsd.edu; accessed on 27 May 2020): MSV000088099. The molecular networking job on GNPS can be found at https://gnps.ucsd.edu/ProteoSAFe/status.jsp?task=12c133ae14b240a8ae5e74b60068bd92; accessed on 27 May 2020.

## Supplementary Materials

The following supporting information can be downloaded at: https://www.mdpi.com/article/10.3390/molecules31060973/s1, including the contribution plot of the PLS model of *P. dentata* crude extracts against *Psiadia* sp. inactive and moderately active extracts for the antiplasmodial activity., ^1^H NMR spectrum of the active crude extract and moderately active extract of *P. dentata*, and UV spectra, IR, 1D/2D NMR, HRMS data of compounds **26**–**35**, and chromatograms of o biologically tested compounds.
