# Supplementary material for: Metabolomics, Molecular Networking and Phytochemical Investigation of Psiadia dentata (Cass.) DC., Endemic to Reunion Island: Discovery of Novel Bioactive Molecules
Source: Molecules. 2026 Mar 13;31(6):973. doi: 10.3390/molecules31060973 (PMC13029444; doi:10.3390/molecules31060973)
Supplement: Supplementary file 1 [file molecules-31-00973-s001.zip › molecules-4148344-supplementary.pdf]

Supplementary materials

Metabolomics, molecular networking and phytochemical investigation of *Psiadia dentata* (Cass.) DC., endemic to Reunion Island: discovery of novel bioactive molecules

Lantomalala Elsa Razafindrabenja [1,2], Keshika Mahadeo [1], Gaëtan Herbette [3,4], Lúcia Mamede [5], Michel Frederich [5], Carole Di Giorgio [6], Béatrice Baghdikian [7], Patricia Clerc [1], Hippolyte Kodja [8], Isabelle Grondin [1] and Anne Gauvin-Bialecki [1,*]

[1] Laboratoire de Chimie et de Biotechnologie des Produits Naturels (ChemBioPro), Université de La Réunion, 15 Avenue René Cassin, CS 92 003, 97 744 Saint-Denis Cedex 9, La Réunion, France; lantomalala.razafindrabenja@univ-reunion.fr; keshika.mahadeo@univ-reunion.fr; patricia.clerc@univ-reunion.fr; isabelle.grondin@univ-reunion.fr; anne.bialecki@univ-reunion.fr

[2] SPO, University of Montpellier, INRAE, Institut Agro, Montpellier, France;

[3] CNRS, Aix-Marseille Université, Centrale Méditerranée, FSCM, Spectropole, Campus de St Jérôme-Service 511, 13397 Marseille, France; gaetan.herbette@univ-amu.fr or gaetan.herbette@cnrs.fr

[4] Institut de Chimie des Substances Naturelles (ICSN), Université Paris-Saclay, CNRS, UPR 2301, 91198 Gif-sur-Yvette, France

[5] Pharmacognosy Laboratory, Department of Pharmacy, Centre Interfacultaire de Recherche sur le Médicament (CIRM), University of Liège, Campus du Sart-Tilman, Quartier Hôpital, Avenue Hippocrate, 15, B36, 4000 Liège, Belgium; lucia.c.mamede@outlook.pt; m.frederich@uliege.be

[6] IMBE, Aix Marseille Univ, Avignon Univ, CNRS 7263, IRD 237, 27 Bd Jean Moulin, Service of Environmental Mutagenesis, Faculty of Pharmacy, 13385 Marseille, France; carole.di-giorgio@univ-amu.fr

[7] IMBE, Aix Marseille Univ, Avignon Univ, CNRS 7263, IRD 237, 27 Bd Jean Moulin, Service of Pharmacognosy, Faculty of Pharmacy, 13385 Marseille, France; beatrice.baghdikian@univ-amu.fr

[8] Qualisud, University Montpellier, CIRAD, Institut Agro, Avignon Université, Univ de La Réunion, 34093 Montpellier, France; hippolyte.kodja@univ-reunion.fr

* Correspondence: anne.bialecki@univ-reunion.fr; Tel.: +262-(0)2-6293-8197

Table of contents

Figure S1. Contribution plot of the PLS model of *P. dentata* crude extracts against *Psiadia* sp. inactive and moderately active extracts for the antiplasmodial activity. 5
Figure S2. ^{1}H NMR spectrum ($CDCl_3$, 600 MHz) of the active crude extract and moderately active extract of *P. dentata* with ermanin (1), isokaempferide (7), and isoobtusitin (18) key assignments. 6
Figure S3. UV spectrum of **26** and **27**.......... 7
Figure S4. HRESI$^+$MS spectrum of **26** and **27**.......... 7
Figure S5. IR (FT-IR) spectrum of **26** and **27**. 8
Figure S6. ^{1}H NMR ($CDCl_3$, 600 MHz) spectrum of **26** and **27**.......... 9
Figure S7. ^{13}C NMR ($CDCl_3$, 150 MHz) spectrum of **26** and **27**. 10
Figure S8. ^{1}H-^{1}H COSY NMR ($CDCl_3$, 600 MHz,) spectrum of **26** and **27**. 11
Figure S9. ^{1}H-^{13}C HSQC NMR (600 MHz, $CDCl_3$) spectrum of **26** and **27**.......... 12
Figure S10. ^{1}H-^{13}C HMBC NMR (600 MHz, $CDCl_3$) spectrum of **26** and **27**.......... 13
Figure S11. ^{1}H-^{1}H NOESY NMR (600 MHz, $CDCl_3$) spectrum of **26** and **27.** 14
Figure S12. UV spectrum of **28**. 15
Figure S13. HRESI$^+$MS spectrum of **28**. 15
Figure S14. IR (FT-IR) spectrum of **28**.......... 16
Figure S15. ^{1}H NMR ($CDCl_3$, 600 MHz) spectrum of **28**. 17
Figure S16. ^{1}H-^{1}H COSY NMR (600 MHz, $CDCl_3$) spectrum of **28**.......... 18
Figure S17. ^{1}H-^{13}C HSQC NMR (600 MHz, $CDCl_3$) spectrum of **28**. 19
Figure S18. ^{1}H-^{13}C HMBC NMR (600 MHz, $CDCl_3$) spectrum of **28**. 20
Figure S19. ^{1}H-^{1}C NOESY NMR (600 MHz, $CDCl_3$) spectrum of **28**. 21
Figure S20. UV spectrum of **29**. 22
Figure S21. HRESI$^+$MS spectrum of **29**. 22
Figure S22. IR (FT-IR) spectrum of **29**.......... 23
Figure S23. ^{1}H NMR ($CDCl_3$, 600 MHz) spectrum of **29**. 24
Figure S24. ^{1}H-^{1}H COSY NMR (600 MHz, $CDCl_3$) spectrum of **29**.......... 25
Figure S25. ^{1}H-^{13}C HSQC NMR (600 MHz, $CDCl_3$) spectrum of **29**. 26
Figure S26. ^{1}H-^{13}C HMBC NMR (600 MHz, $CDCl_3$) spectrum of **29**. 27
Figure S27. ^{1}H-^{1}C NOESY NMR (600 MHz, $CDCl_3$) spectrum of **29**. 28
Figure S28. UV spectrum of **30**. 29
Figure S29. HRESI$^-$MS spectrum of **30**.......... 29
Figure S30. IR (FT-IR) spectrum of **30**.......... 30
Figure S31. ^{1}H NMR ($CDCl_3$, 600 MHz) spectrum of **30**. 31
Figure S32. ^{13}C NMR ($CDCl_3$, 150 MHz) spectrum of **30**.......... 32
Figure S33. ^{1}H-^{1}H COSY NMR (600 MHz, $CDCl_3$) spectrum of **30**.......... 33
Figure S34. ^{1}H-^{13}C HSQC NMR (600 MHz, $CDCl_3$) spectrum of **30**. 34

Figure S35. ^{1}H-^{13}C HMBC NMR (600 MHz, $CDCl_3$) spectrum of **30**. 35
Figure S36. ^{1}H-^{1}H NOESY NMR (600 MHz, $CDCl_3$) spectrum of **30**. 36
Figure S37. UV spectrum of **31** and **32**. 37
Figure S38. HRESI^{+}MS spectrum of **31** and **32**. 37
Figure S39. IR (FT-IR) spectrum of **31** and **32**. 38
Figure S40. ^{1}H NMR ($CDCl_3$, 600 MHz) spectrum of **31** and **32**. 39
Figure S41. ^{13}C NMR ($CDCl_3$, 150 MHz) spectrum of **31** and **32**. 40
Figure S42. ^{1}H-^{1}H COSY NMR (600 MHz, $CDCl_3$) spectrum of **31** and **32**. 41
Figure S43. ^{1}H-^{13}C HSQC NMR (600 MHz, $CDCl_3$) spectrum of **31** and **32**. 42
Figure S44. ^{1}H-^{13}C HMBC NMR (600 MHz, $CDCl_3$) spectrum of **31** and **32**. 43
Figure S45. ^{1}H-^{1}H NOESY NMR (600 MHz, $CDCl_3$) spectrum of **31** and **32**. 44
Figure S46. UV spectrum of **33**. 45
Figure S47. HRESI^{+}MS spectrum of **33**. 45
Figure S48. IR (FT-IR) spectrum of **33**. 46
Figure S49. ^{1}H NMR ($CDCl_3$, 600 MHz) spectrum of **33**. 47
Figure S50. ^{1}H-^{1}H COSY NMR (600 MHz, $CDCl_3$) spectrum of **33**. 48
Figure S51. ^{1}H-^{13}C HSQC NMR (600 MHz, $CDCl_3$) spectrum of **33**. 49
Figure S52. ^{1}H-^{13}C HMBC NMR (600 MHz, $CDCl_3$) spectrum of **33**. 50
Figure S53. UV spectrum of **34**. 51
Figure S54. HRESI^{+}MS spectrum of **34**. 51
Figure S55. IR (FT-IR) spectrum of **34**. 52
Figure S56. ^{1}H NMR ($CDCl_3$, 600 MHz) spectrum of **34**. 53
Figure S57. ^{1}H-^{1}H COSY NMR (600 MHz, $CDCl_3$) spectrum of **34**. 54
Figure S58. ^{1}H-^{13}C HSQC NMR (600 MHz, $CDCl_3$) spectrum of **34**. 55
Figure S59. ^{1}H-^{13}C HMBC NMR (600 MHz, $CDCl_3$) spectrum of **34**. 56
Figure S60. UV spectrum of **35**. 57
Figure S61. HRESI^{+}MS spectrum of **35**. 57
Figure S62. IR (FT-IR) spectrum of **35**. 58
Figure S63. ^{1}H NMR ($CDCl_3$, 600 MHz) spectrum of **35**. 59
Figure S64. ^{1}H-^{13}C HSQC NMR (600 MHz, $CDCl_3$) spectrum of **35**. 60
Figure S65. ^{1}H-^{13}C HMBC NMR (600 MHz, $CDCl_3$) spectrum of **35**. 61
Figure S66. ^{1}H-^{1}H NOESY NMR (600 MHz, $CDCl_3$) spectrum of **35**. 62
Figure S67. HPLC-CAD chromatogram of compound **1**. 63
Figure S68. HPLC-CAD chromatogram of compound **2**. 64
Figure S69. HPLC-CAD chromatogram of compound **3**. 65
Figure S70. HPLC-CAD chromatogram of compound **5**. 66
Figure S71. HPLC-CAD chromatogram of compound **6**. 67
Figure S72. HPLC-CAD chromatogram of compound **7**. 68

Figure S73. HPLC-CAD chromatogram of compound **8**. 69
Figure S74. HPLC-CAD chromatogram of compound **11**. 70
Figure S75. HPLC-CAD chromatogram of compound **18**. 71
Figure S76. HPLC-CAD chromatogram of compound **28**. 72
Figure S77. HPLC-CAD chromatogram of compound **30**. 73
Figure S78. HPLC-CAD chromatogram of compound **33**. 74
Figure S79. HPLC-CAD chromatogram of compound **34**. 75
Figure S80. HPLC-CAD chromatogram of compounds **4** and **37**. 76
Figure S81. HPLC-CAD chromatogram of compounds **19** and **34**. 77
Figure S82. HPLC-CAD chromatogram of compounds **26** and **27**. 78
Figure S83. HPLC-CAD chromatogram of compounds **31** and **32**. 79
Figure S84. HPLC-CAD chromatogram of compounds **1** and **36**. 80
Figure S85. HPLC-CAD chromatogram of compounds **4** and **37**. 81
Figure S86. HPLC-CAD chromatogram of compounds **29** and **37**. 82
Figure S87. HPLC-CAD chromatogram of compounds **38** and **39**. 83

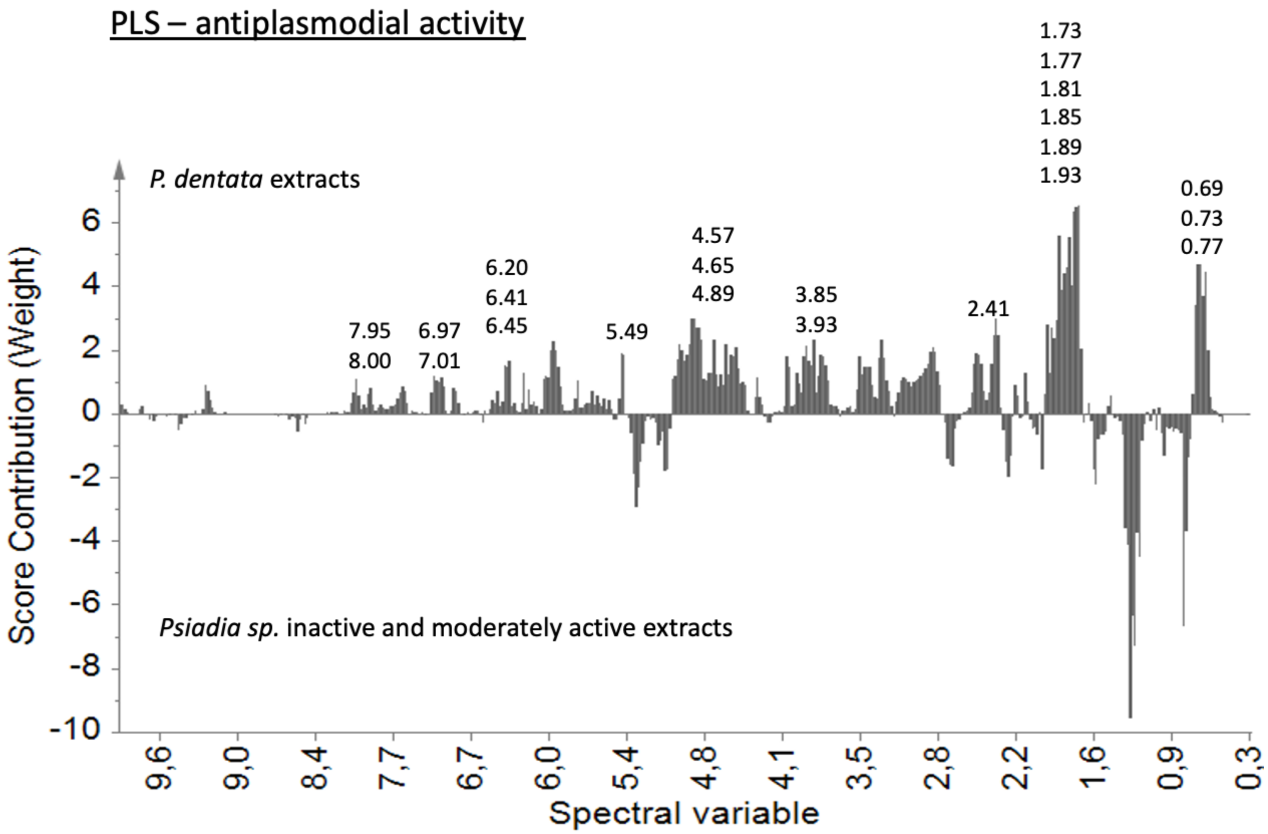

Figure S1. Contribution plot of the PLS model of *P. dentata* crude extracts against *Psiadia* sp. inactive and moderately active extracts for the antiplasmodial activity.

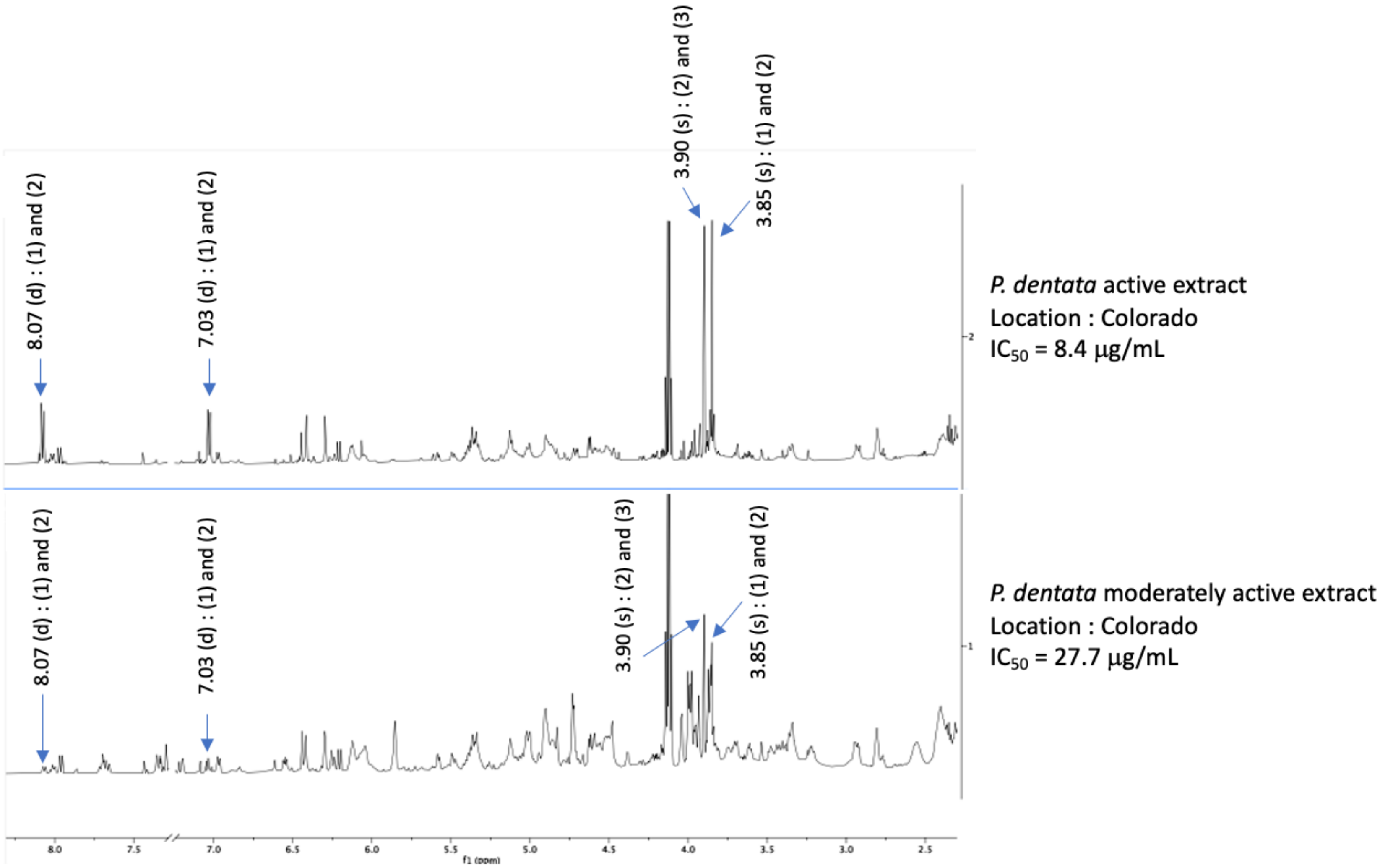

Figure S2. ^{1}H NMR spectrum ($CDCl_3$, 600 MHz) of the active crude extract and moderately active extract of *P. dentata* with ermanin (1), isokaempferide (7), and isoobtusitin (18) key assignments.

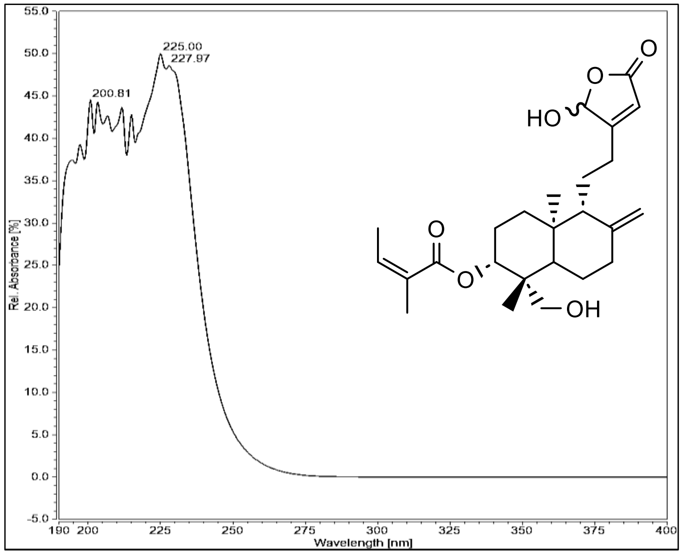

Figure S3. UV spectrum of **26** and **27**.

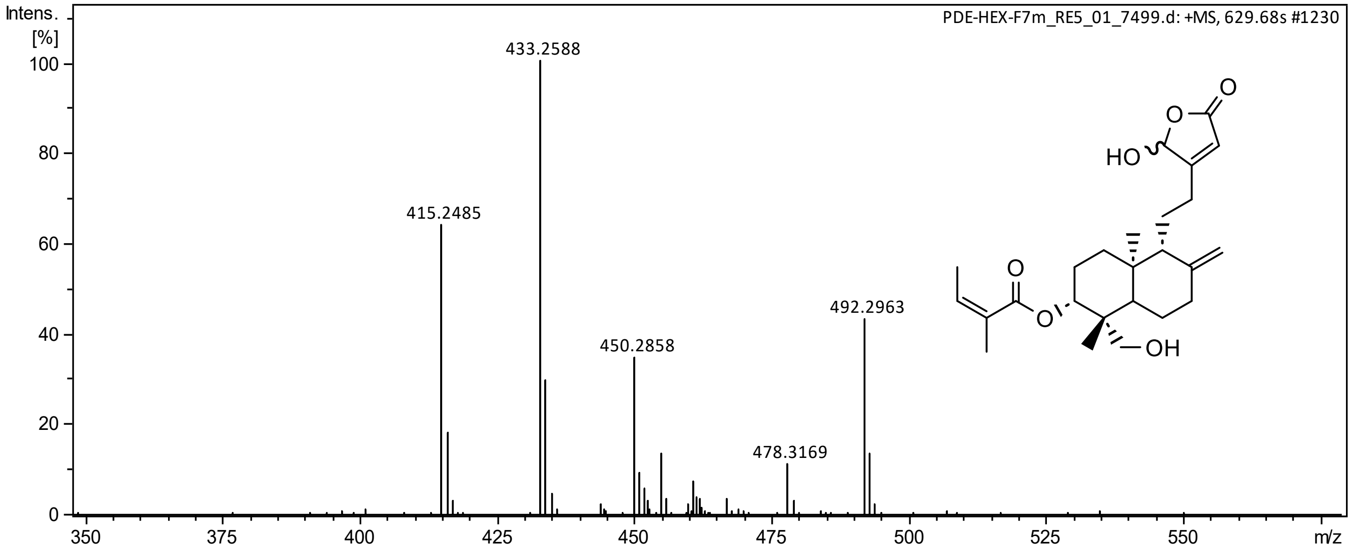

Figure S4. HRESI+MS spectrum of **26** and **27**.

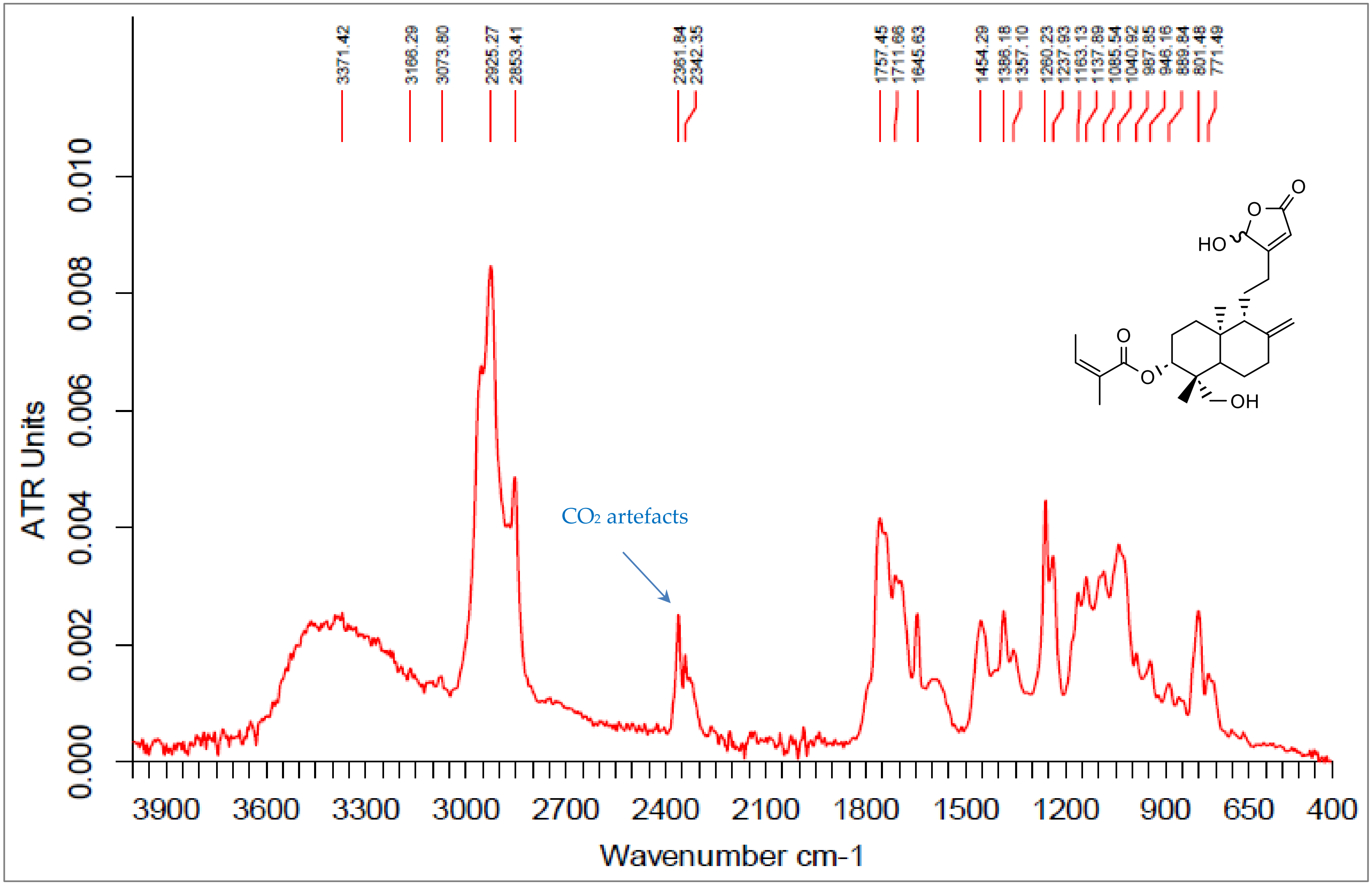

Figure S5. IR (FT-IR) spectrum of **26** and **27**.

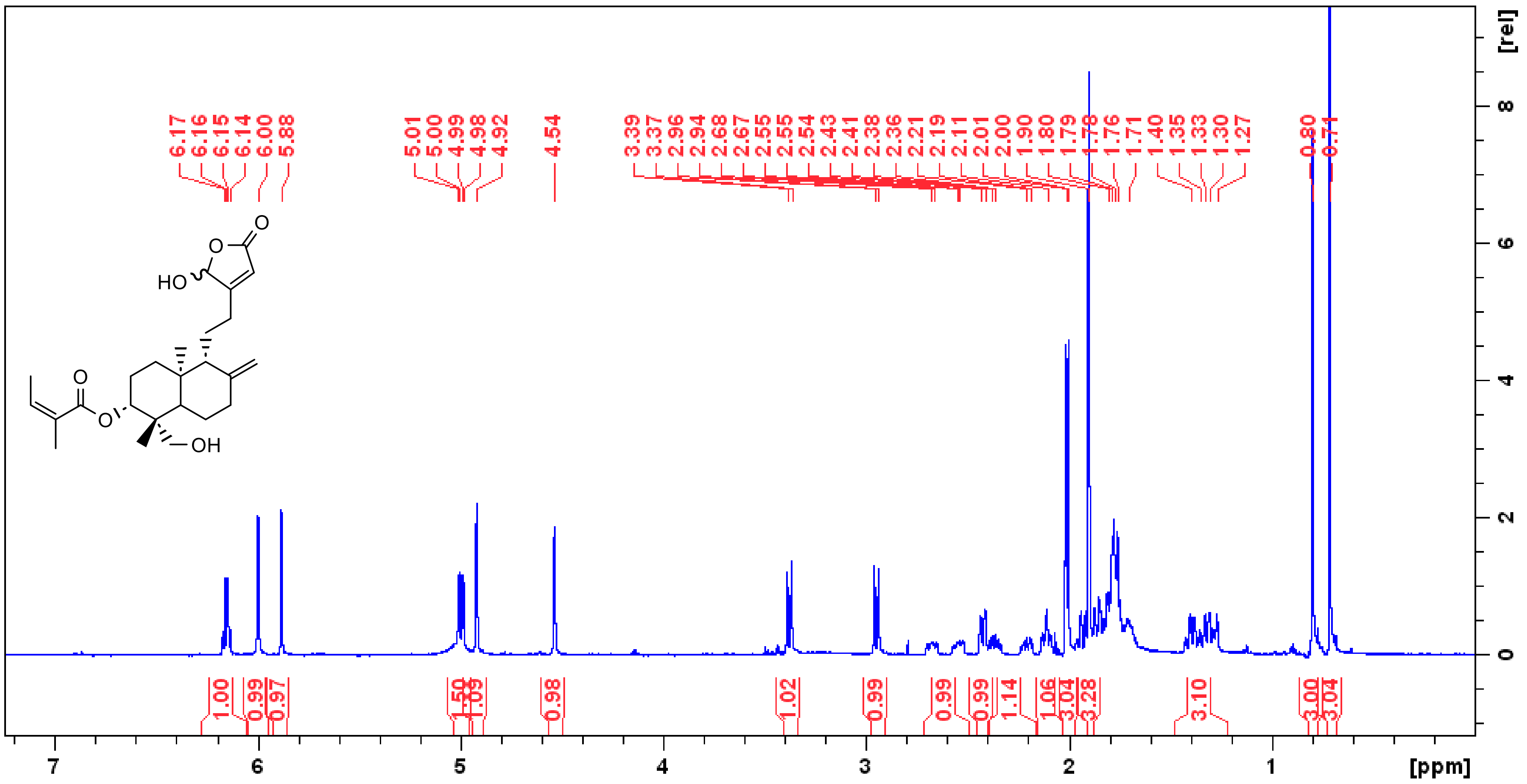

Figure S6. ^{1}H NMR ($CDCl_3$, 600 MHz) spectrum of **26** and **27**.

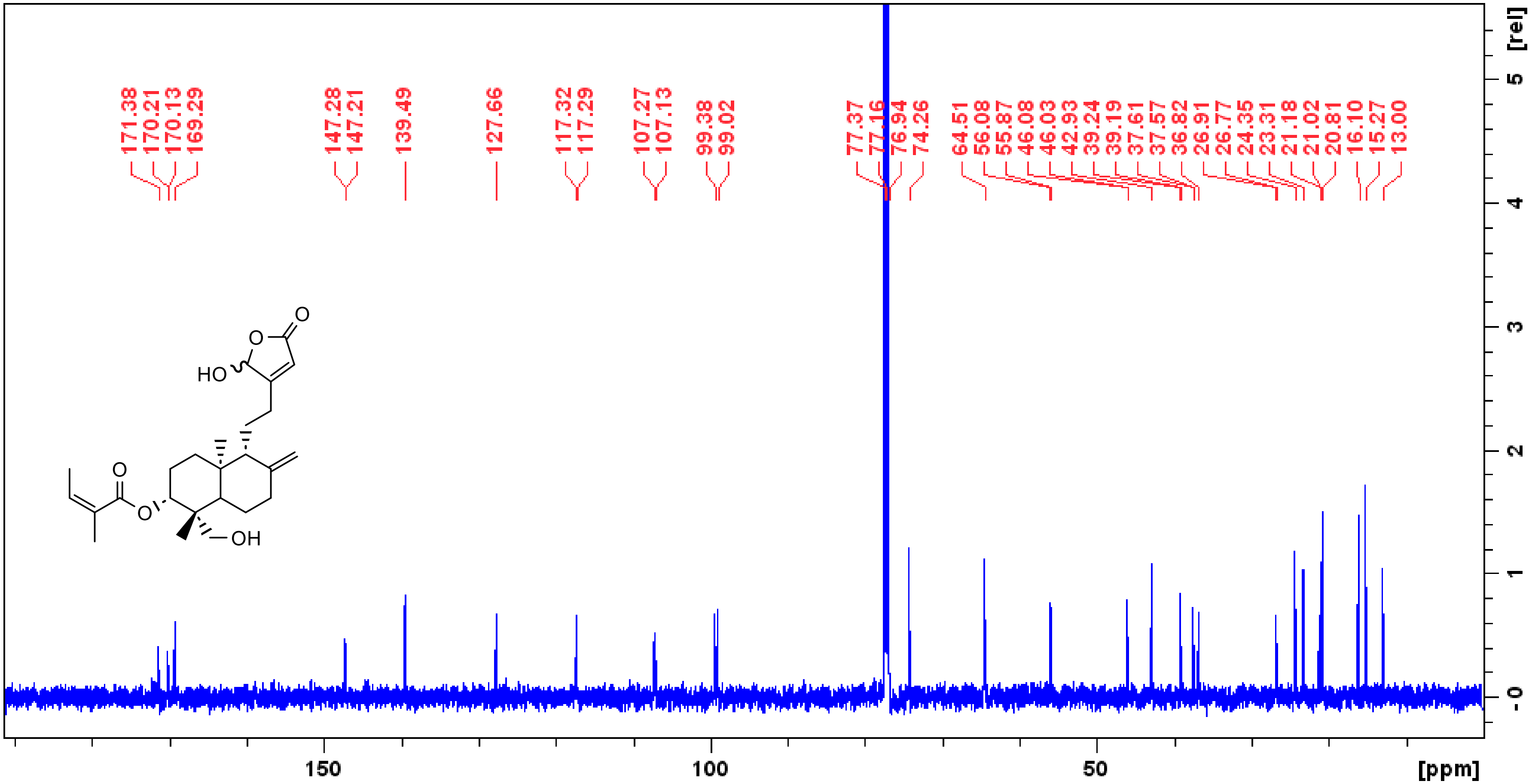

Figure S7. ^{13}C NMR ($CDCl_3$, 150 MHz) spectrum of **26** and **27**.

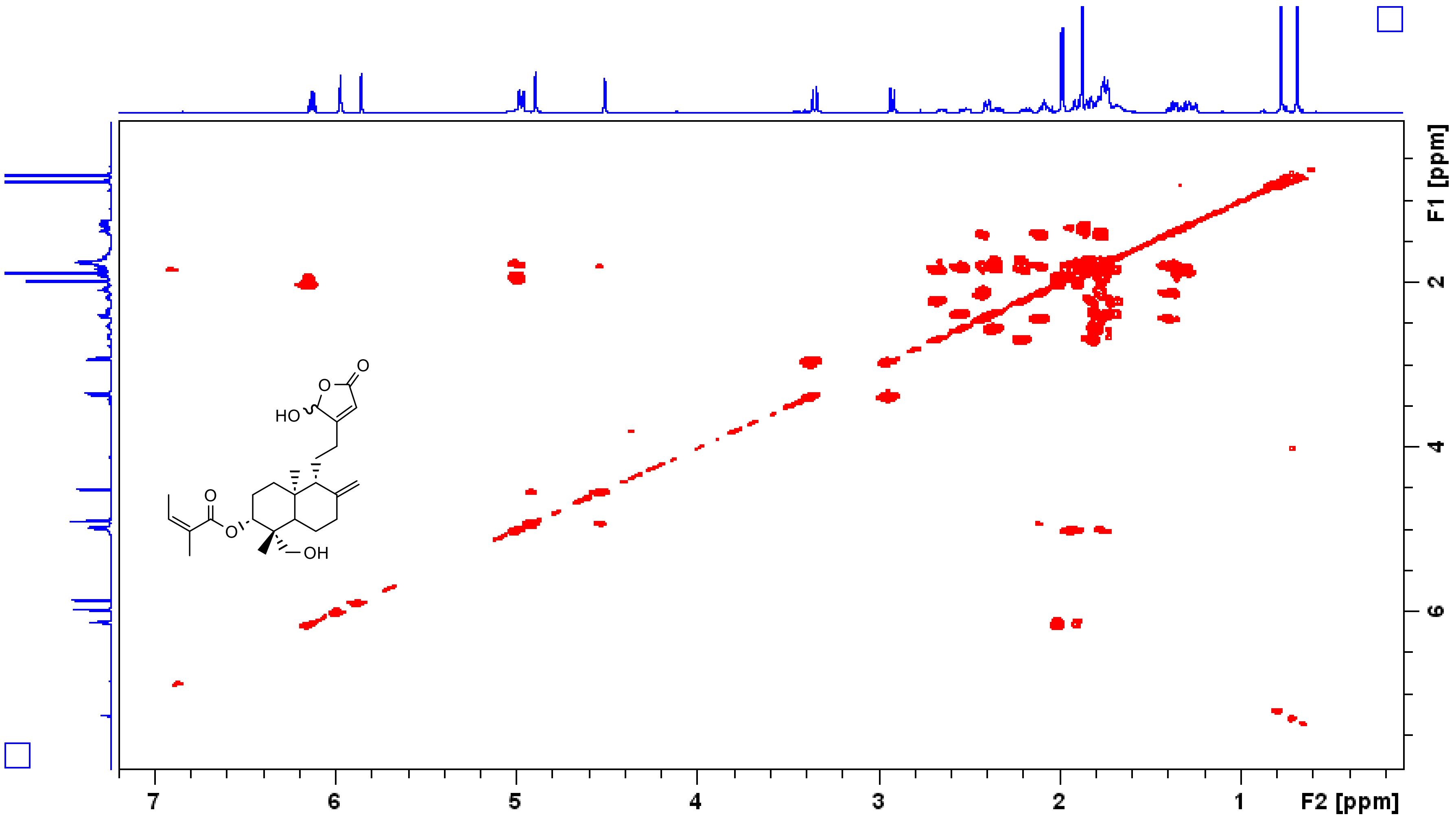

Figure S8. ^{1}H-^{1}H COSY NMR ($CDCl_3$, 600 MHz,) spectrum of **26** and **27**.

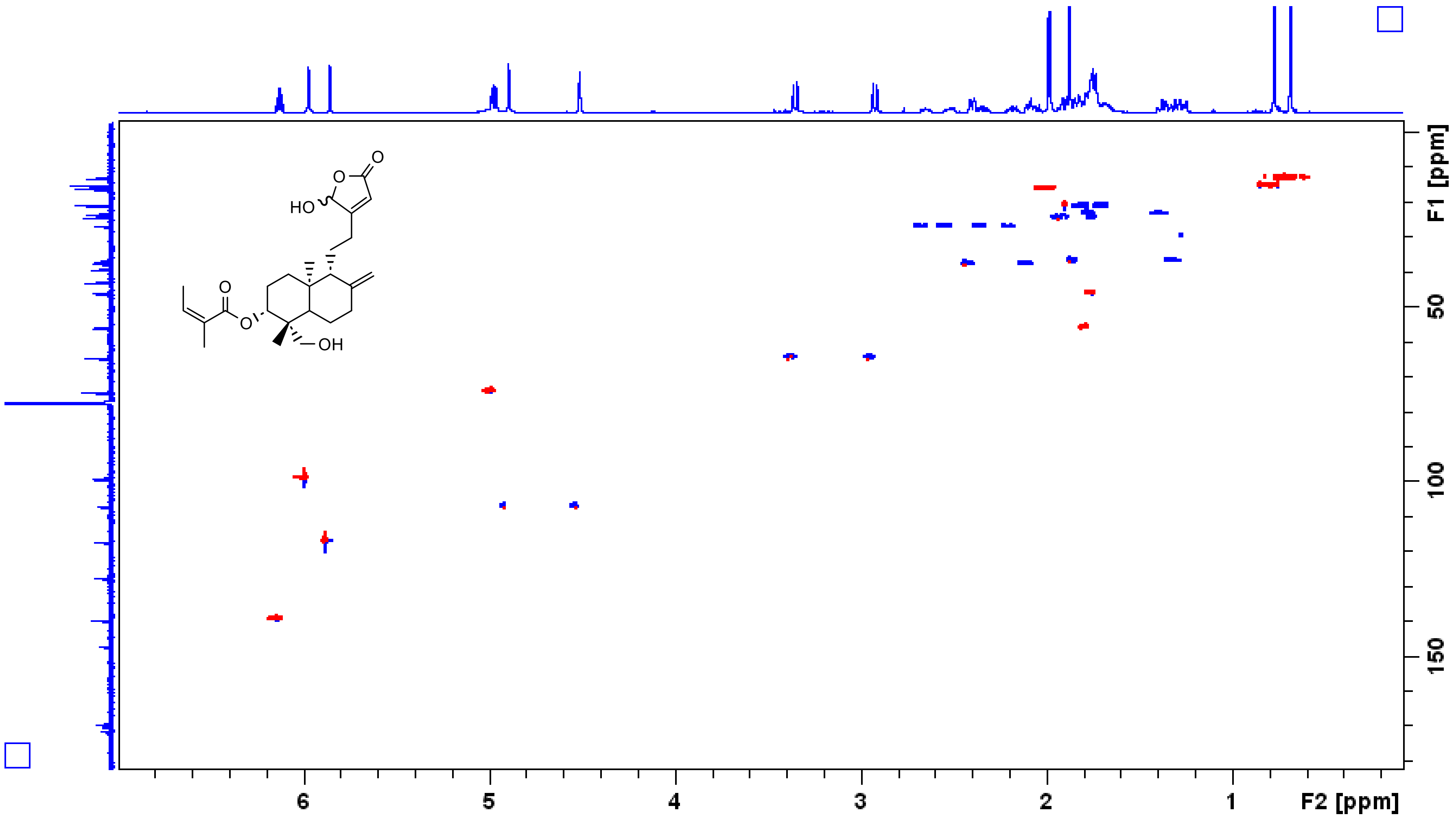

Figure S9. ^{1}H-^{13}C HSQC NMR (600 MHz, $CDCl_3$) spectrum of **26** and **27**.

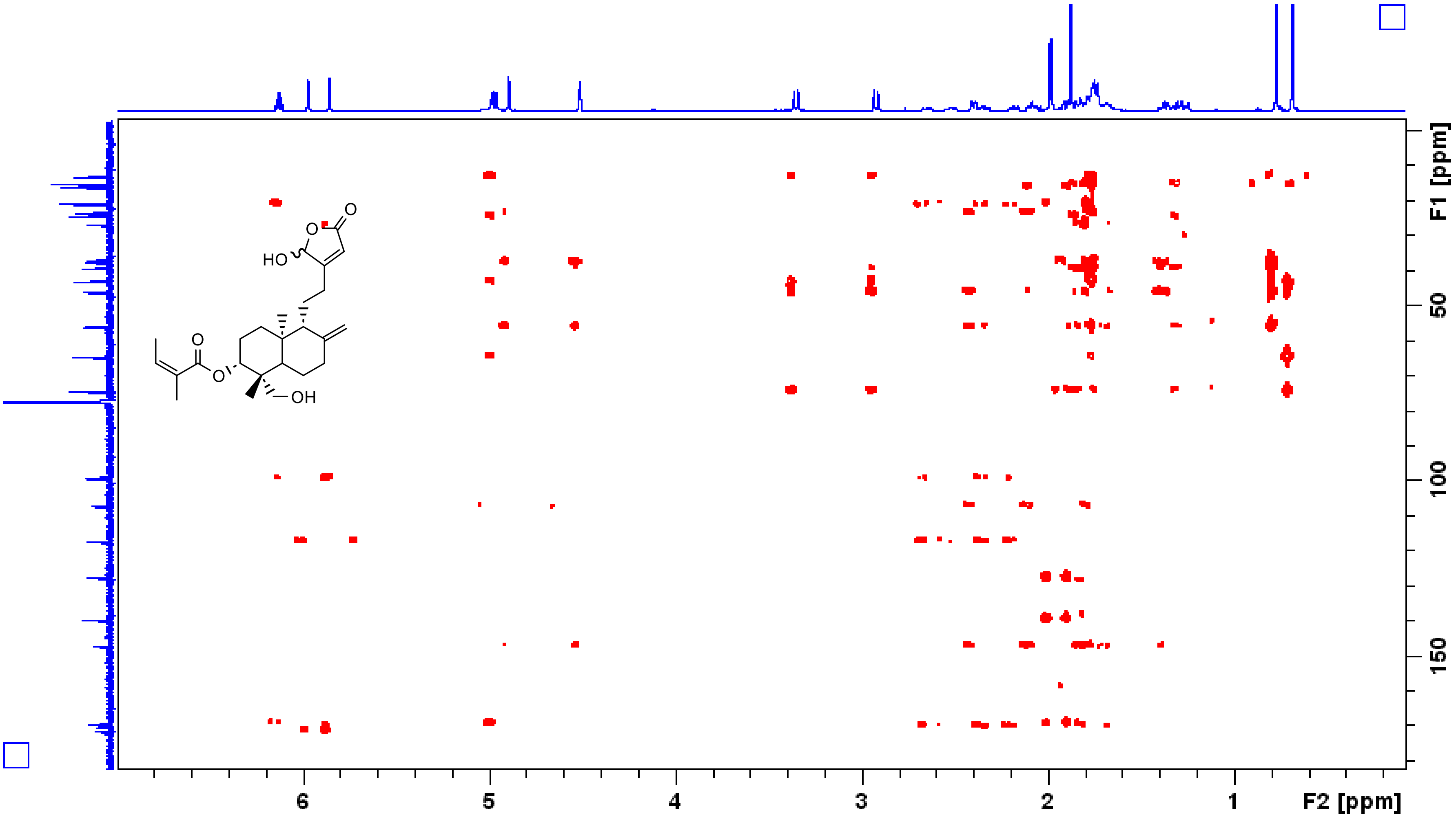

Figure S10. ^{1}H-^{13}C HMBC NMR (600 MHz, $CDCl_3$) spectrum of **26** and **27**.

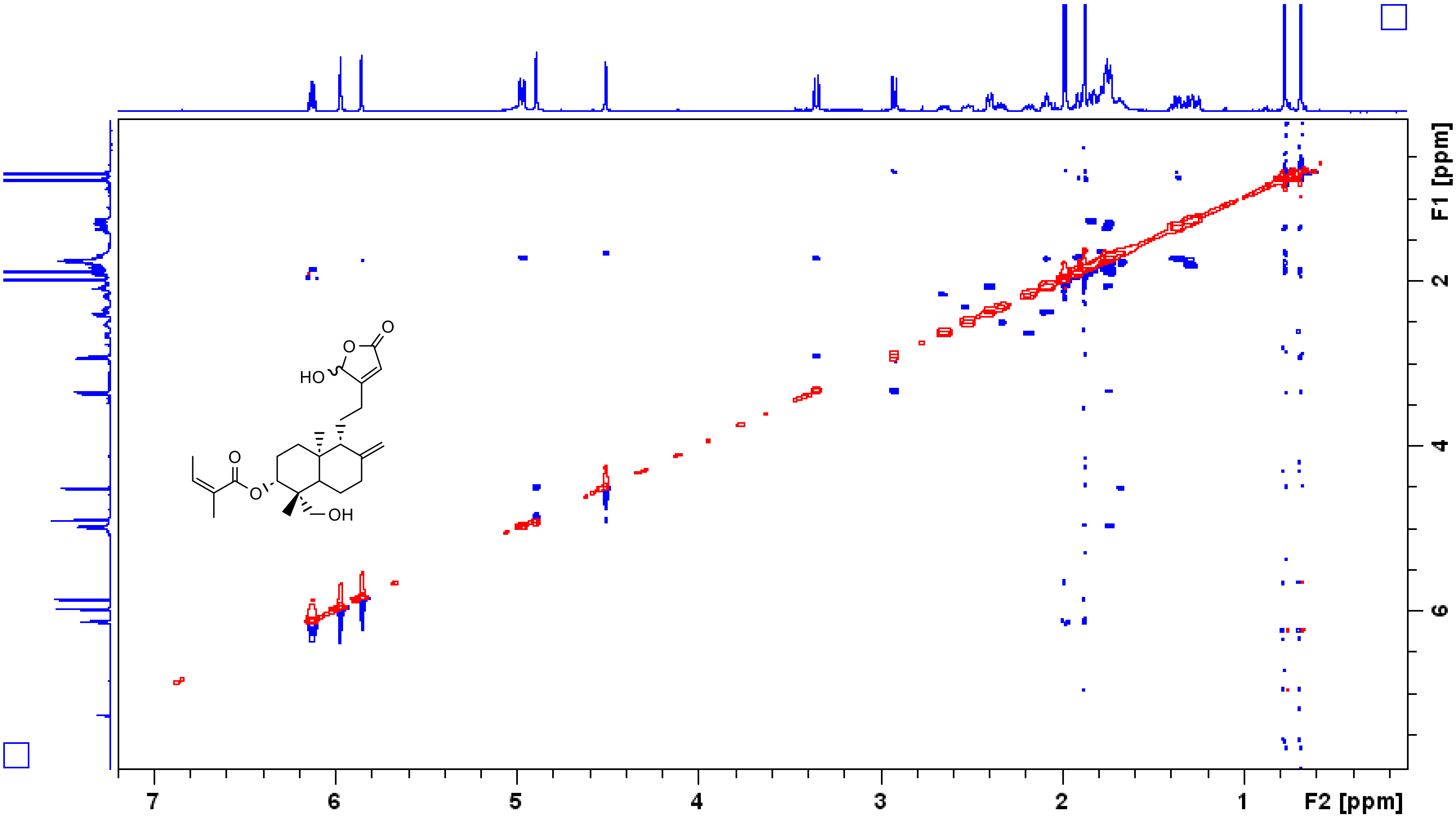

Figure S11. ^{1}H-^{1}H NOESY NMR (600 MHz, $CDCl_3$) spectrum of **26** and **27.**

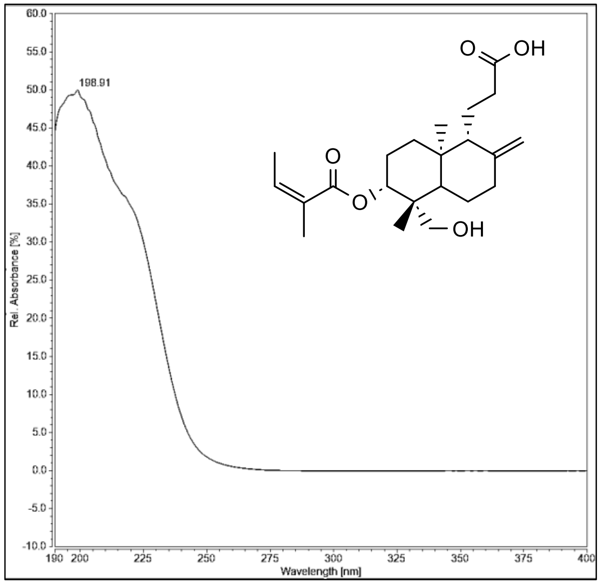

Figure S12. UV spectrum of **28**.

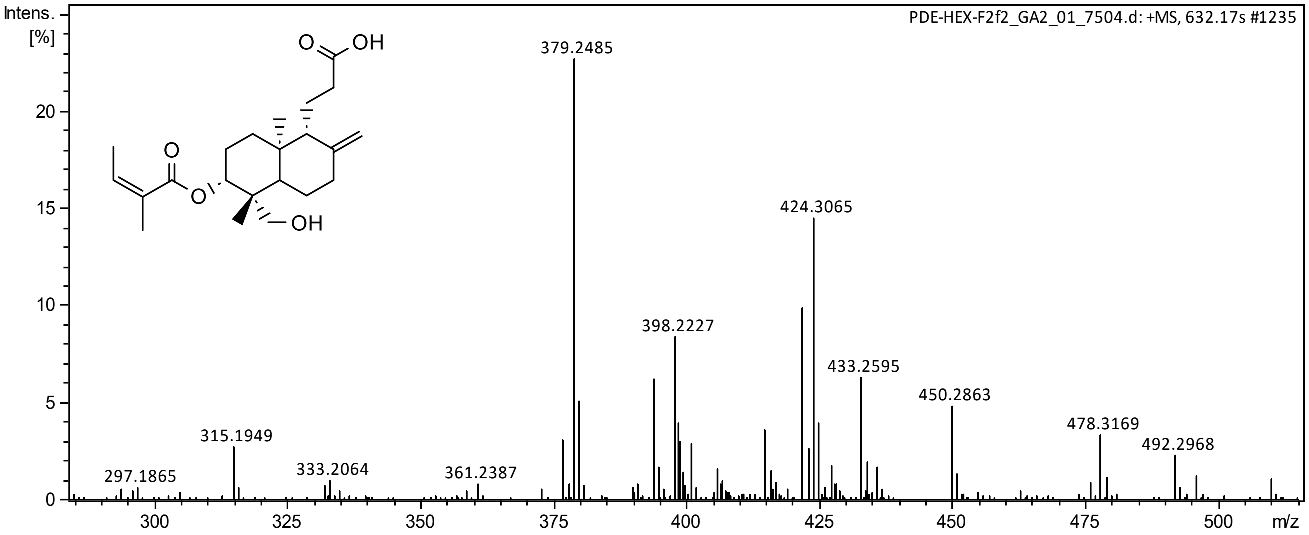

Figure S13. HRESI+MS spectrum of **28**.

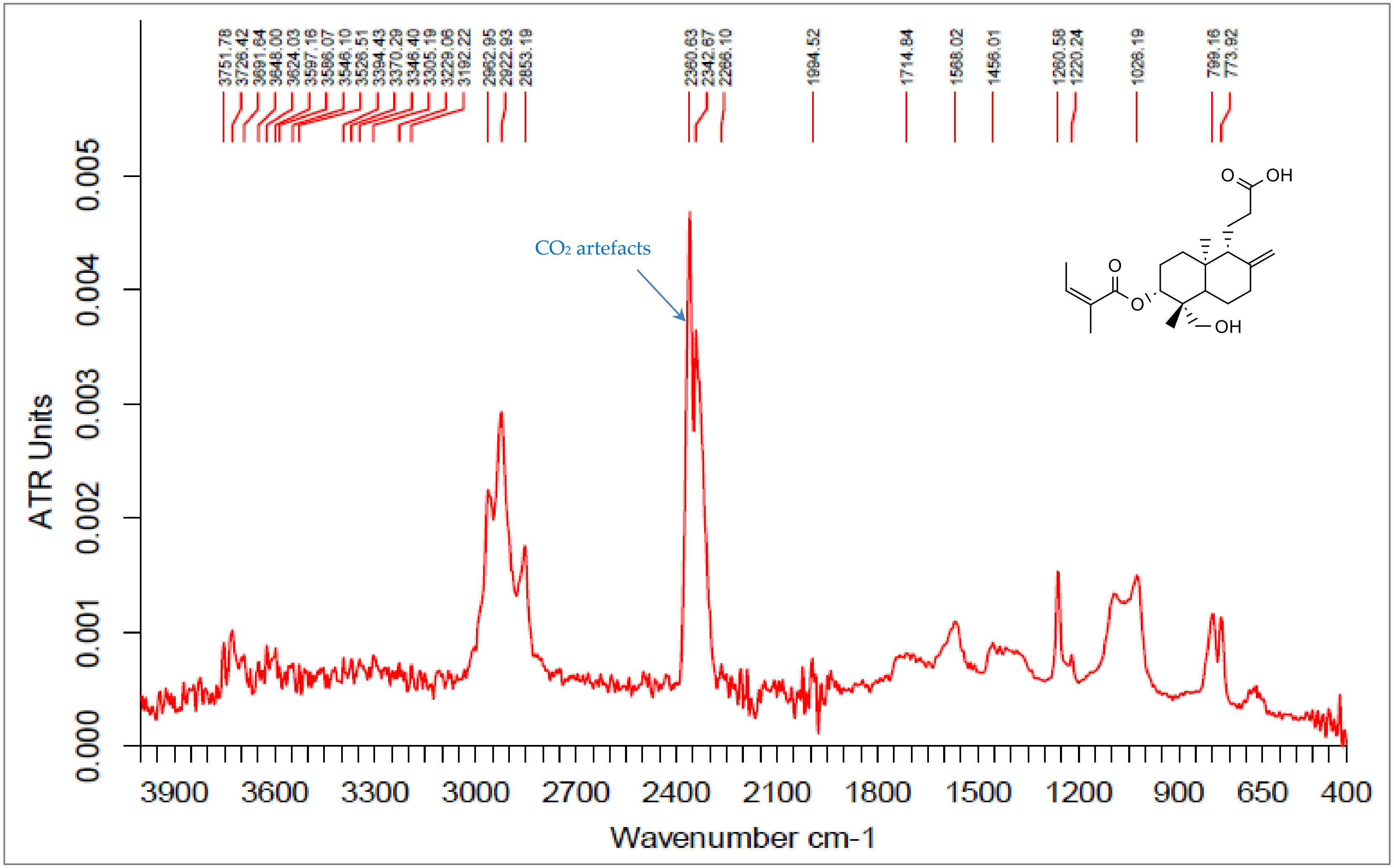

Figure S14. IR (FT-IR) spectrum of **28**.

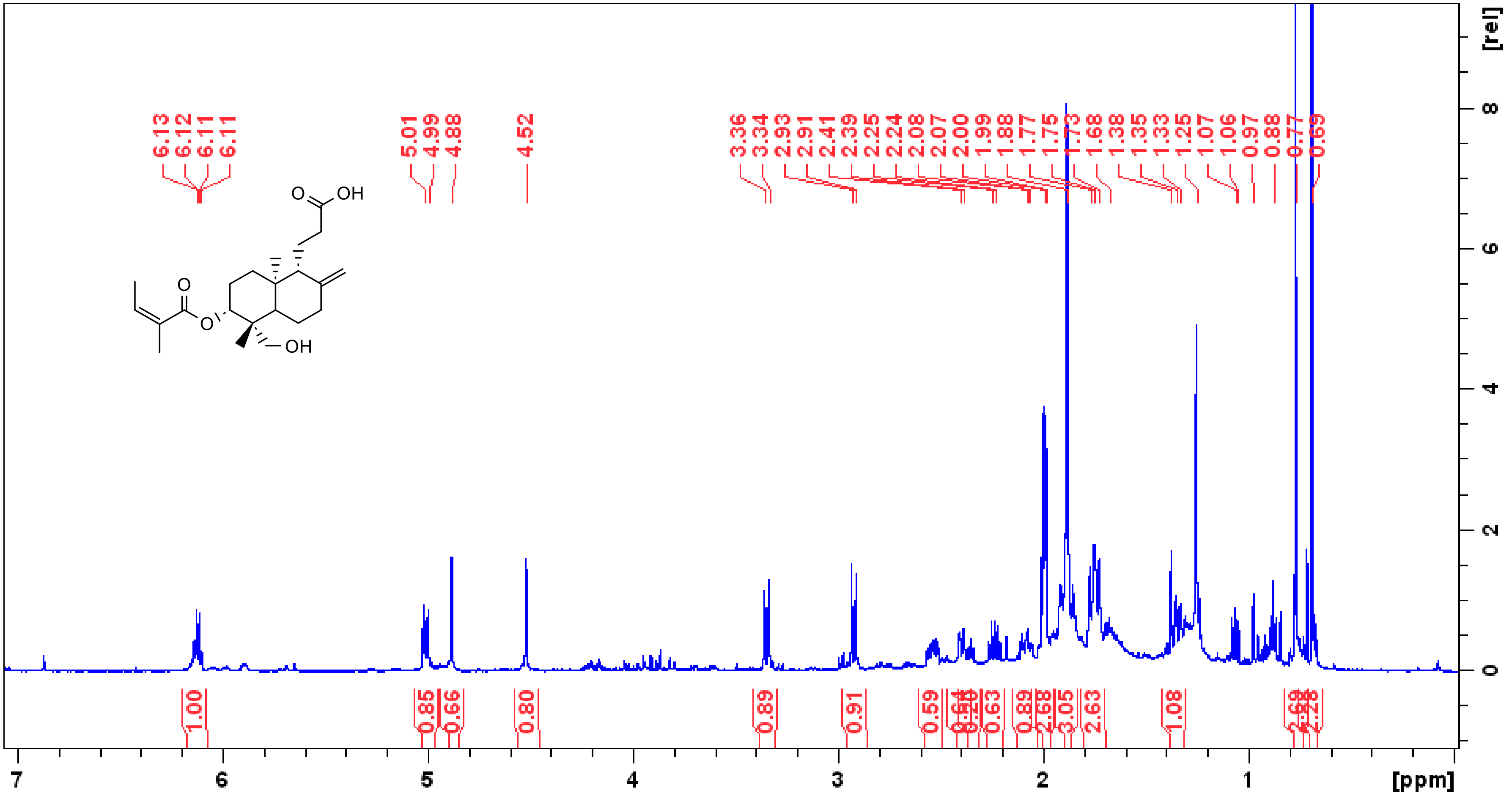

Figure S15. ^{1}H NMR ($CDCl_3$, 600 MHz) spectrum of **28**.

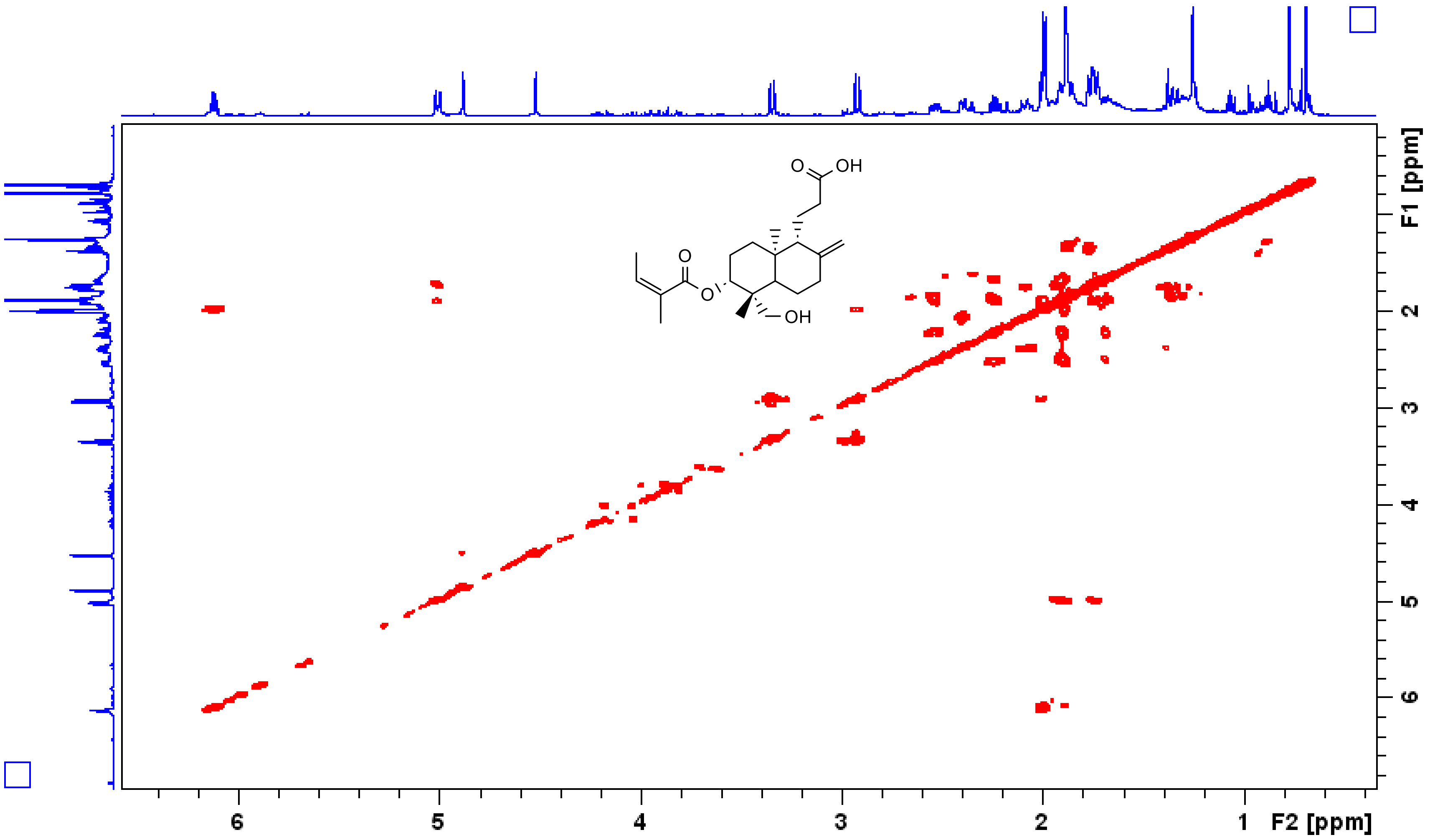

Figure S16. ¹H-¹H COSY NMR (600 MHz, $CDCl_3$) spectrum of **28**.

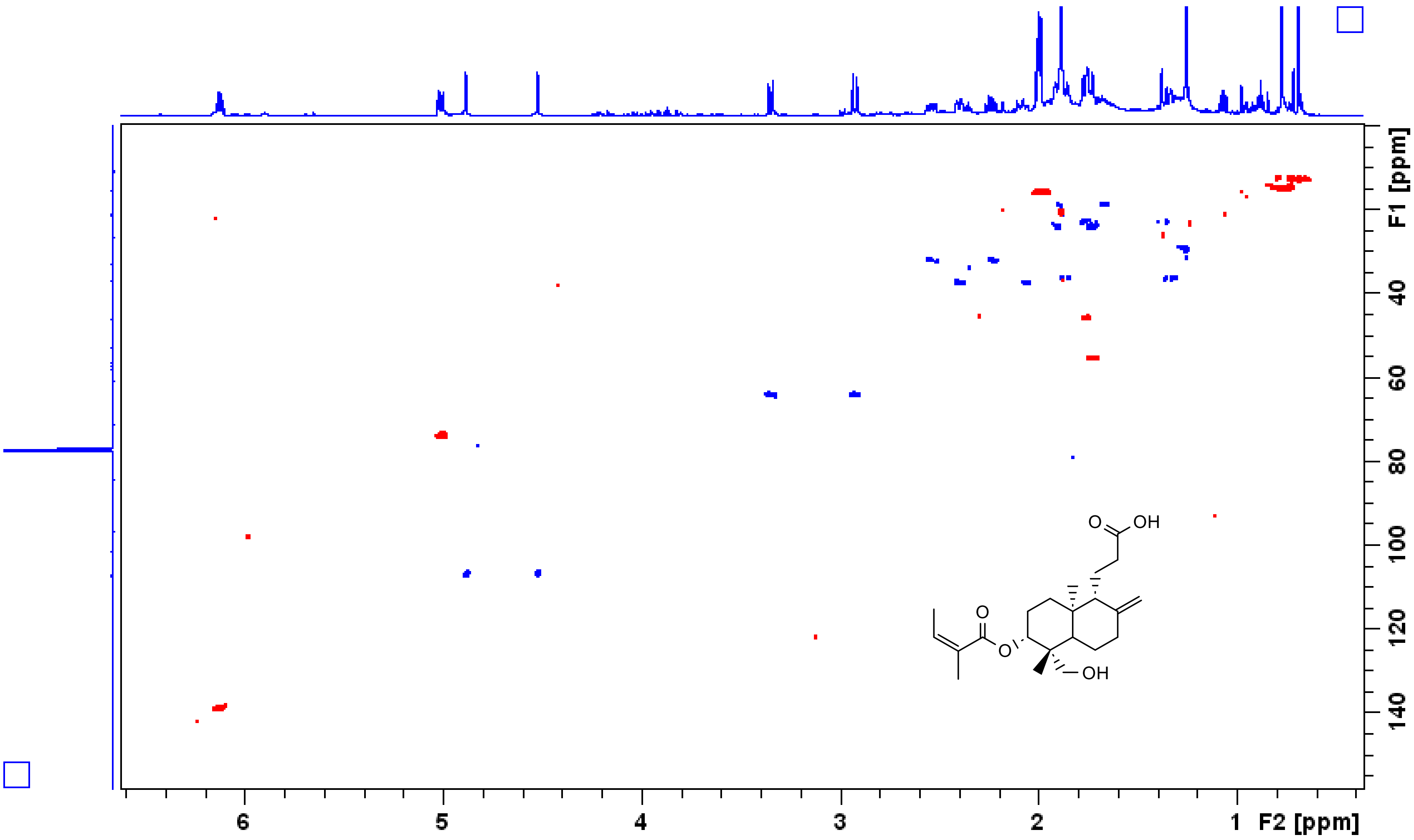

Figure S17. ¹H-¹³C HSQC NMR (600 MHz, $CDCl_3$) spectrum of **28**.

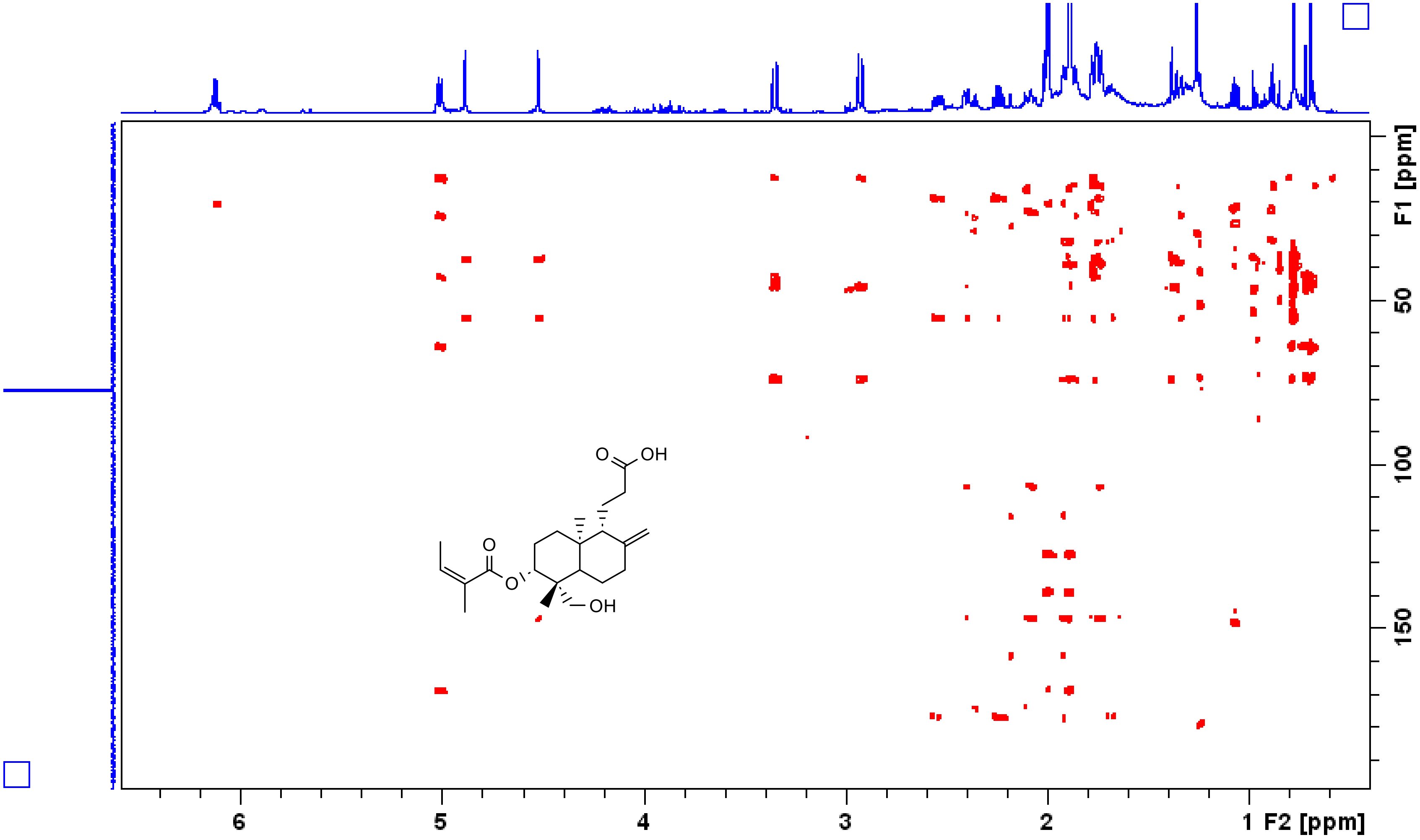

Figure S18. ^{1}H-^{13}C HMBC NMR (600 MHz, $CDCl_3$) spectrum of **28**.

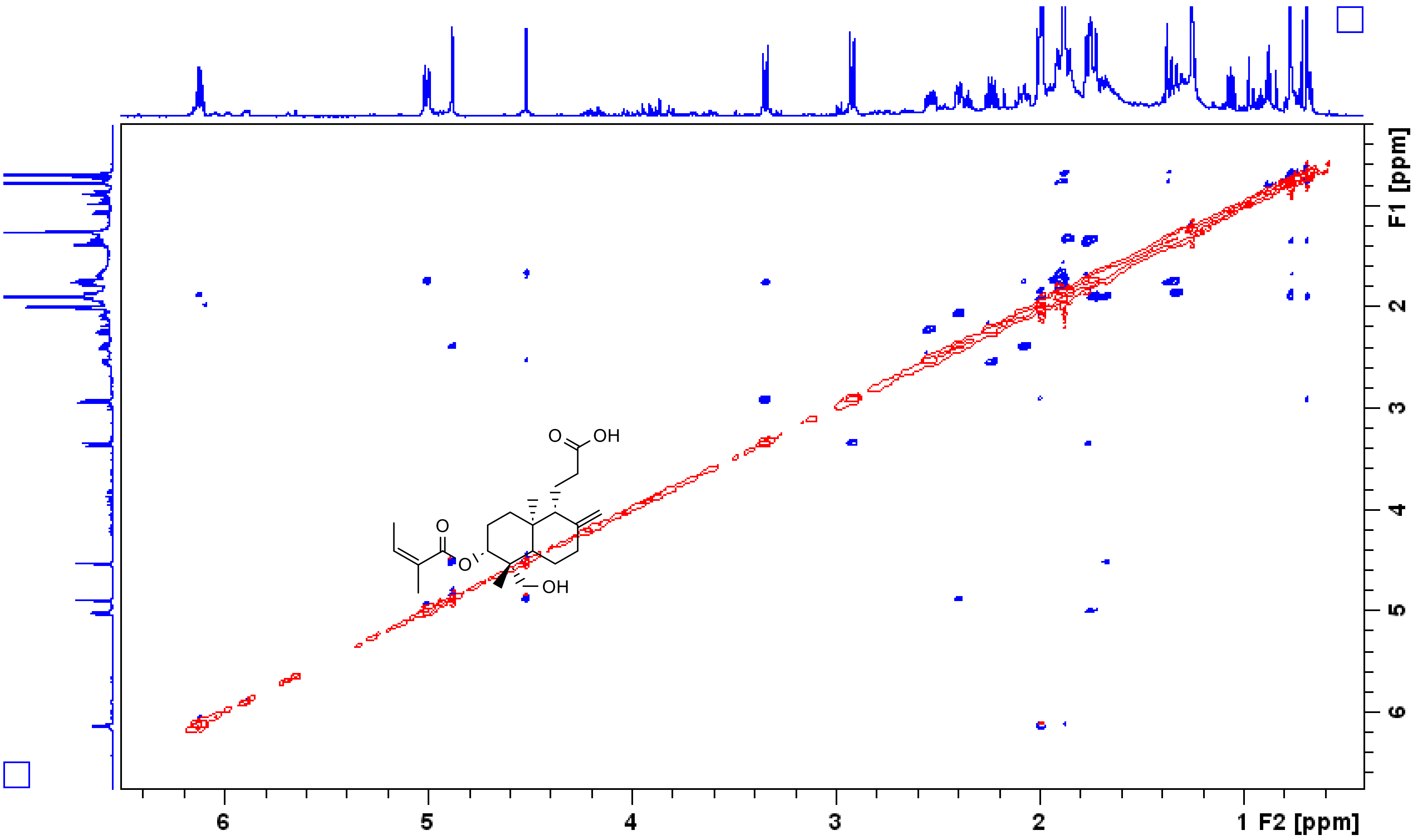

Figure S19. ^{1}H-[1]C NOESY NMR (600 MHz, $CDCl_3$) spectrum of **28**.

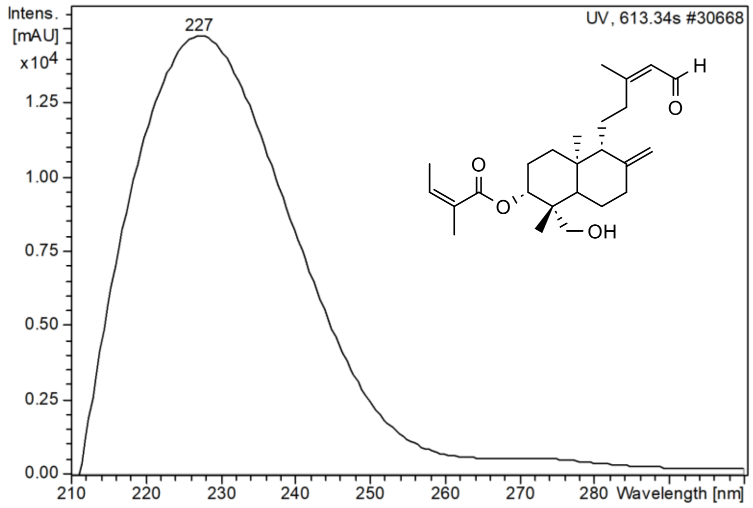

Figure S20. UV spectrum of **29**.

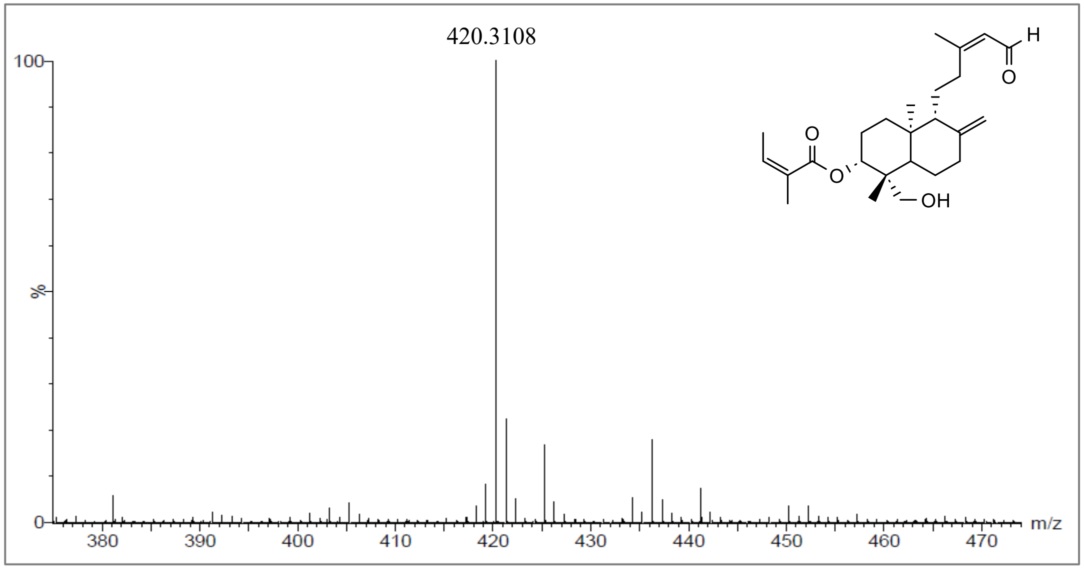

Figure S21. HRESI+MS spectrum of **29**.

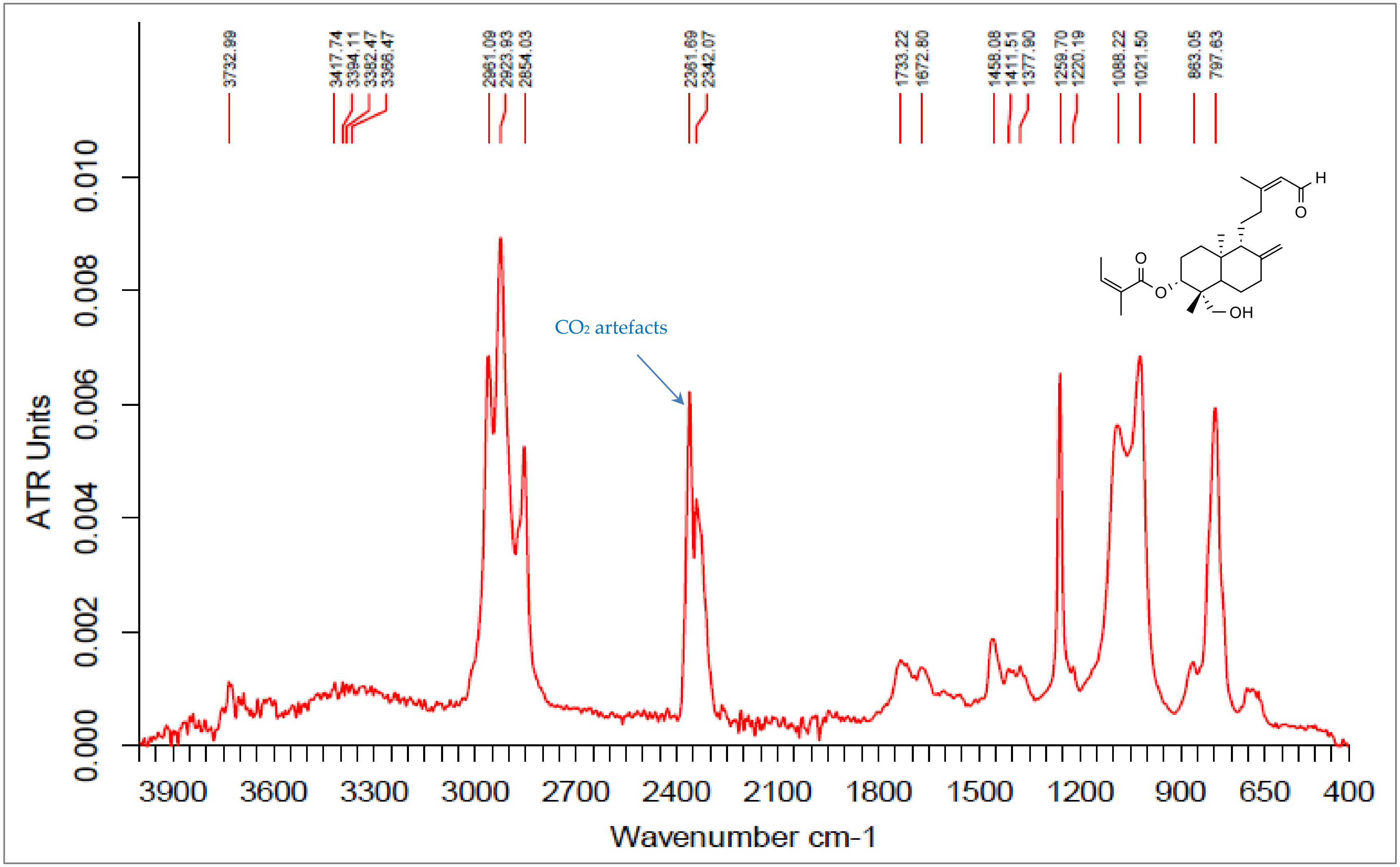

Figure S22. IR (FT-IR) spectrum of **29**.

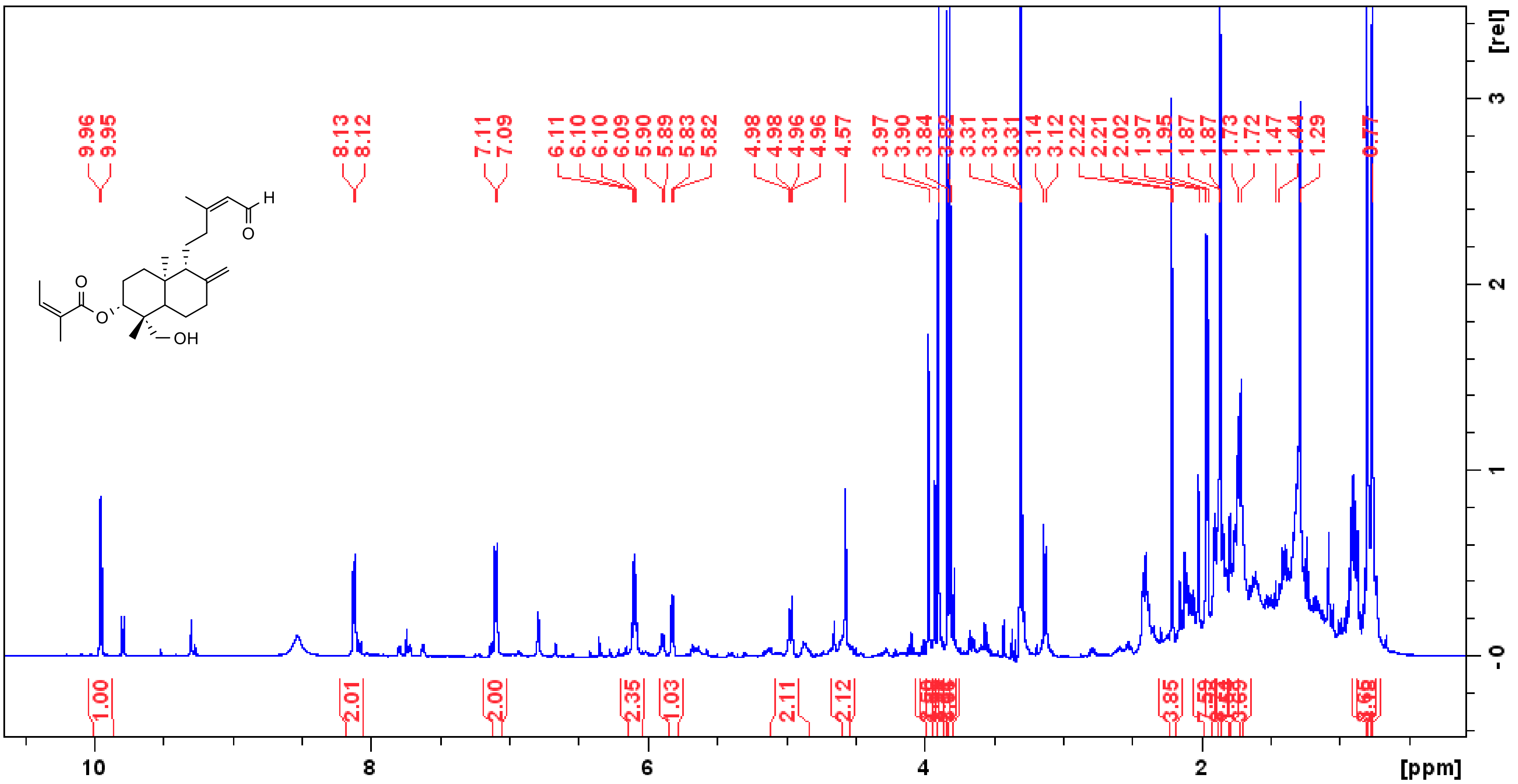

Figure S23. ^{1}H NMR (CDCl₃, 600 MHz) spectrum of **29**.

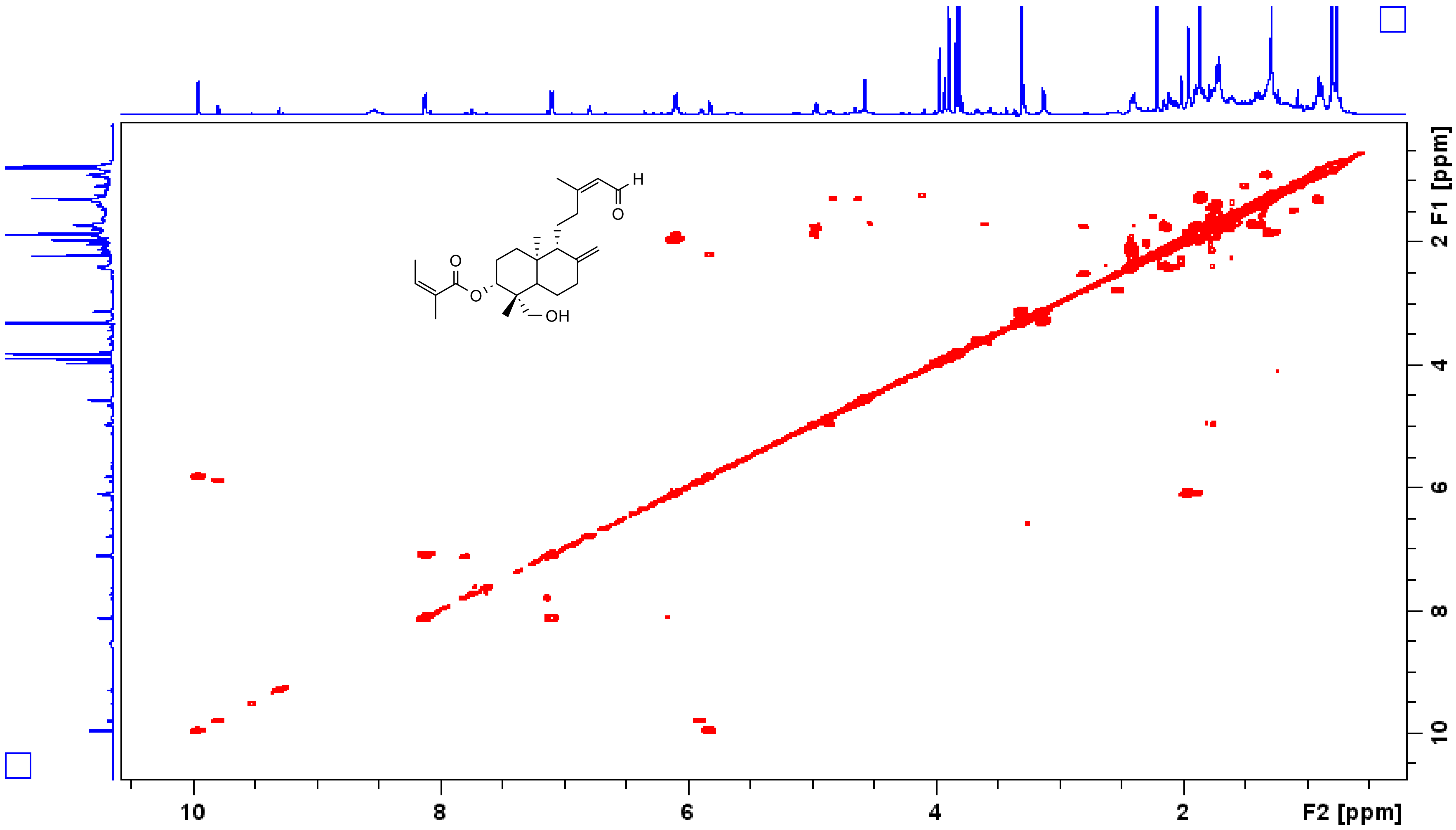

Figure S24. ^{1}H-^{1}H COSY NMR (600 MHz, $CDCl_3$) spectrum of **29**.

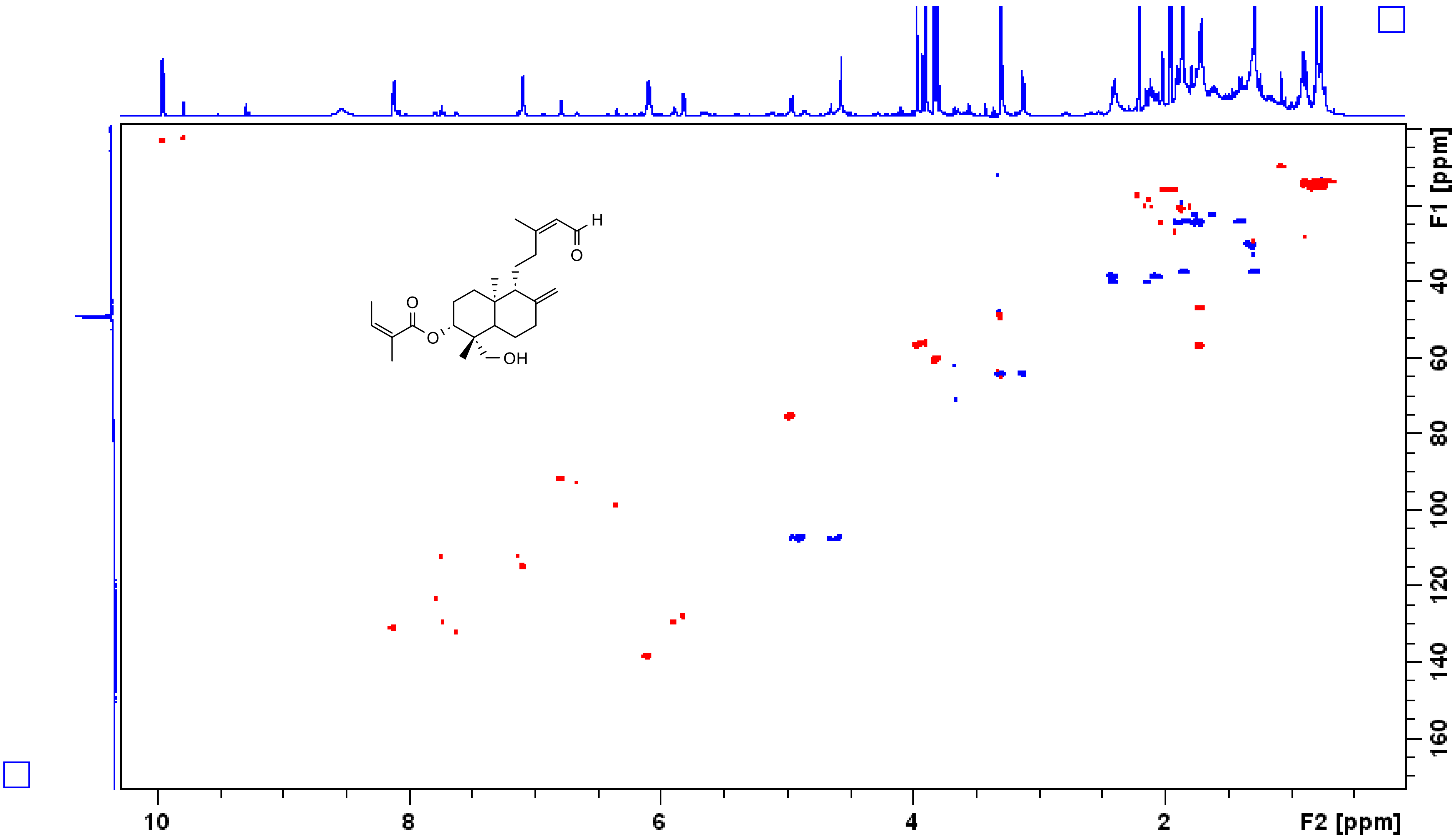

Figure S25. ^{1}H-^{13}C HSQC NMR (600 MHz, $CDCl_3$) spectrum of **29**.

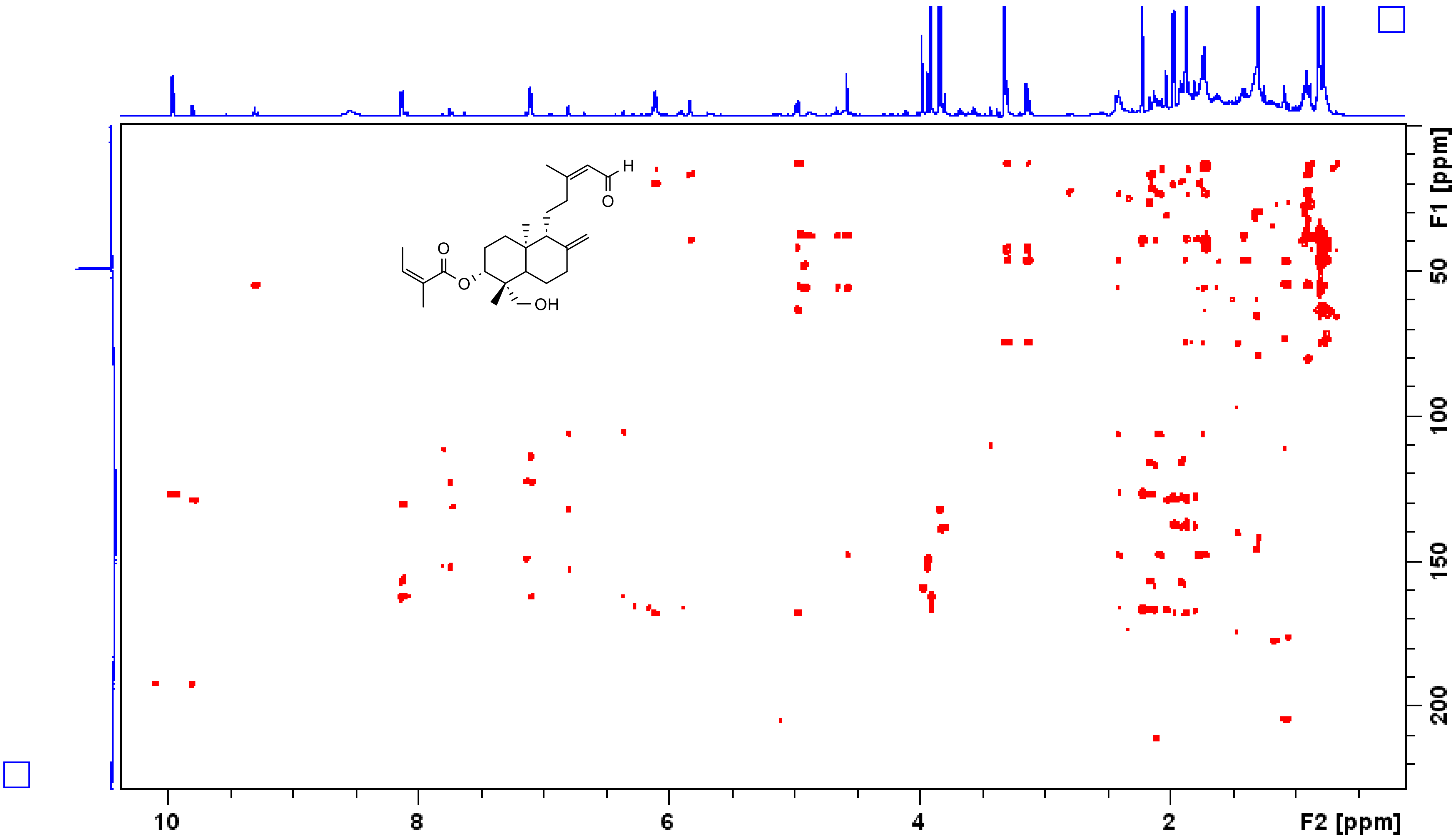

Figure S26. ^{1}H-^{13}C HMBC NMR (600 MHz, $CDCl_3$) spectrum of **29**.

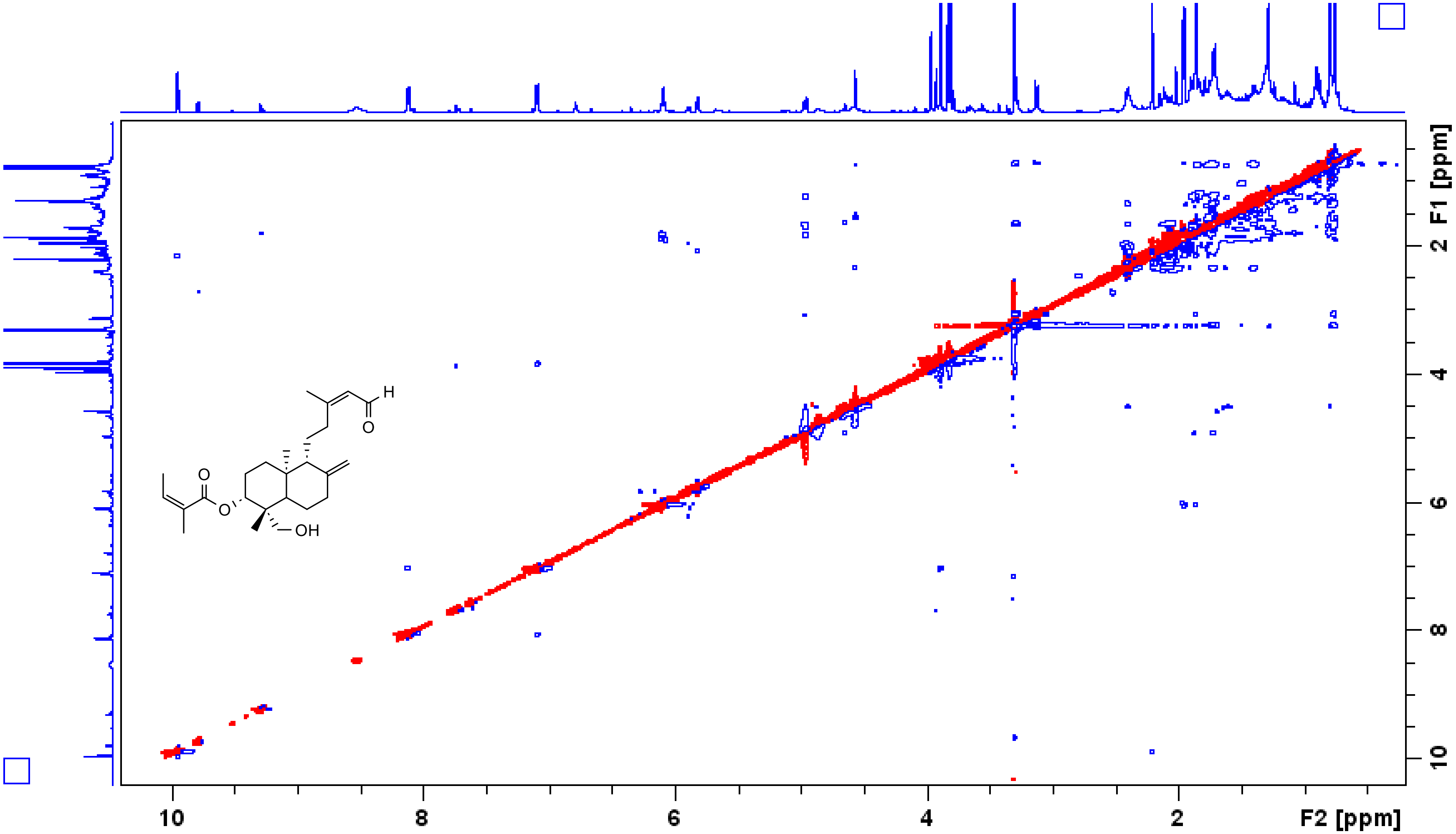

Figure S27. ¹H-¹C NOESY NMR (600 MHz, CDCl₃) spectrum of **29**.

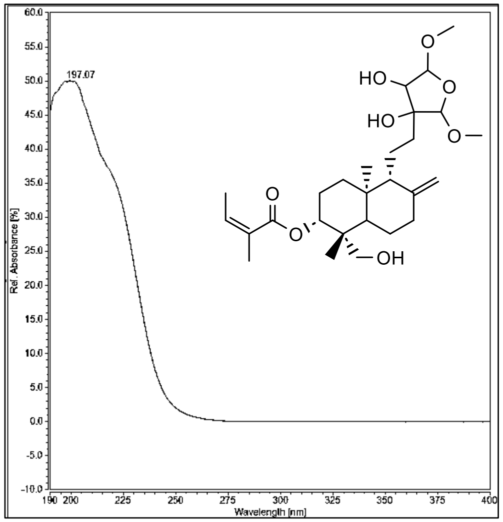

Figure S28. UV spectrum of **30**.

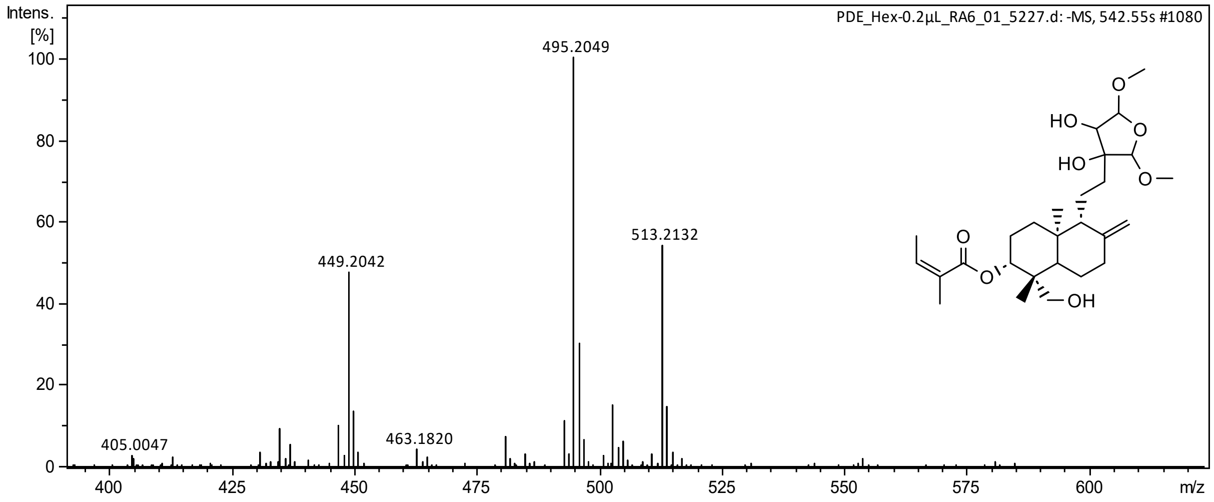

Figure S29. HRESI-MS spectrum of **30**.

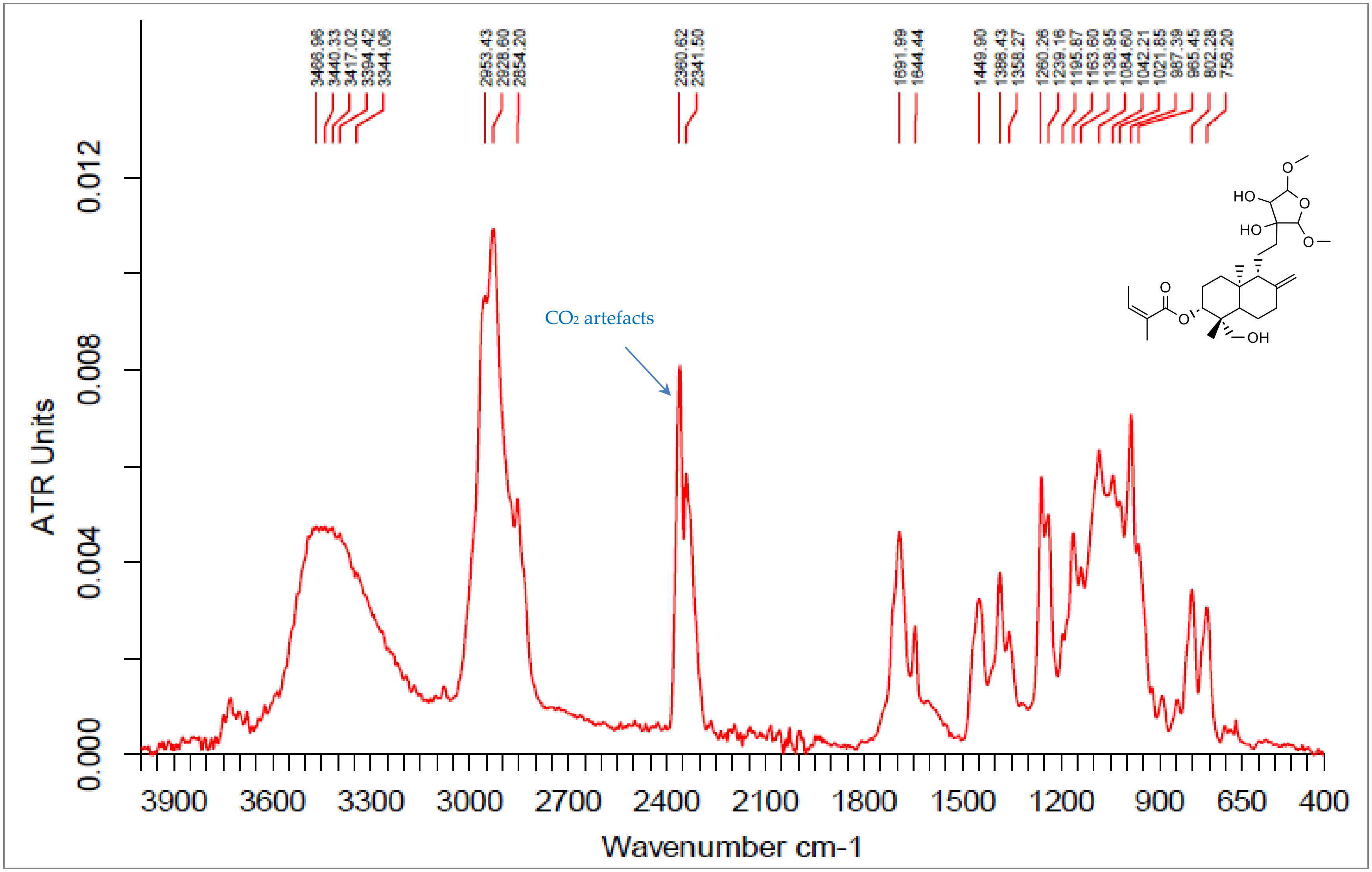

Figure S30. IR (FT-IR) spectrum of **30**.

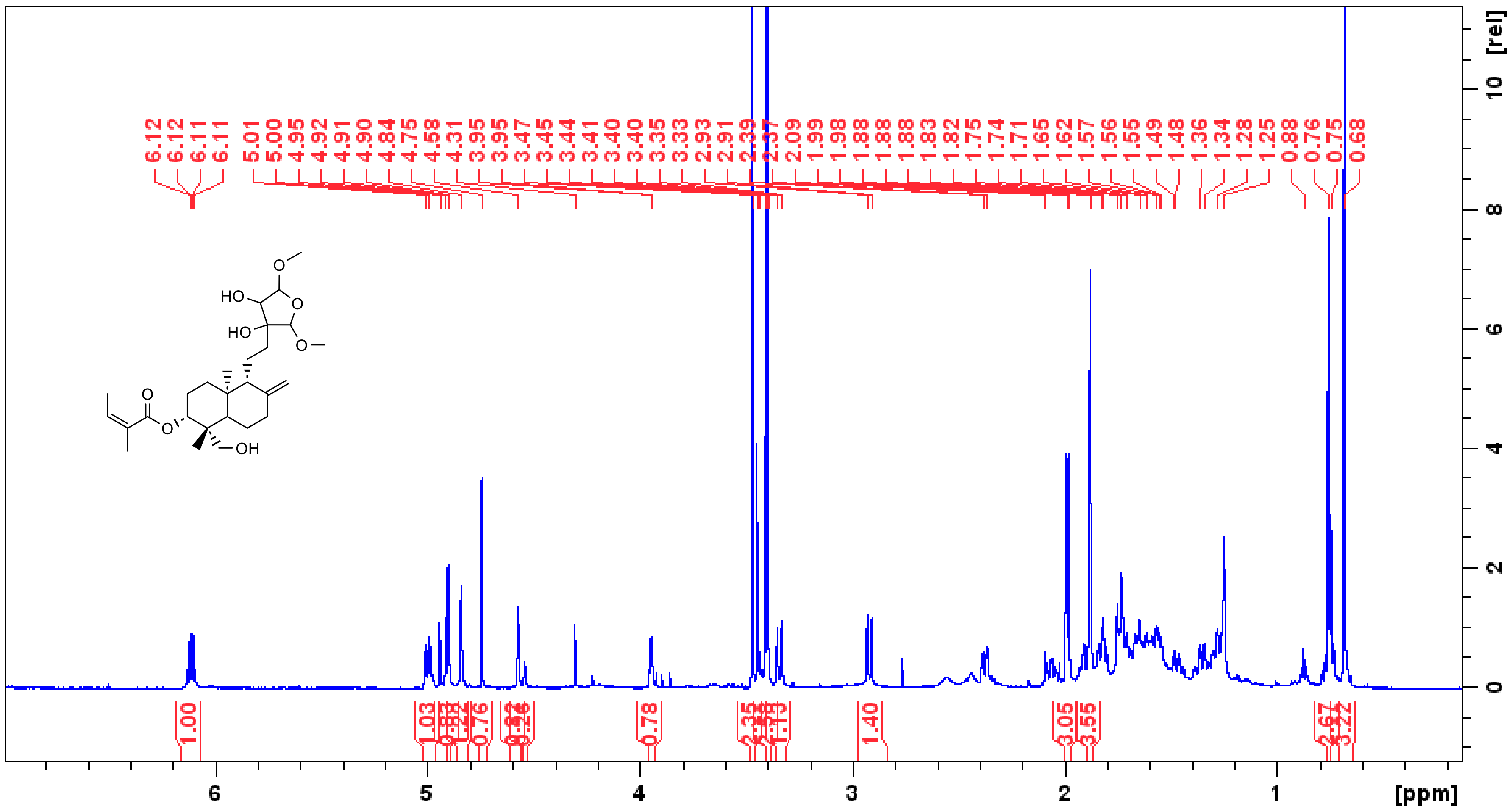

Figure S31. ^{1}H NMR ($CDCl_3$, 600 MHz) spectrum of **30**.

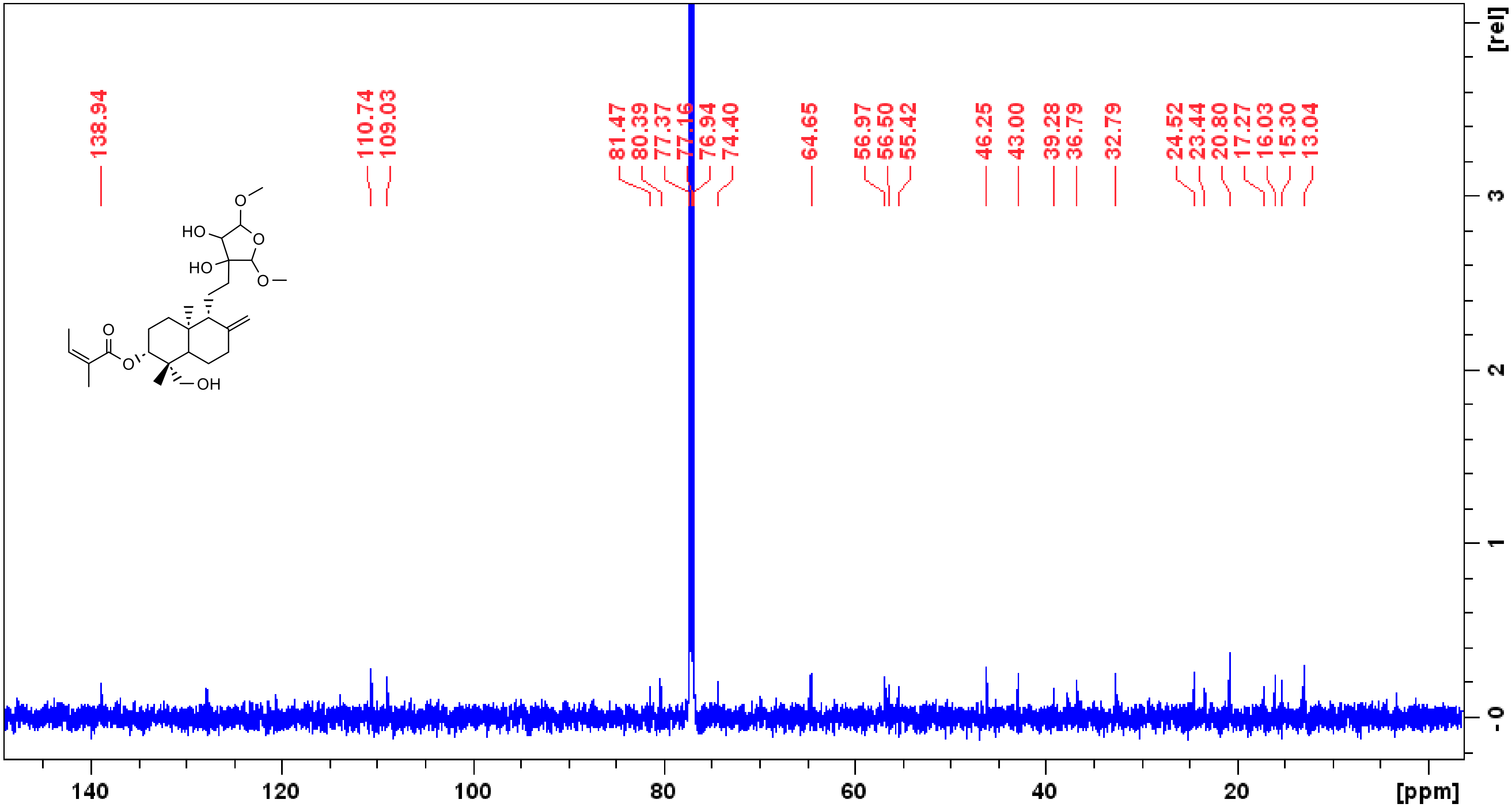

Figure S32. ^{13}C NMR ($CDCl_3$, 150 MHz) spectrum of **30**.

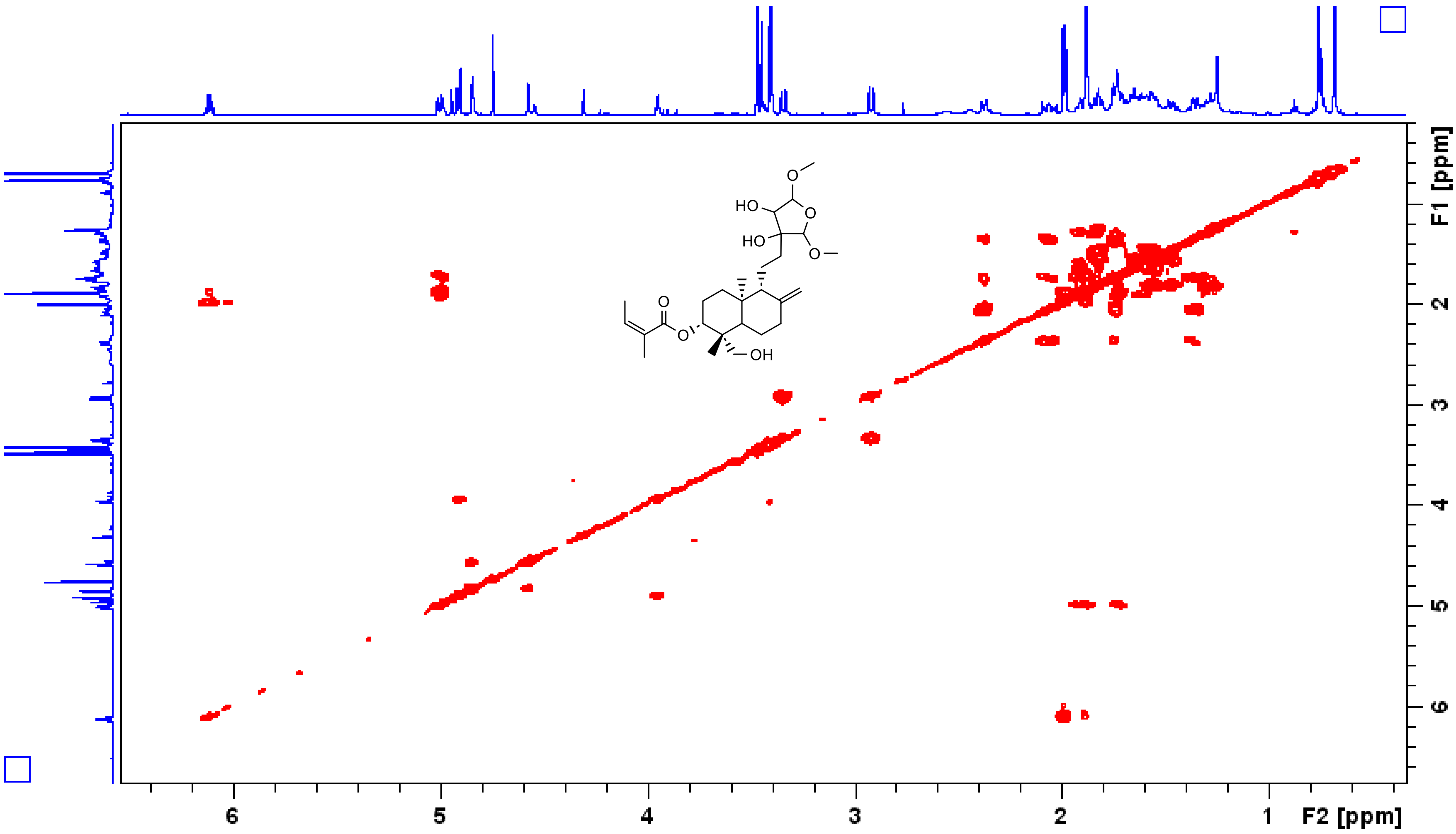

Figure S33. ^{1}H-^{1}H COSY NMR (600 MHz, $CDCl_3$) spectrum of **30**.

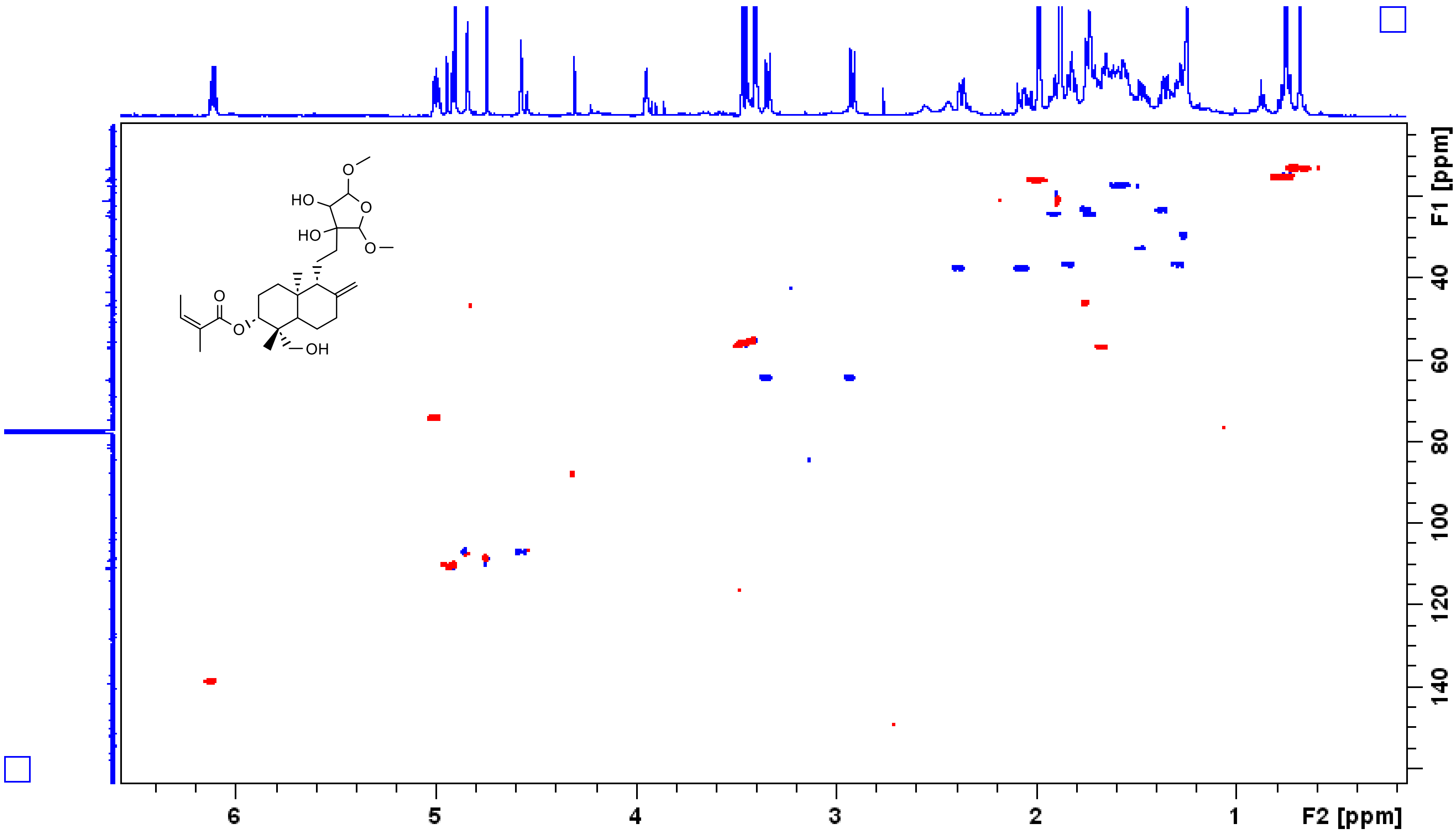

Figure S34. ^{1}H-^{13}C HSQC NMR (600 MHz, $CDCl_3$) spectrum of **30**.

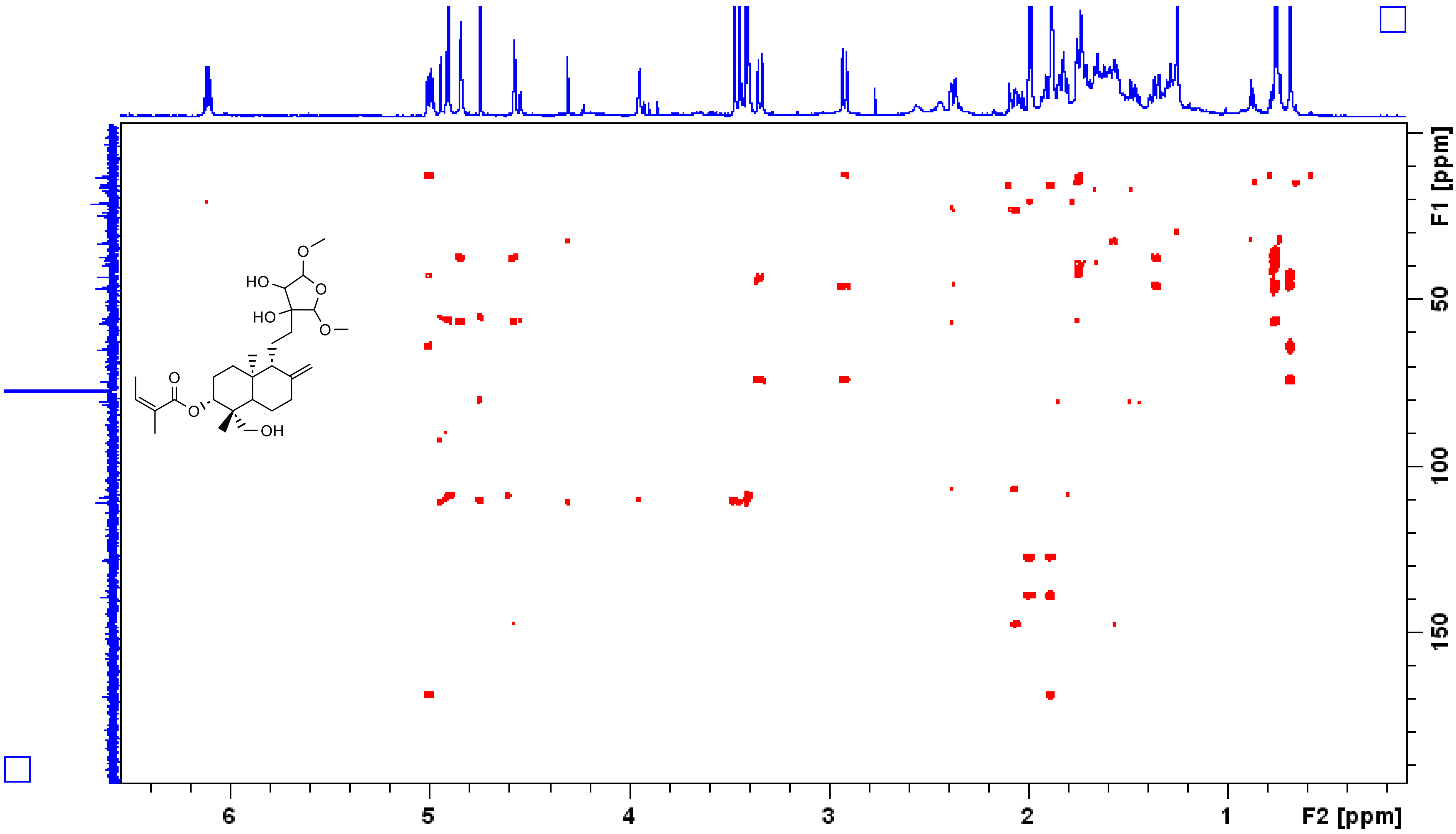

Figure S35. ^{1}H-^{13}C HMBC NMR (600 MHz, $CDCl_3$) spectrum of **30**.

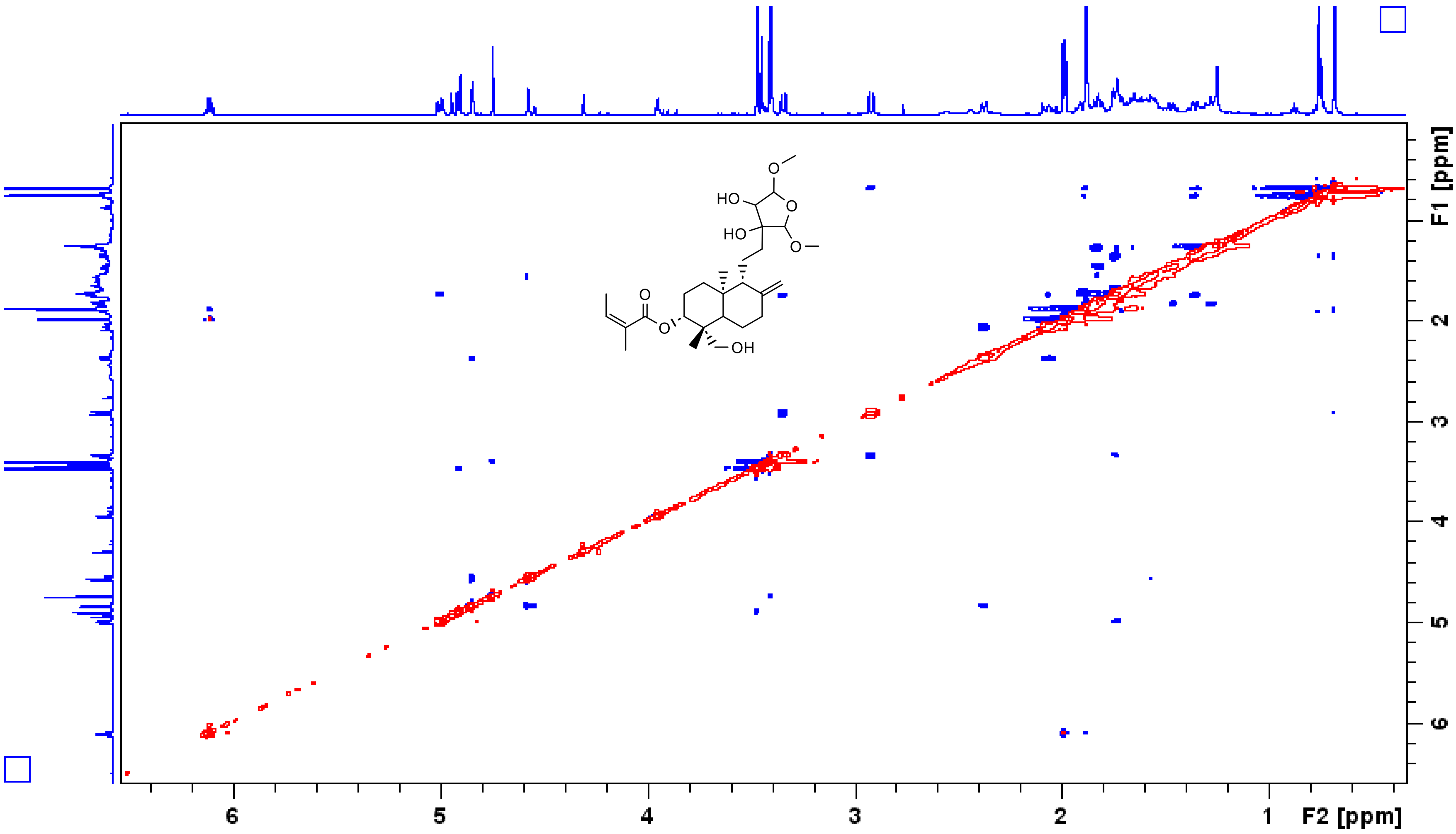

Figure S36. ^{1}H-^{1}H NOESY NMR (600 MHz, $CDCl_3$) spectrum of **30**.

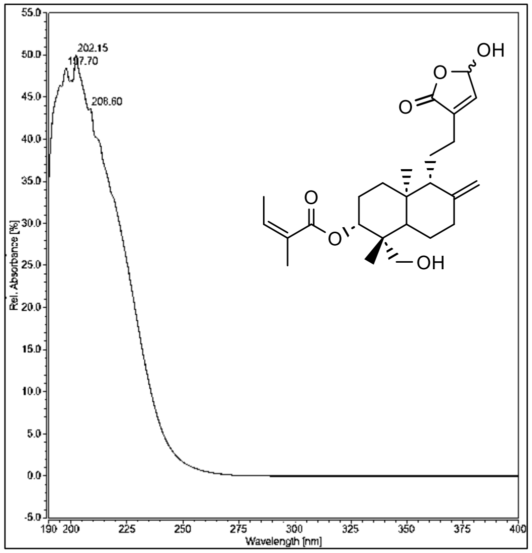

Figure S37. UV spectrum of **31** and **32**.

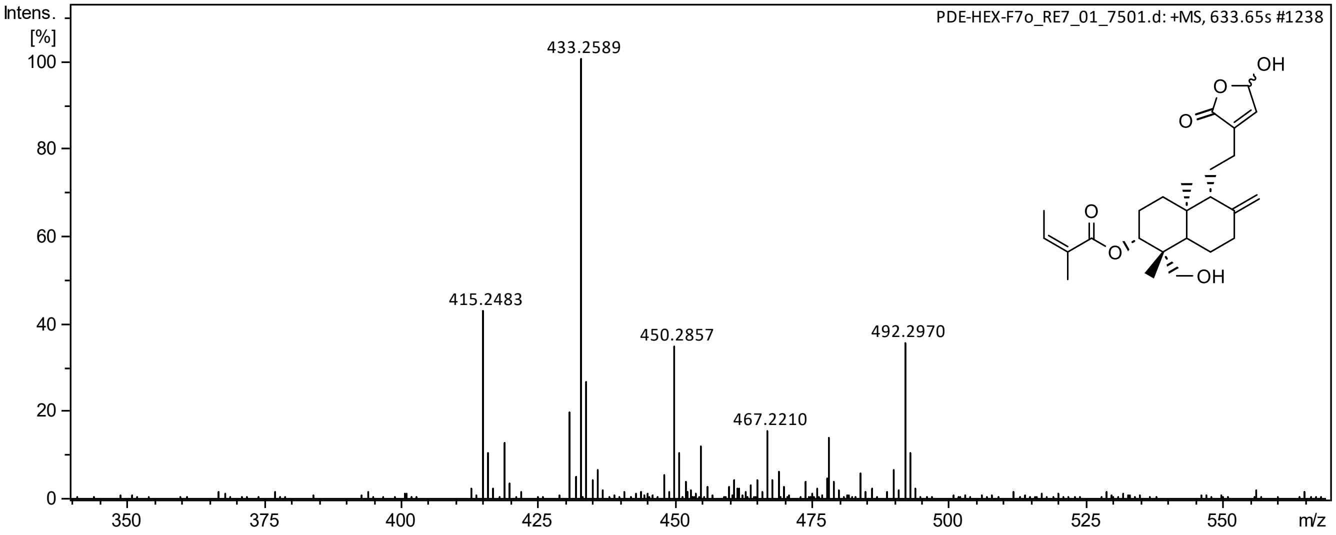

Figure S38. HRESI+MS spectrum of **31** and **32**.

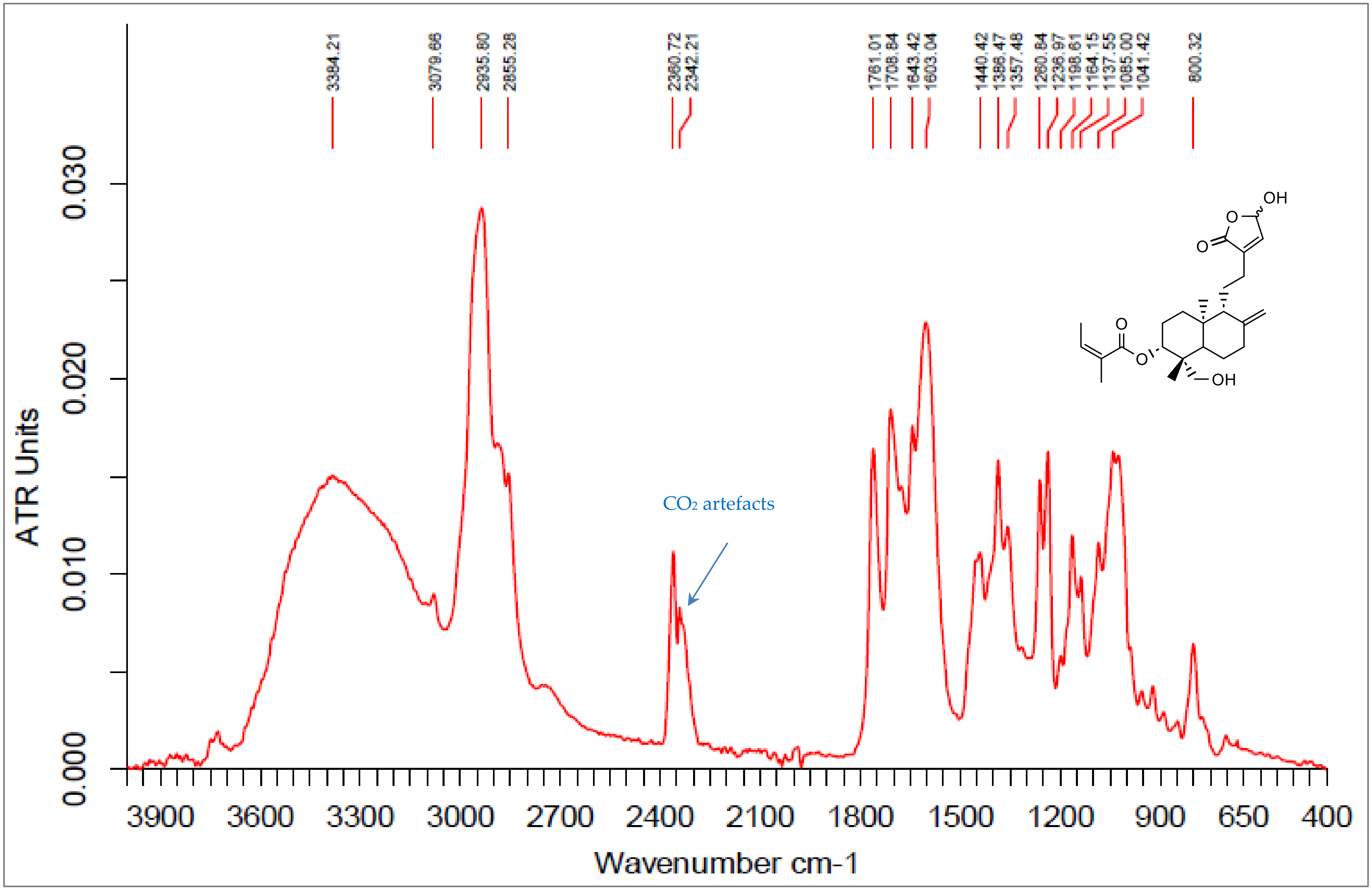

Figure S39. IR (FT-IR) spectrum of **31** and **32**.

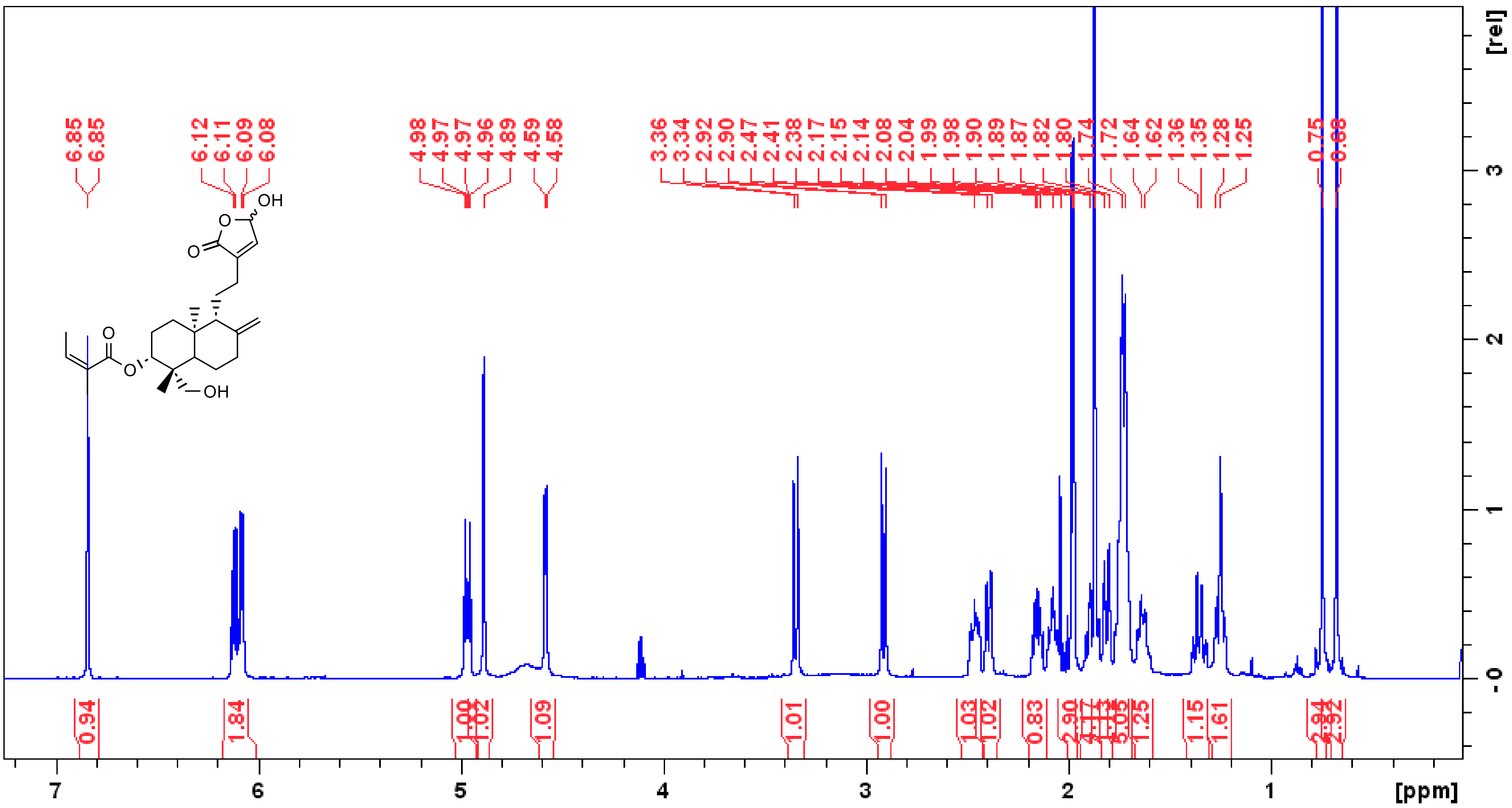

Figure S40. ^{1}H NMR ($CDCl_3$, 600 MHz) spectrum of **31** and **32**.

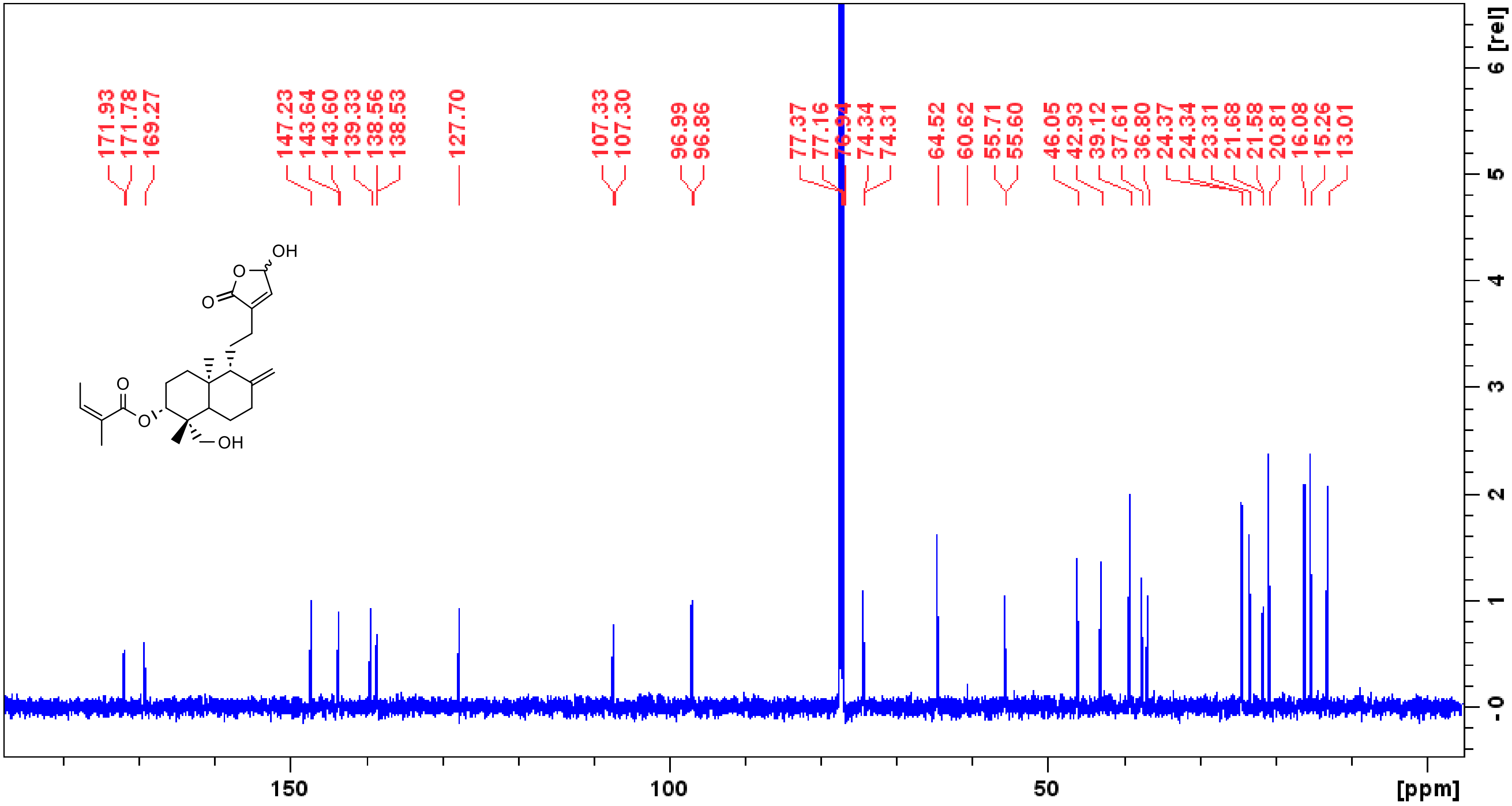

Figure S41. ${}^{13}C$ NMR ($CDCl_3$, 150 MHz) spectrum of **31** and **32**.

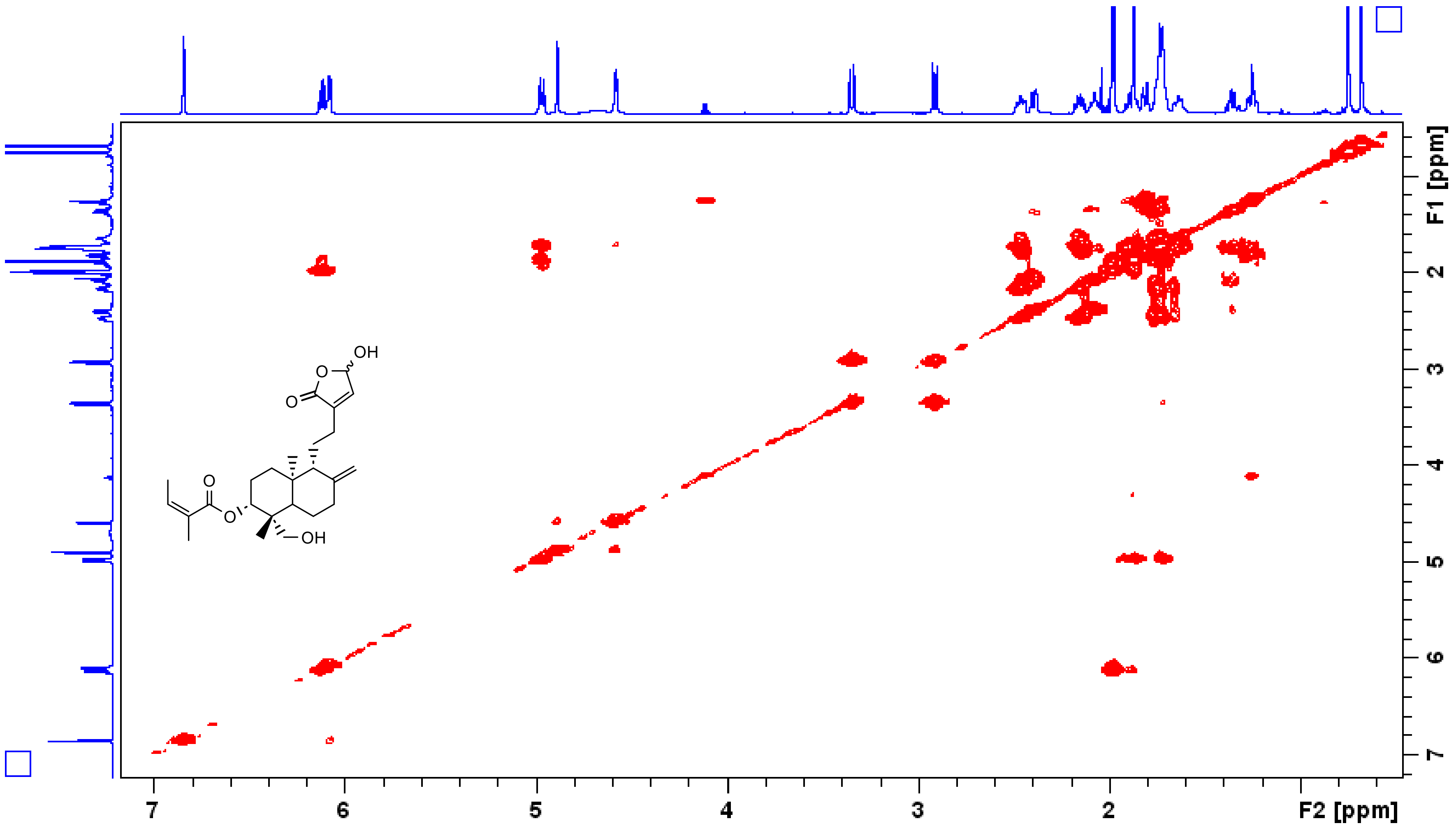

Figure S42. ¹H-¹H COSY NMR (600 MHz, $CDCl_3$) spectrum of **31** and **32**.

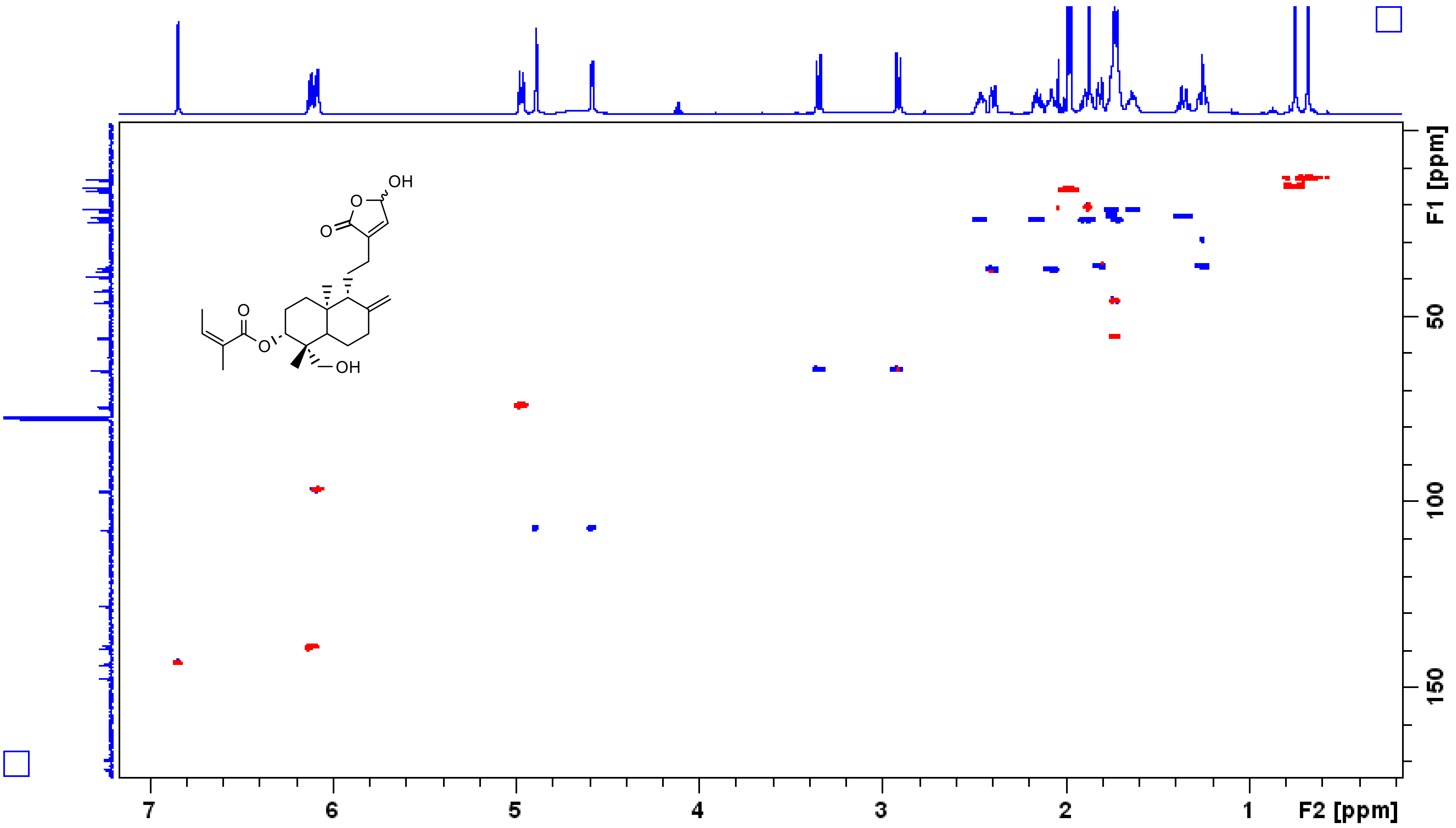

Figure S43. ^{1}H-^{13}C HSQC NMR (600 MHz, $CDCl_3$) spectrum of **31** and **32**.

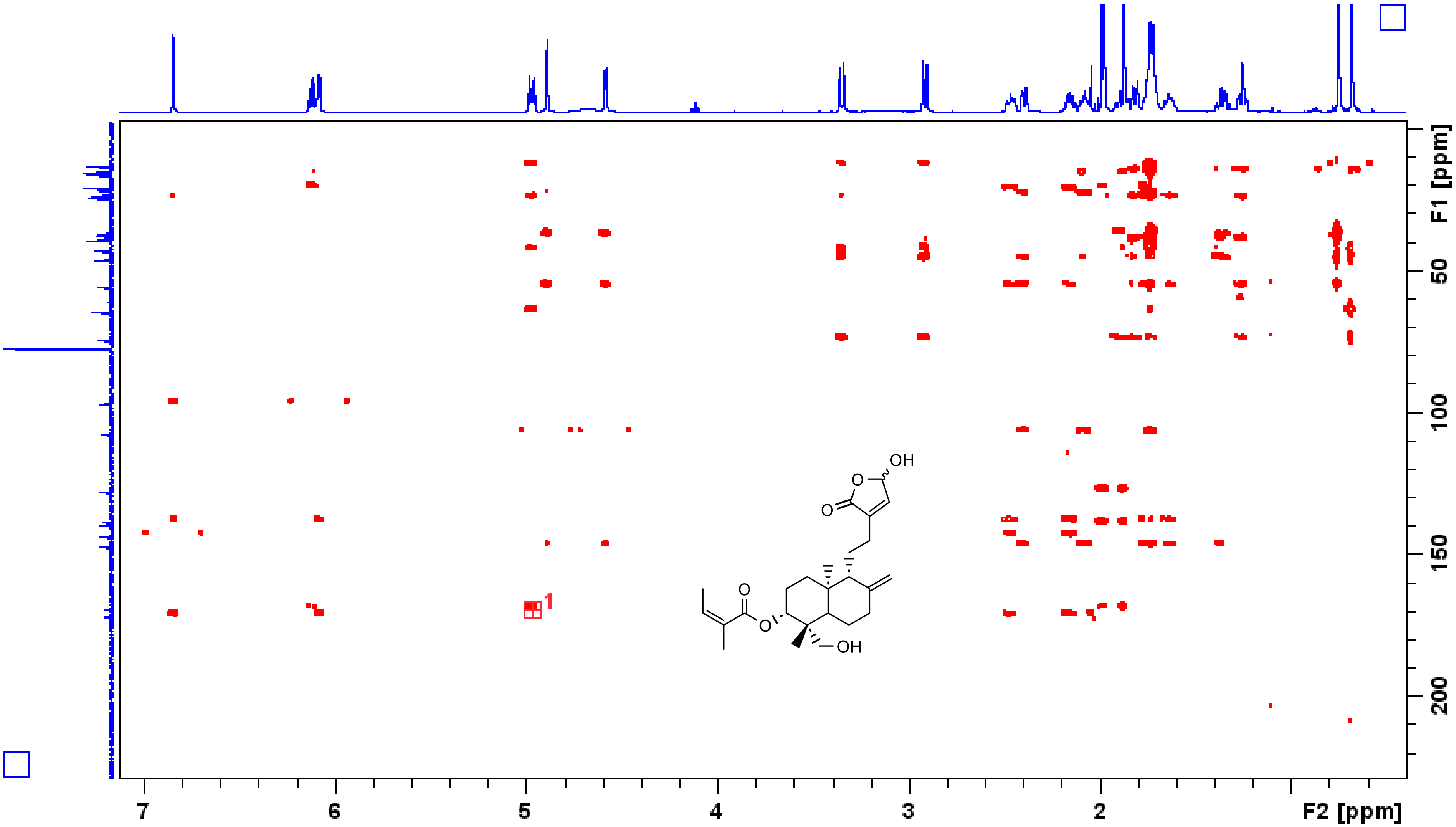

Figure S44. ^{1}H-^{13}C HMBC NMR (600 MHz, $CDCl_3$) spectrum of **31** and **32**.

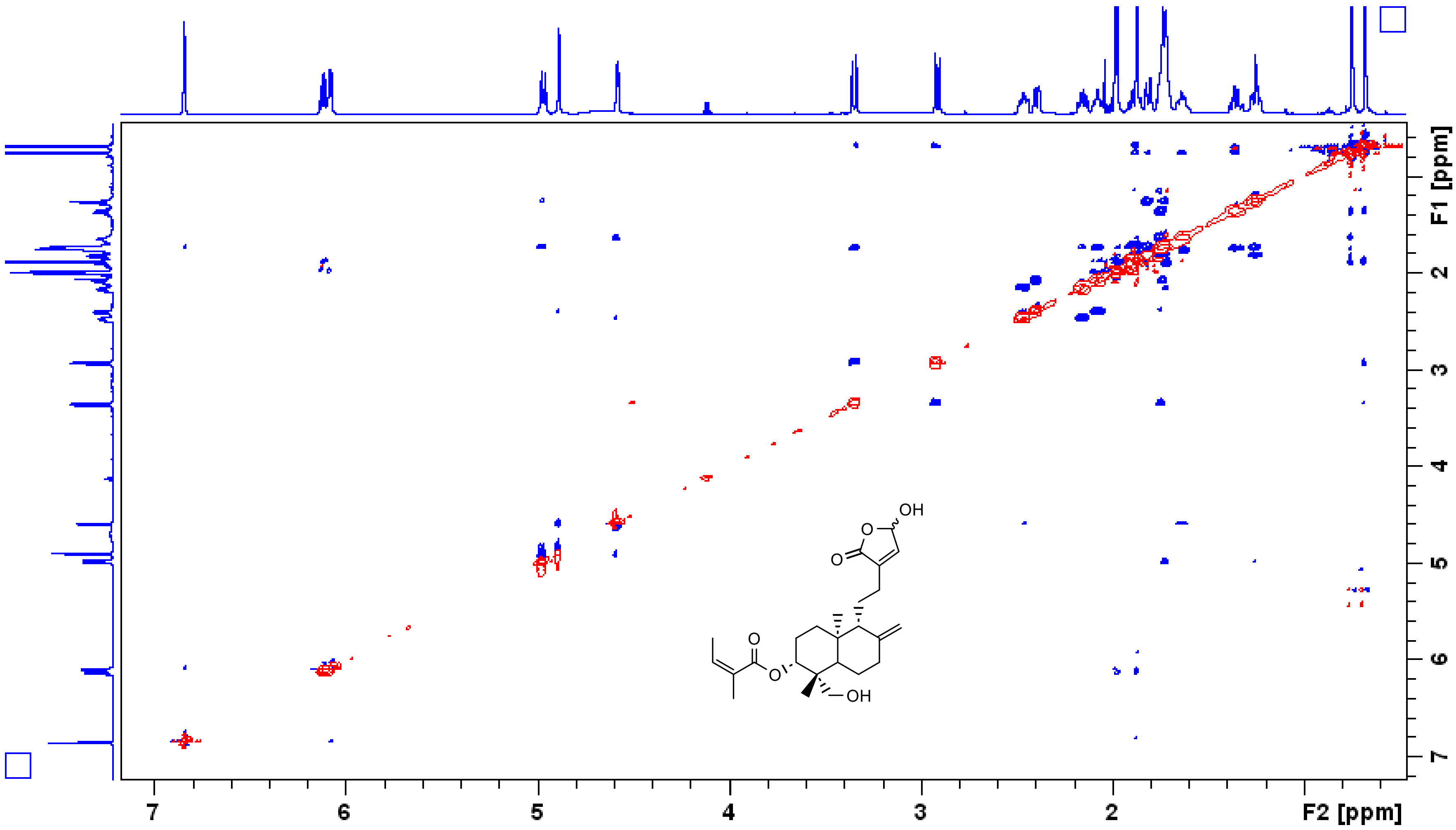

Figure S45. ^{1}H-^{1}H NOESY NMR (600 MHz, $CDCl_3$) spectrum of **31** and **32**.

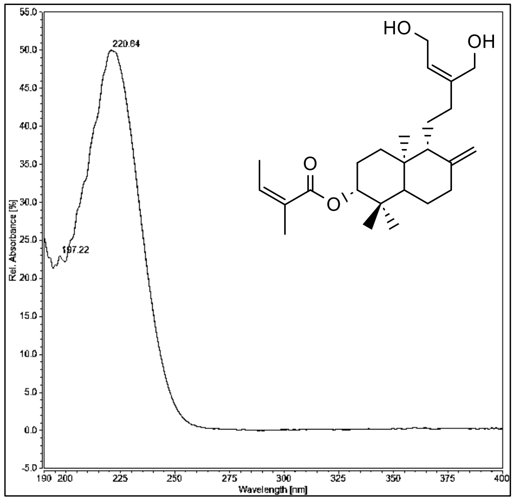

Figure S46. UV spectrum of **33**.

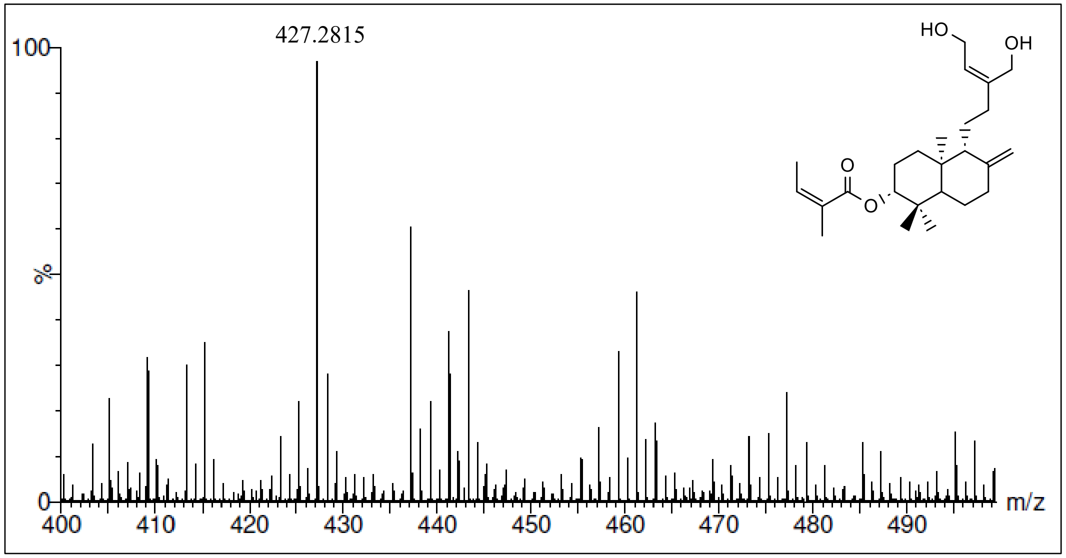

Figure S47. HRESI+MS spectrum of **33**.

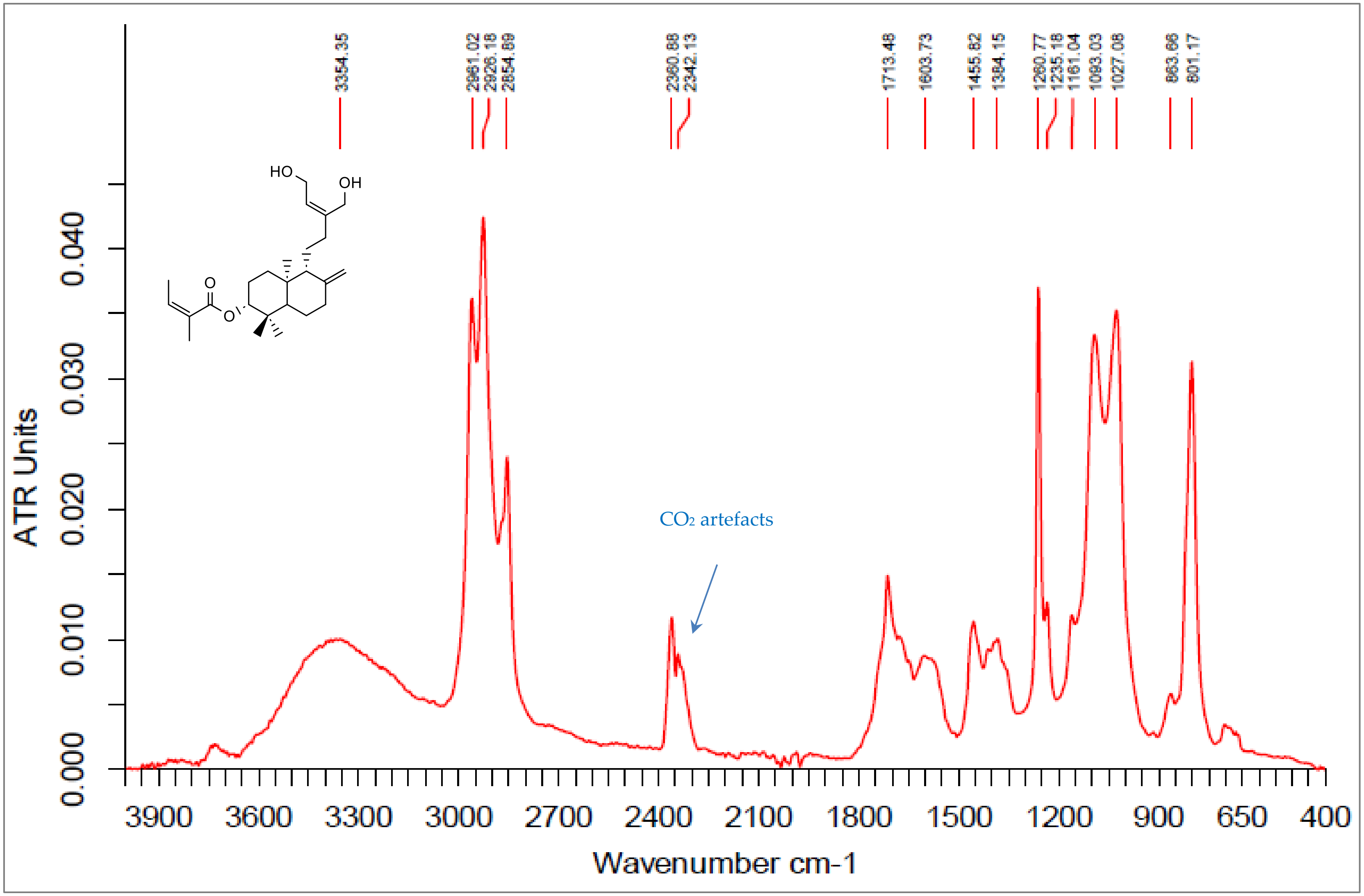

Figure S48. IR (FT-IR) spectrum of **33**.

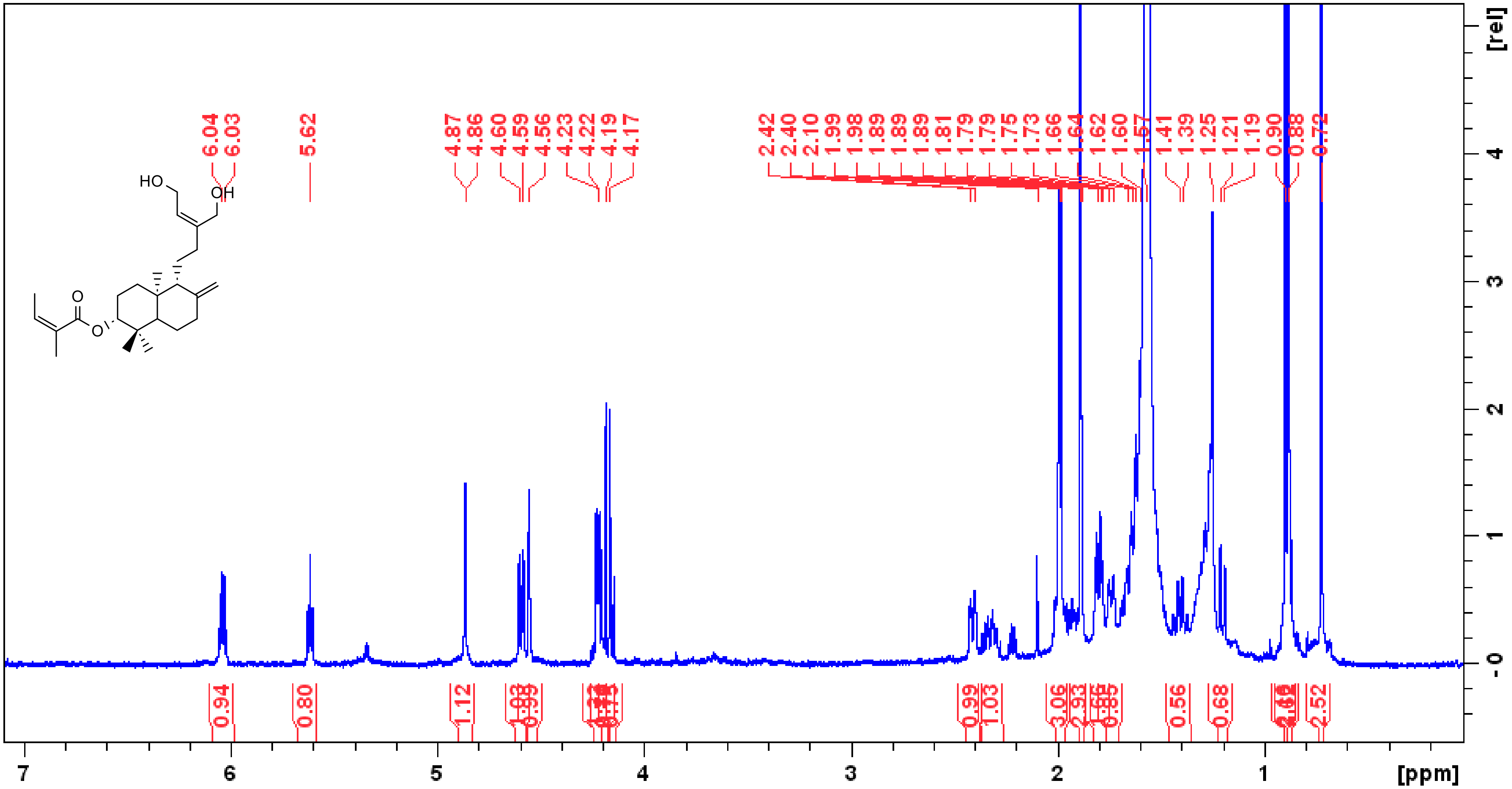

Figure S49. ^1H NMR ($CDCl_3$, 600 MHz) spectrum of **33**.

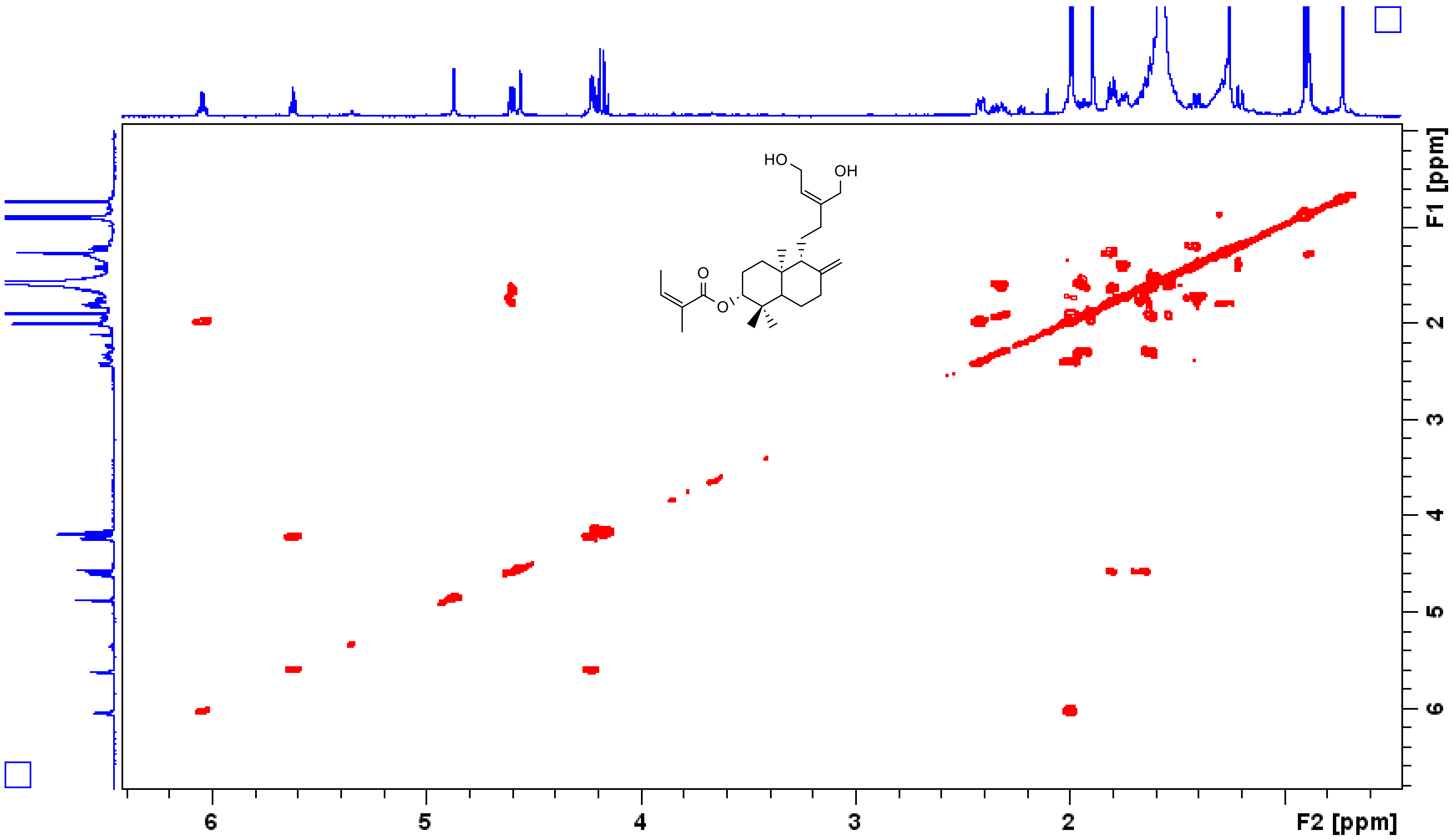

Figure S50. ^1H-^1H COSY NMR (600 MHz, $CDCl_3$) spectrum of **33**.

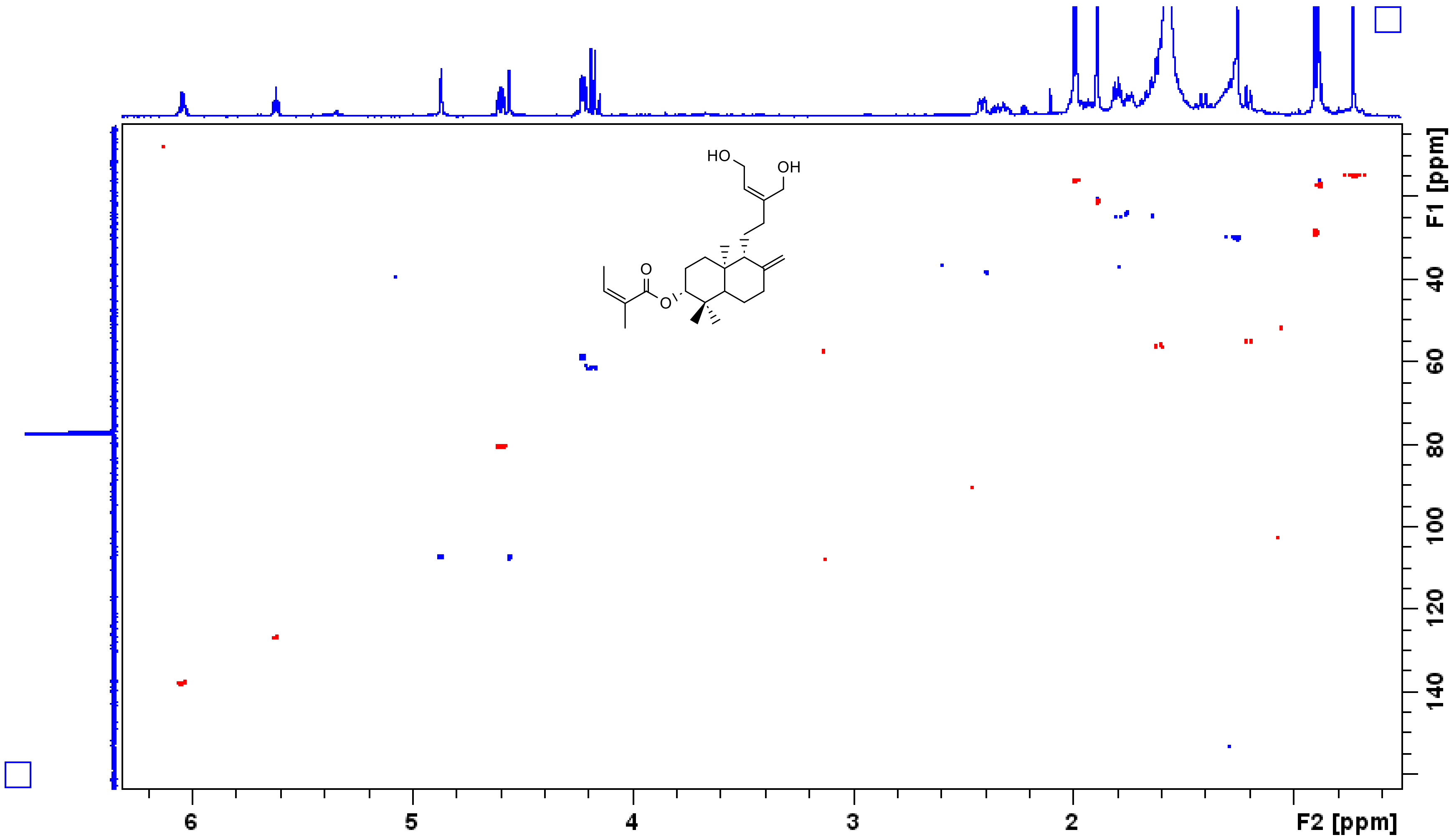

Figure S51. ^{1}H-^{13}C HSQC NMR (600 MHz, $CDCl_3$) spectrum of **33**.

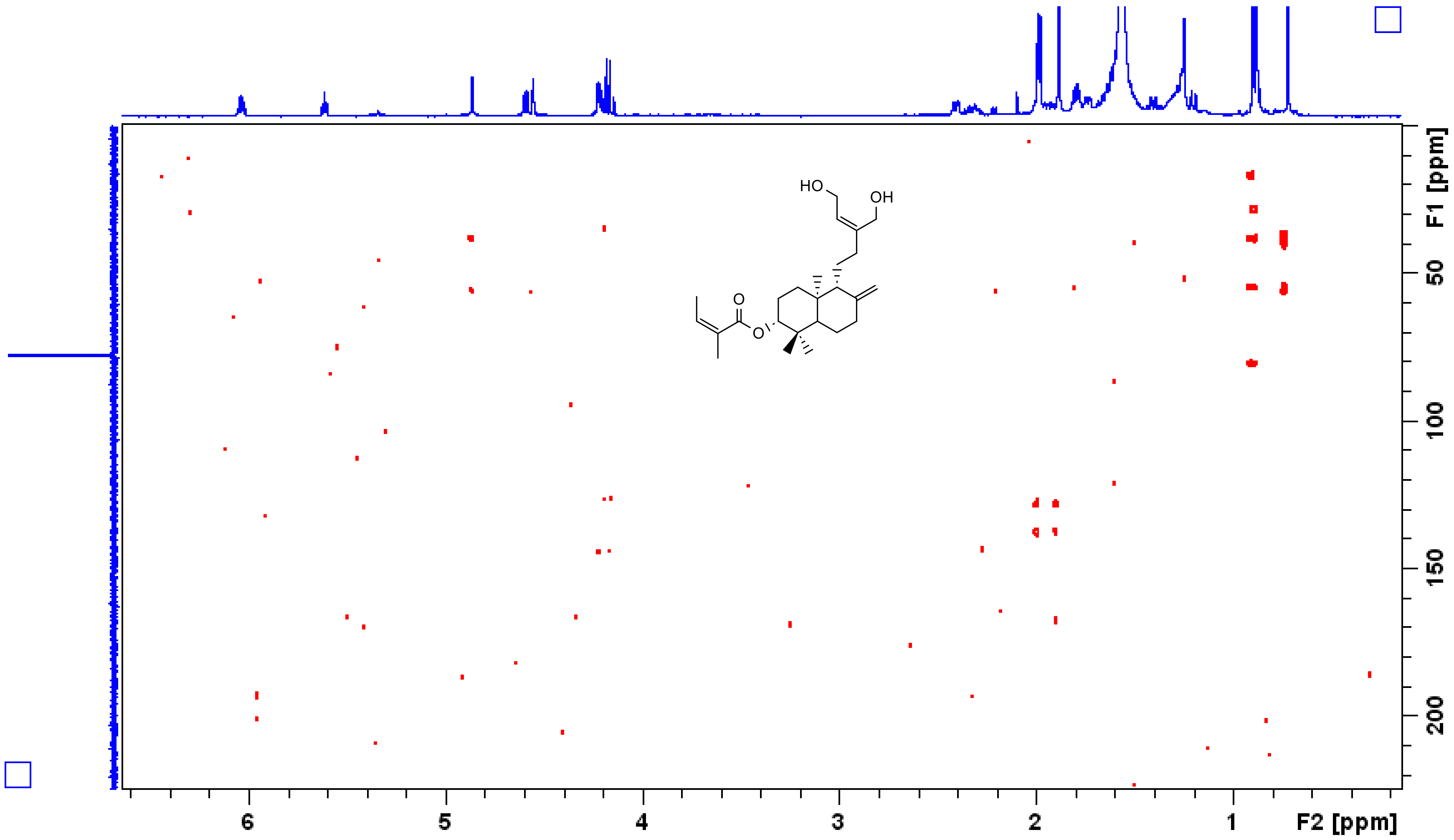

Figure S52. ^{1}H-^{13}C HMBC NMR (600 MHz, $CDCl_3$) spectrum of **33**.

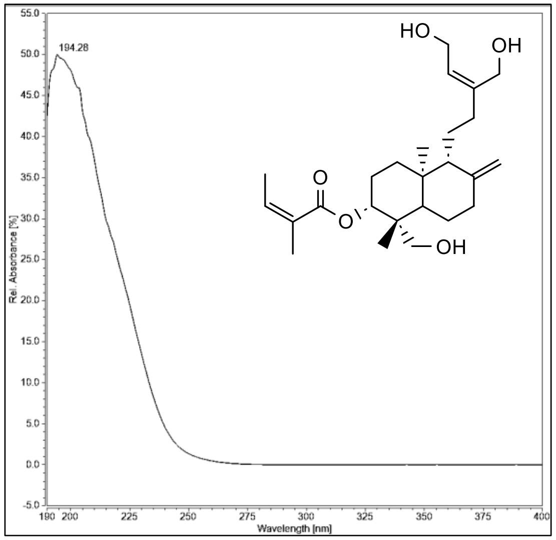

Figure S53. UV spectrum of **34**.

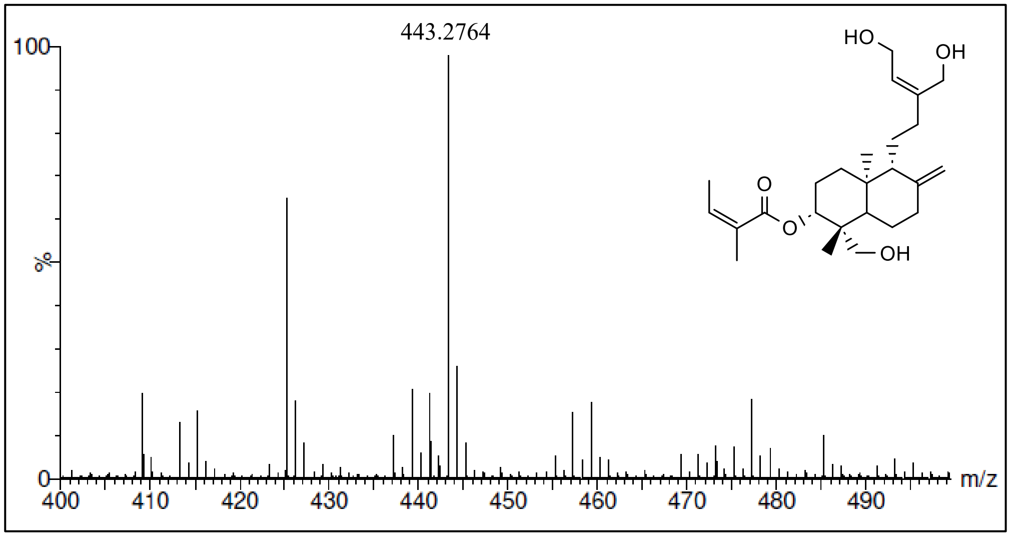

Figure S54. HRESI+MS spectrum of **34**.

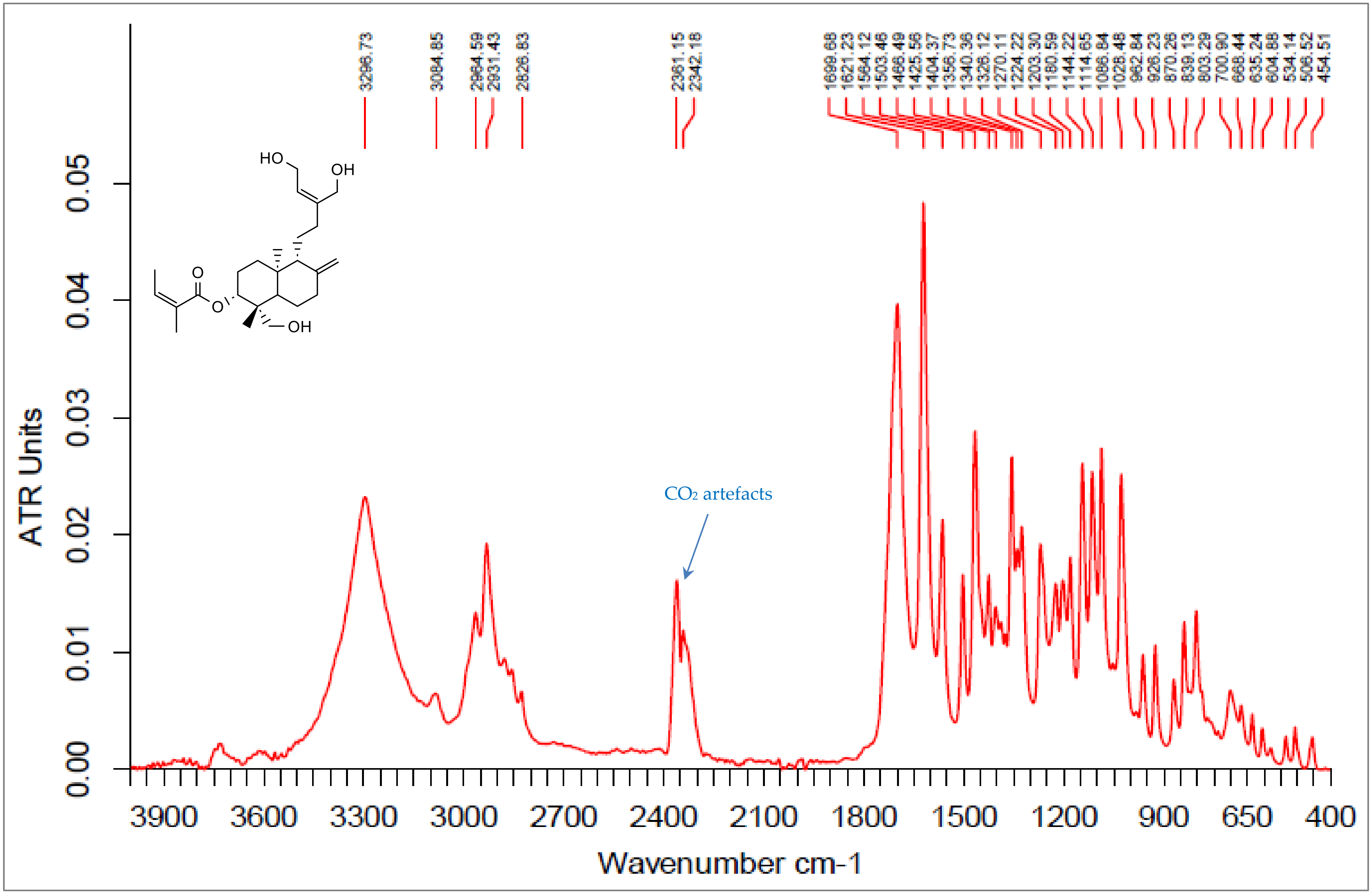

Figure S55. IR (FT-IR) spectrum of **34**.

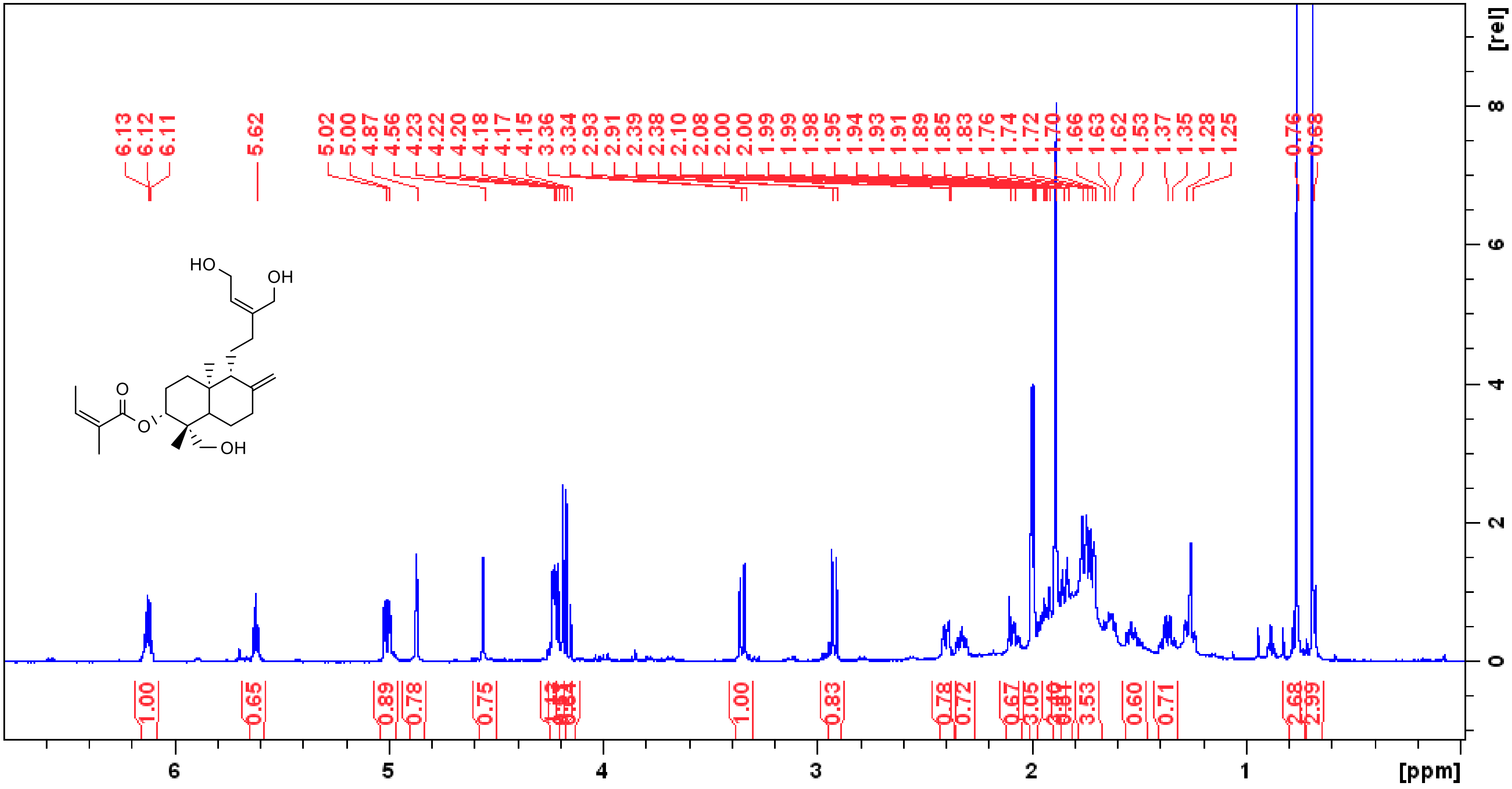

Figure S56. ^{1}H NMR ($CDCl_3$, 600 MHz) spectrum of **34**.

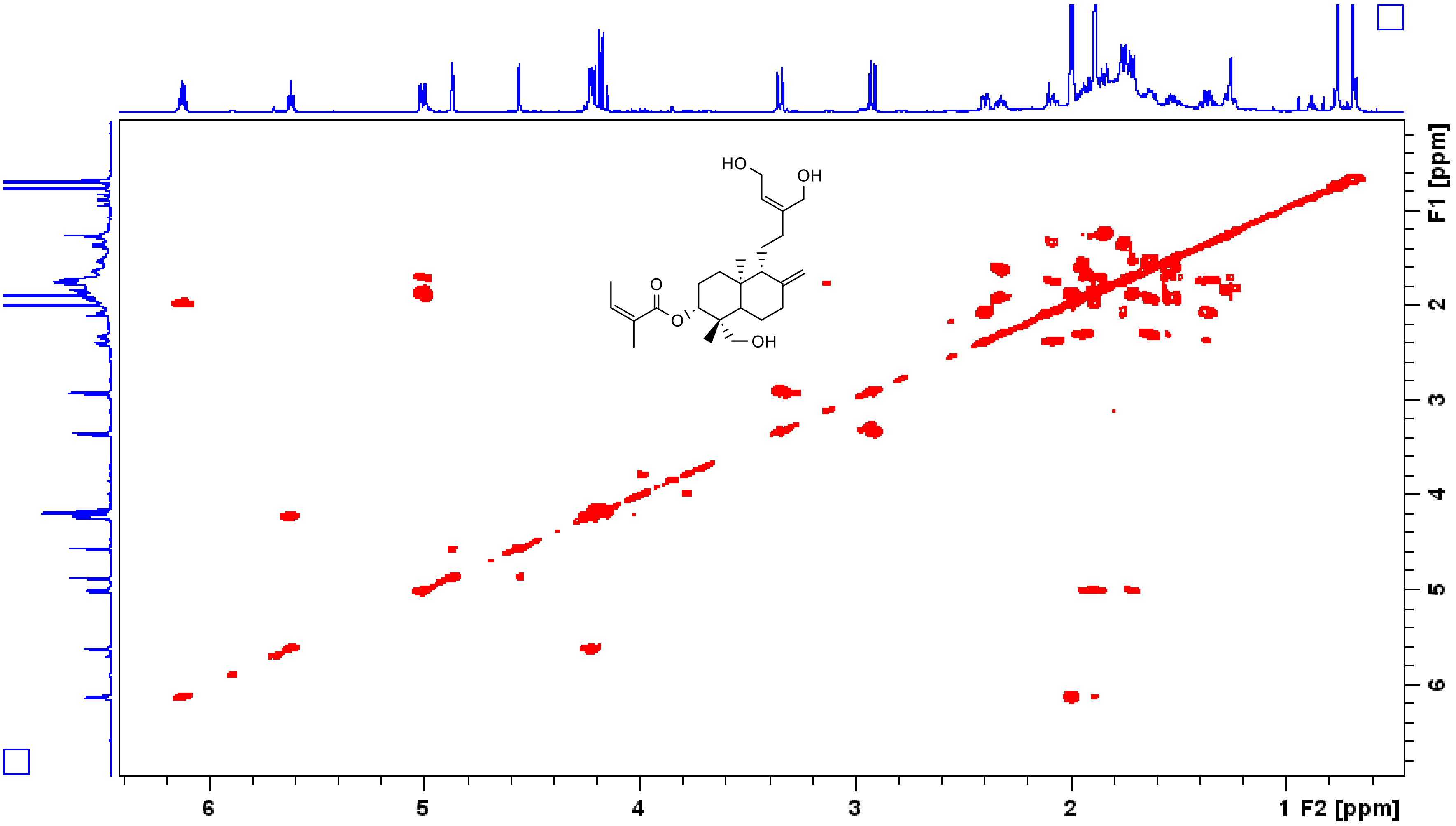

Figure S57. ^{1}H-^{1}H COSY NMR (600 MHz, $CDCl_3$) spectrum of **34**.

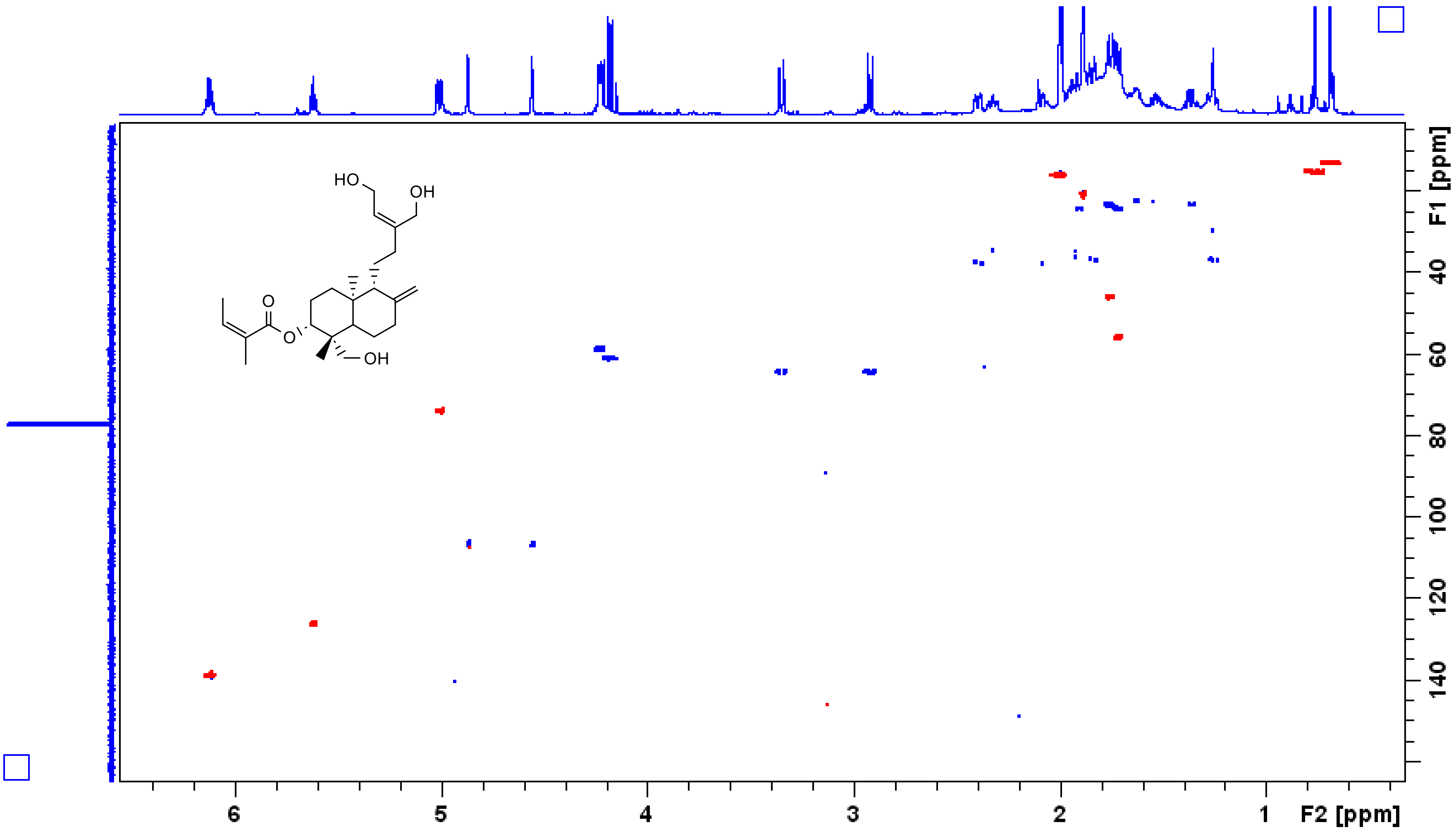

Figure S58. ^{1}H-^{13}C HSQC NMR (600 MHz, $CDCl_3$) spectrum of **34**.

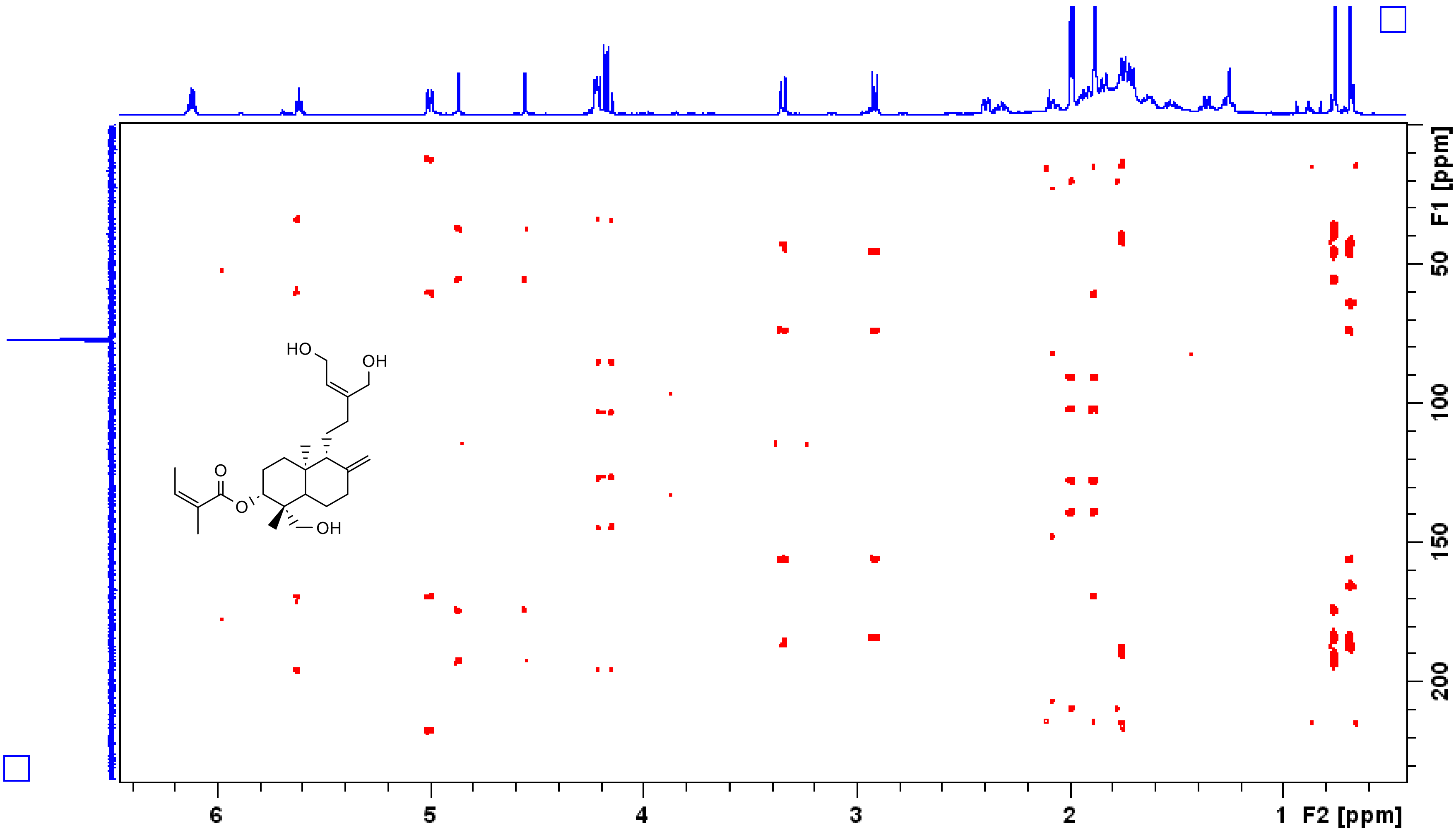

Figure S59. ^{1}H-^{13}C HMBC NMR (600 MHz, $CDCl_3$) spectrum of **34**.

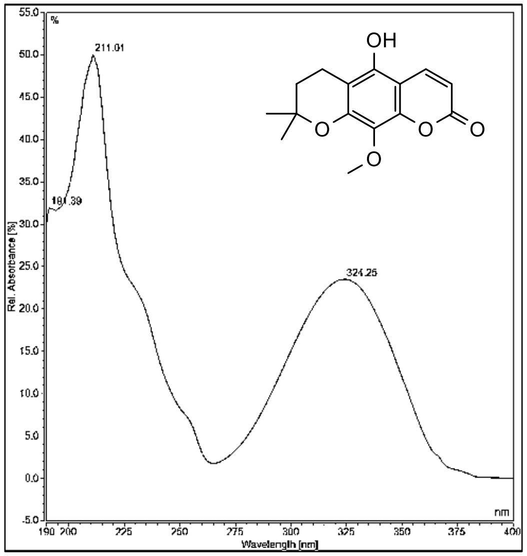

Figure S60. UV spectrum of **35**.

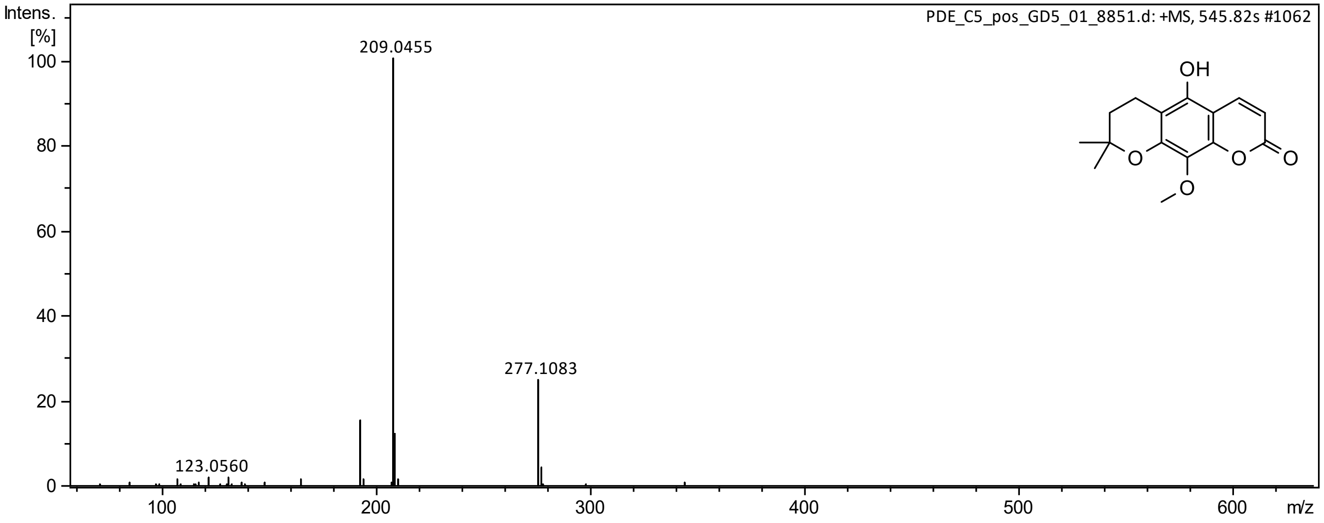

Figure S61. HRESI+MS spectrum of **35**.

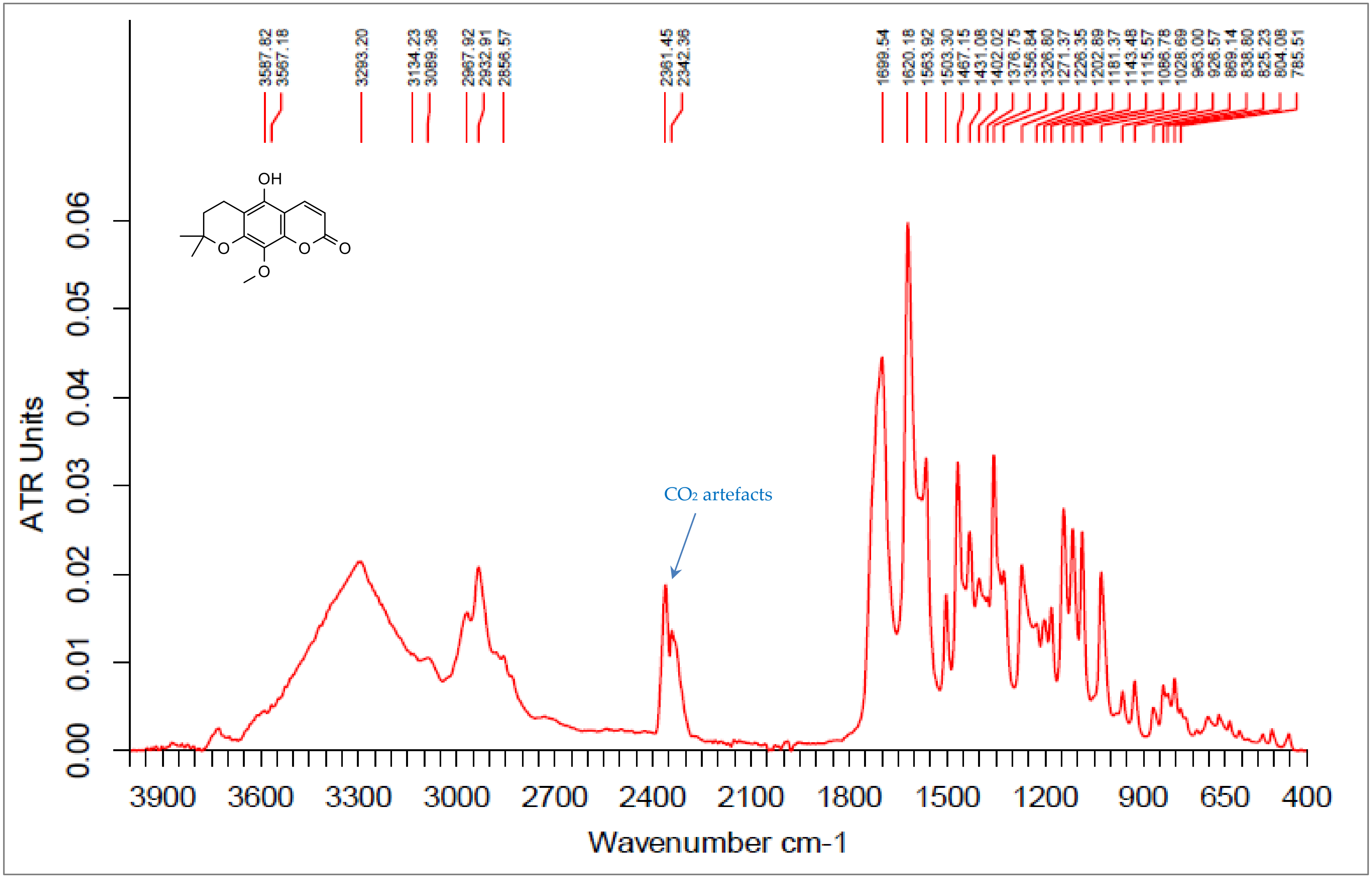

Figure S62. IR (FT-IR) spectrum of **35**.

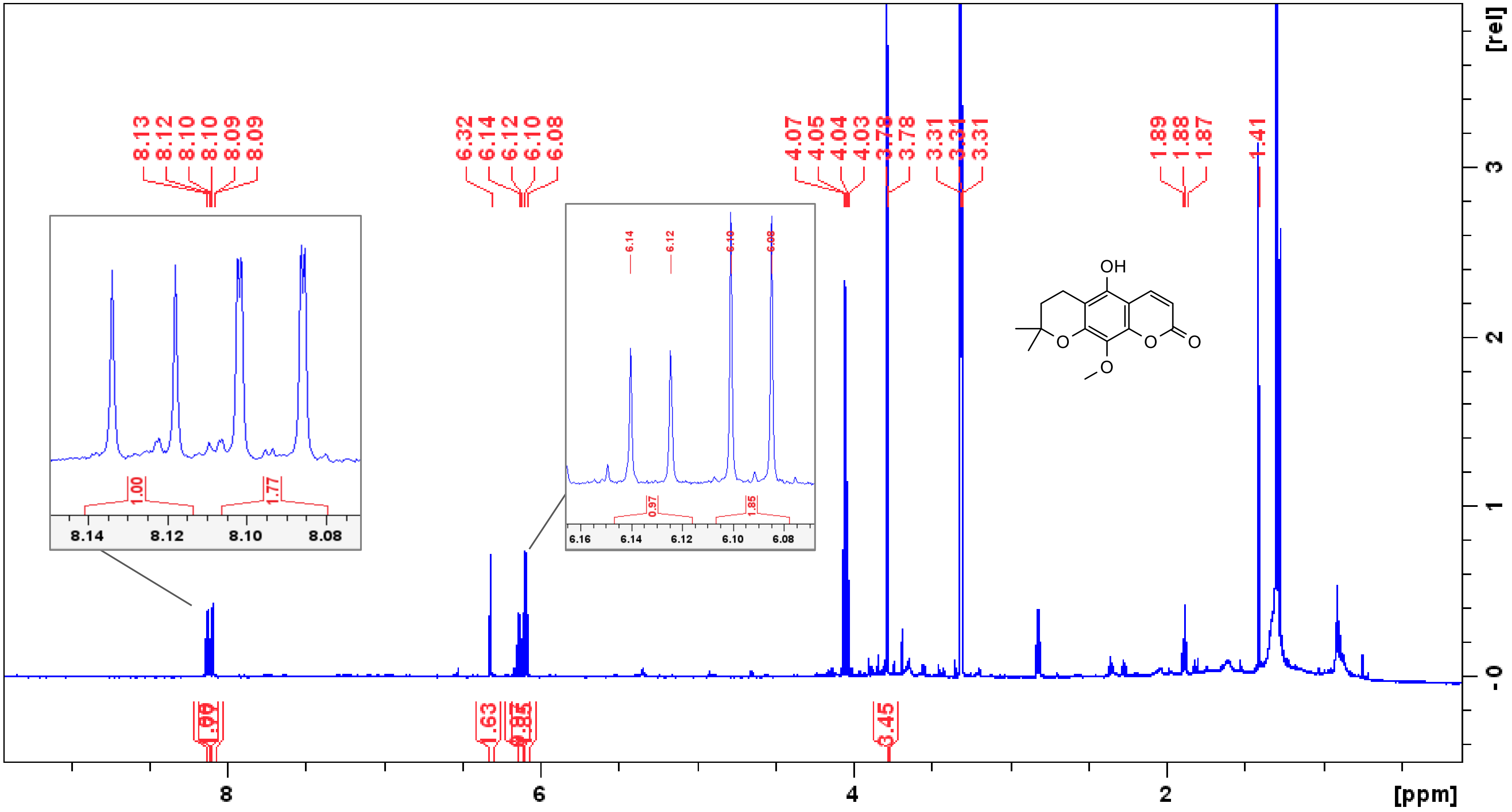

Figure S63. ^{1}H NMR ($CDCl_3$, 600 MHz) spectrum of **35**.

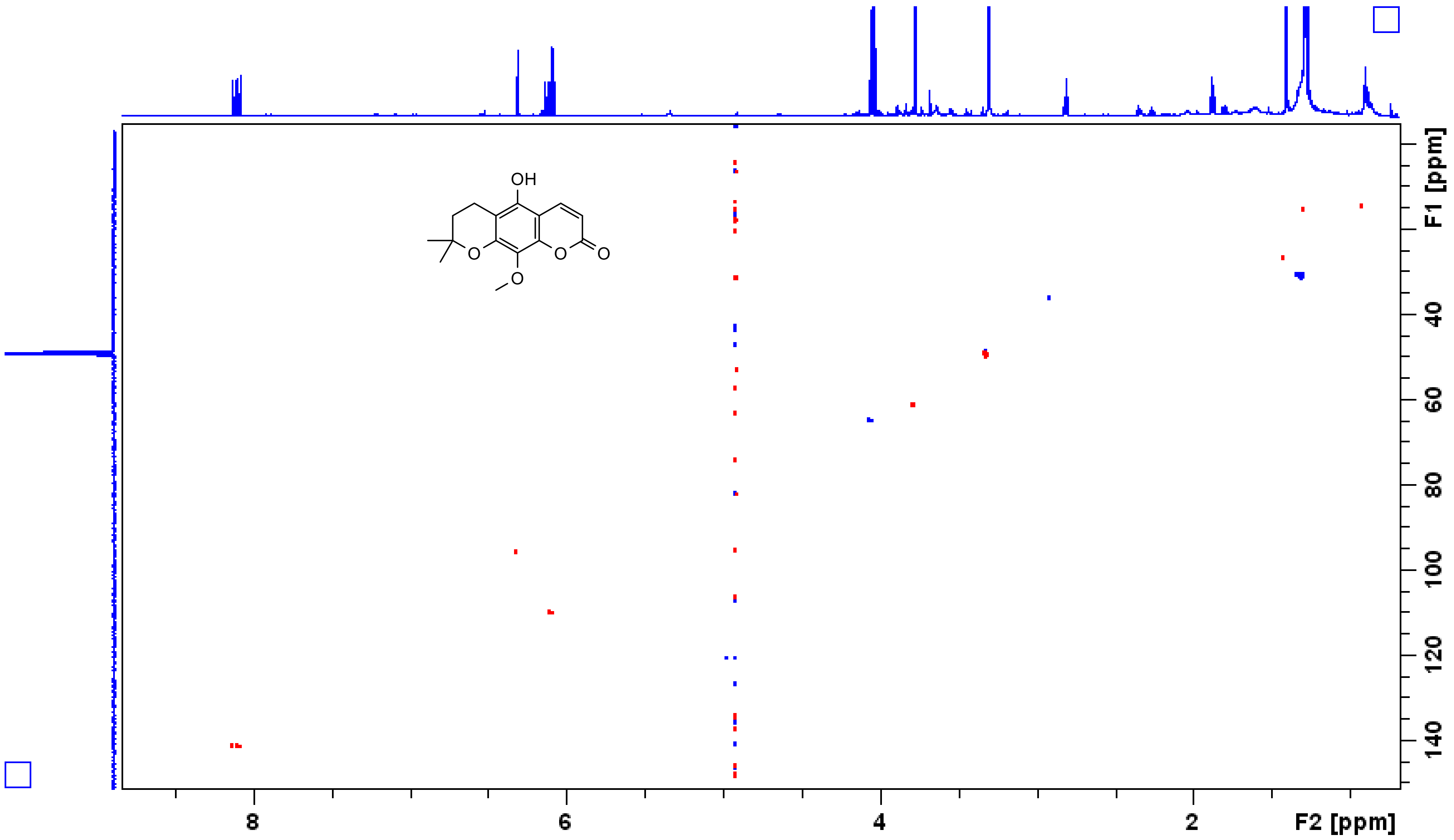

Figure S64. ^{1}H-^{13}C HSQC NMR (600 MHz, $CDCl_3$) spectrum of **35**.

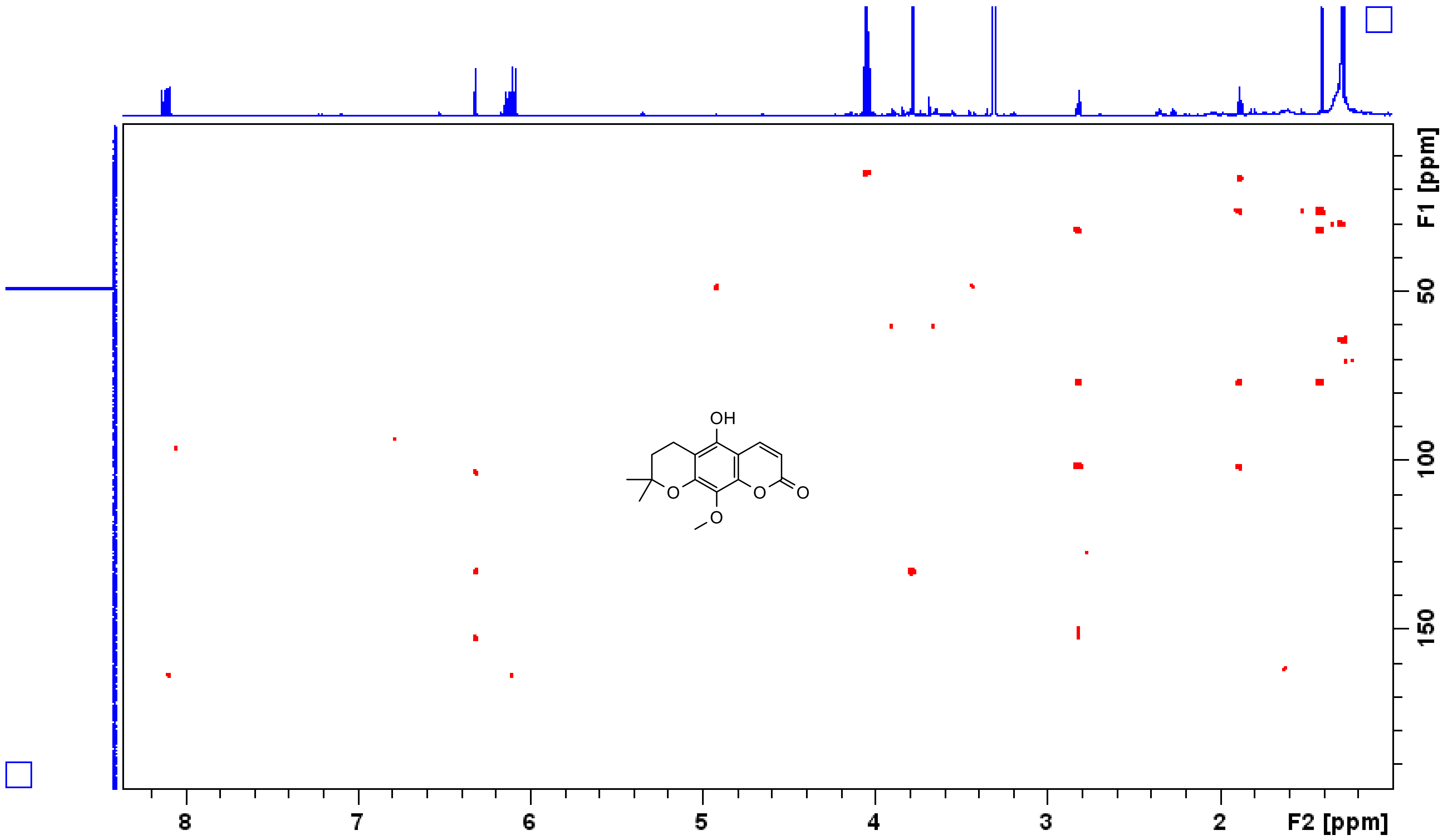

Figure S65. ^{1}H-^{13}C HMBC NMR (600 MHz, $CDCl_3$) spectrum of **35**.

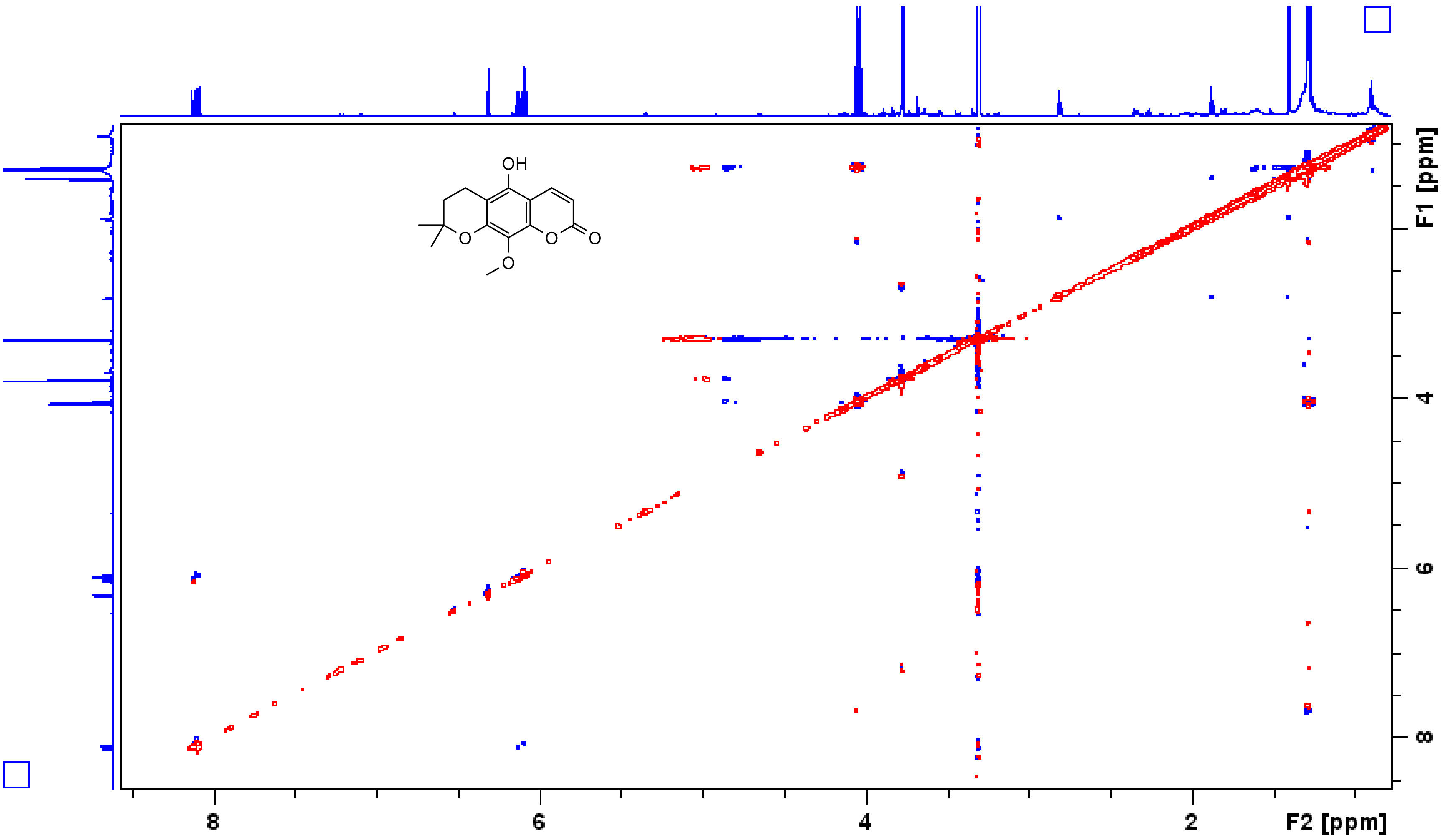

Figure S66. ^{1}H-^{1}H NOESY NMR (600 MHz, $CDCl_3$) spectrum of **35**.

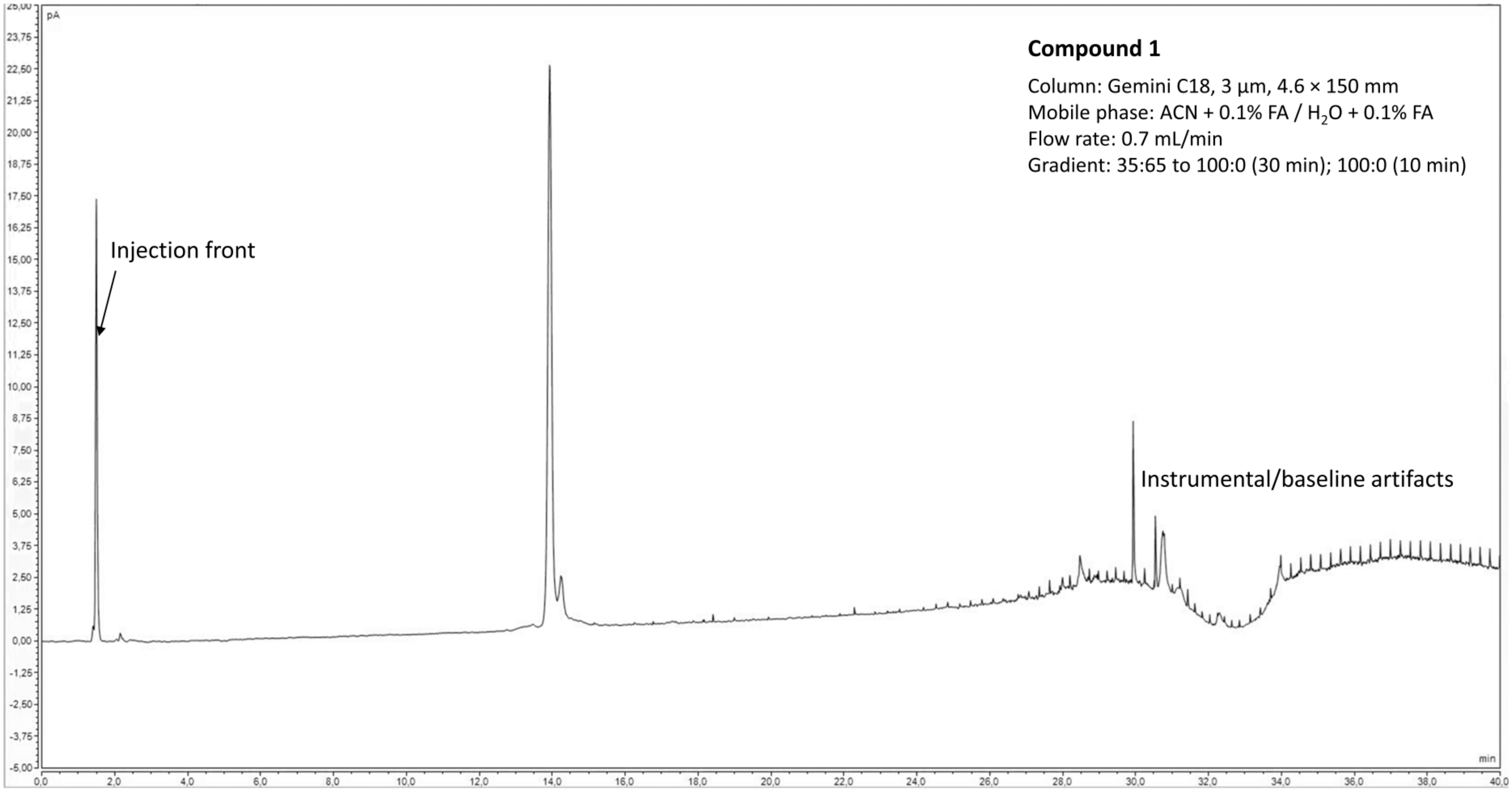

Figure S67. HPLC-CAD chromatogram of compound **1**.

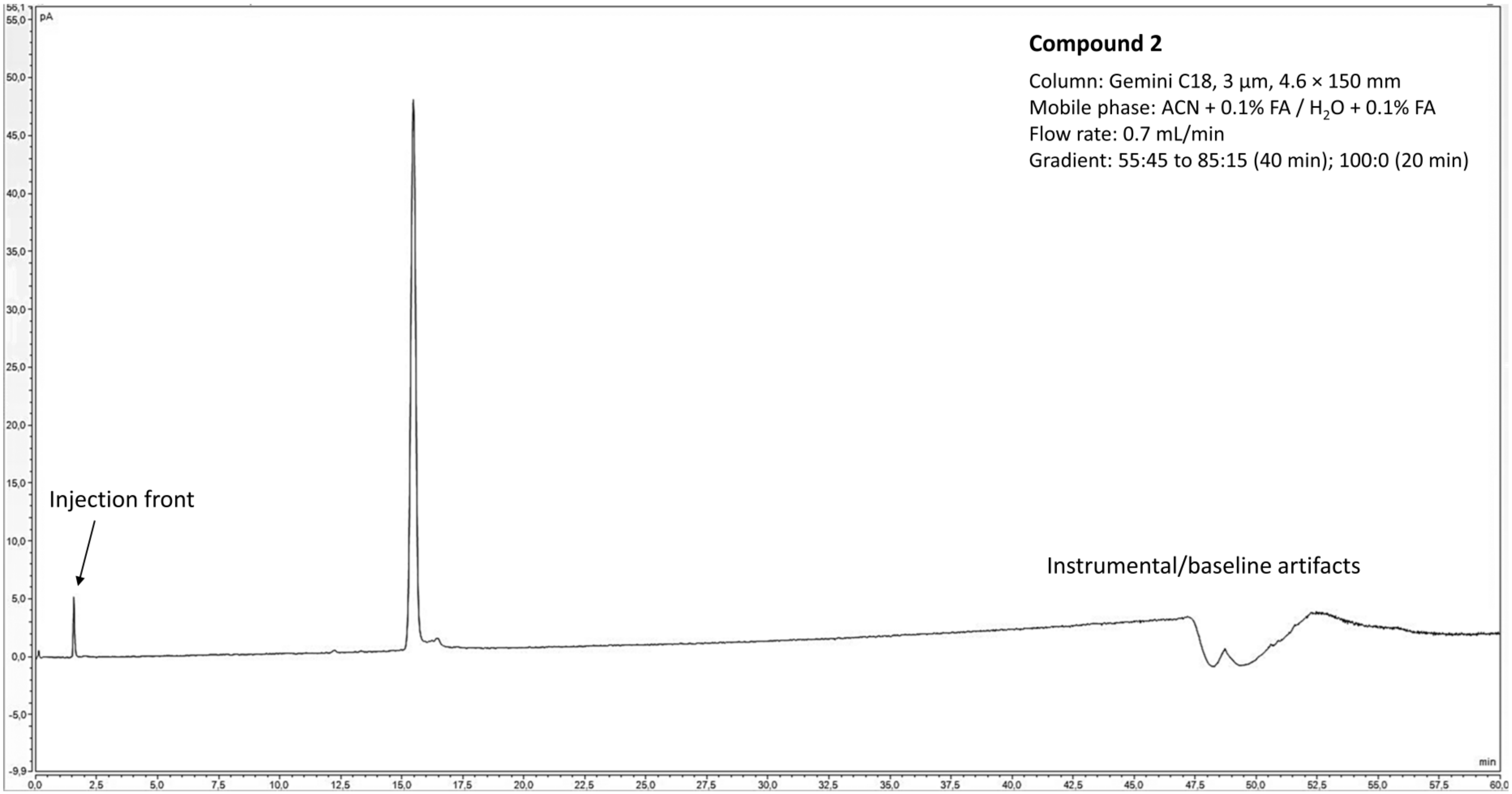

Figure S68. HPLC-CAD chromatogram of compound **2**.

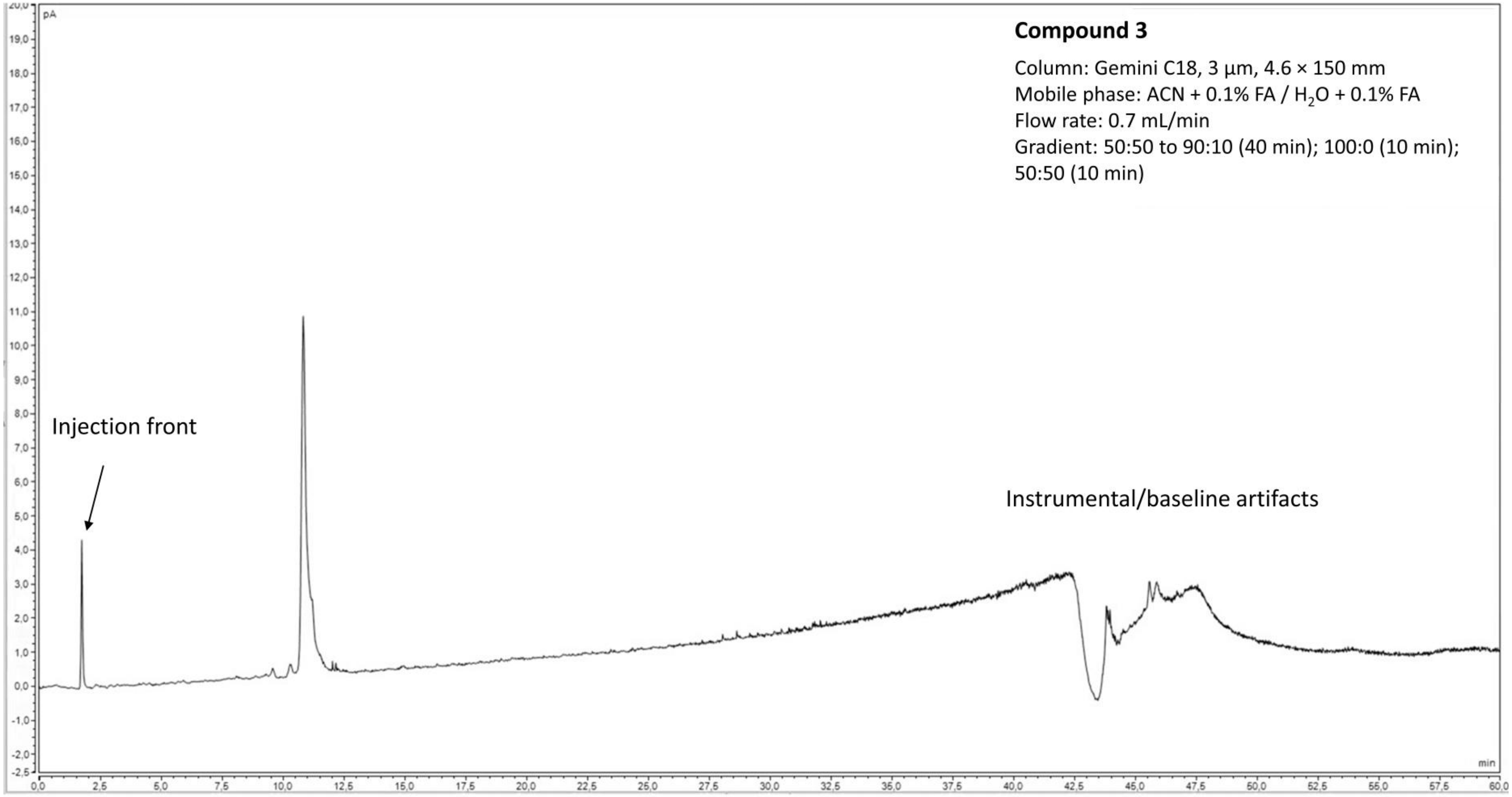

Figure S69. HPLC-CAD chromatogram of compound **3**.

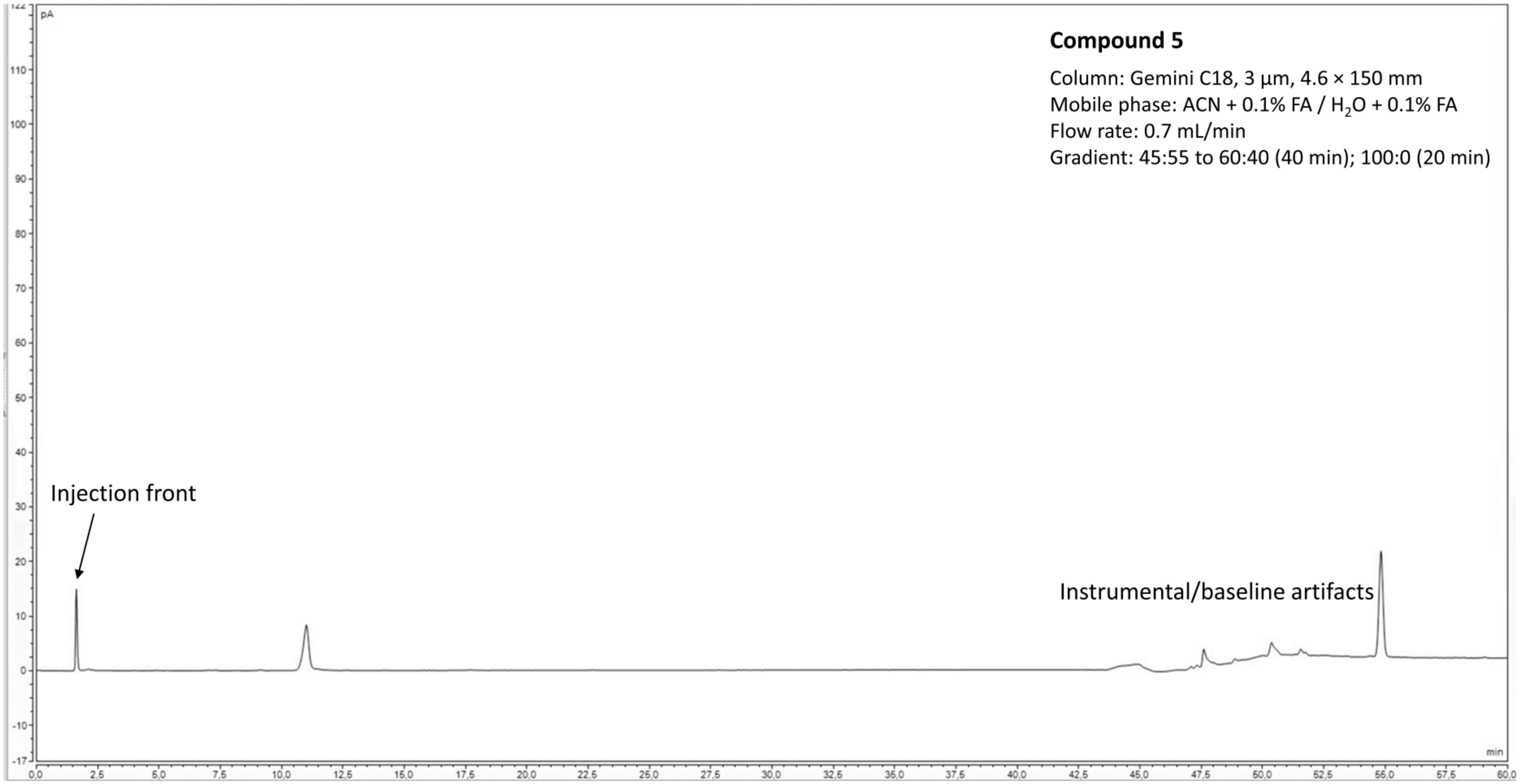

Figure S70. HPLC-CAD chromatogram of compound **5**.

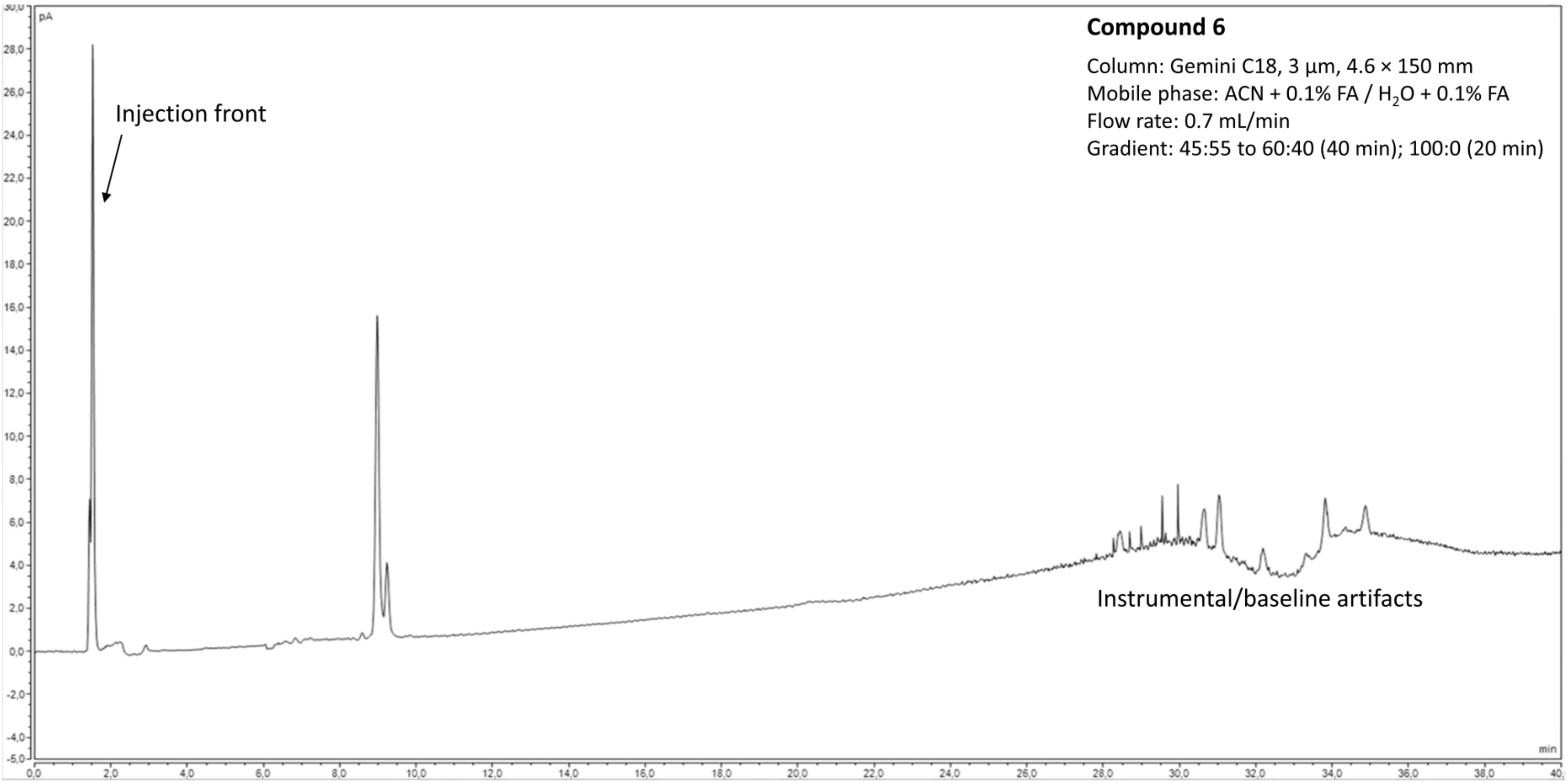

Figure S71. HPLC-CAD chromatogram of compound **6**.

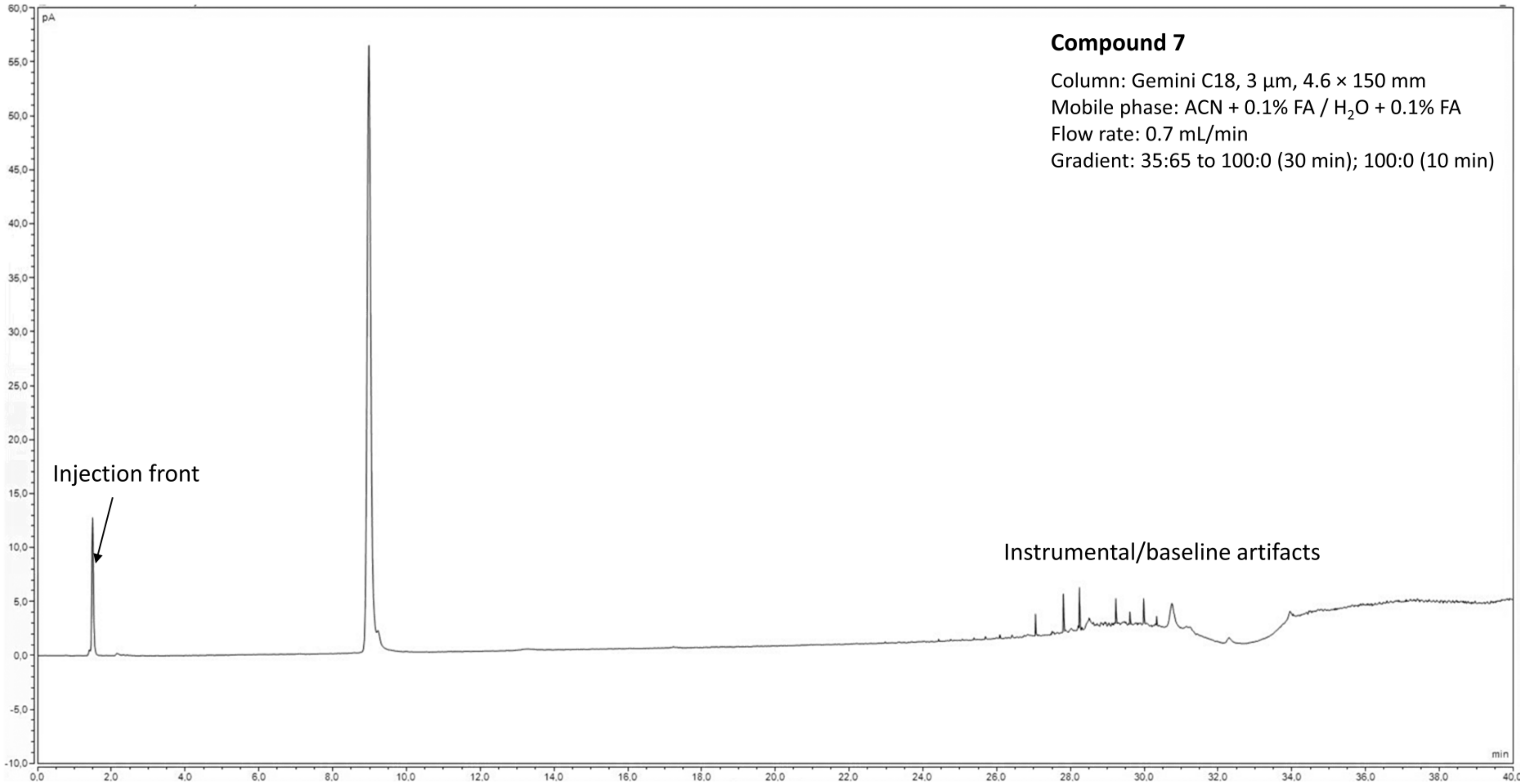

Figure S72. HPLC-CAD chromatogram of compound **7**.

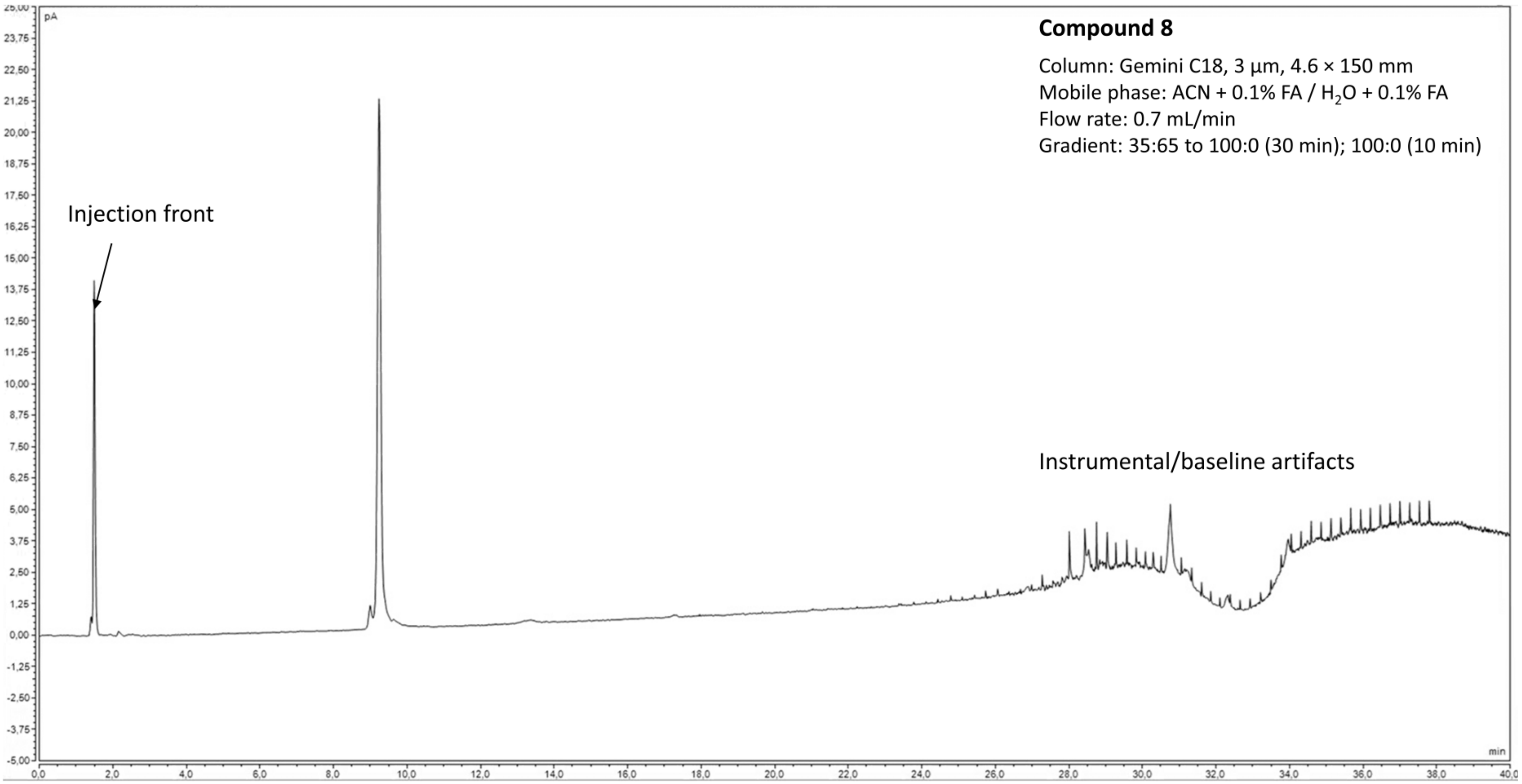

Figure S73. HPLC-CAD chromatogram of compound **8**.

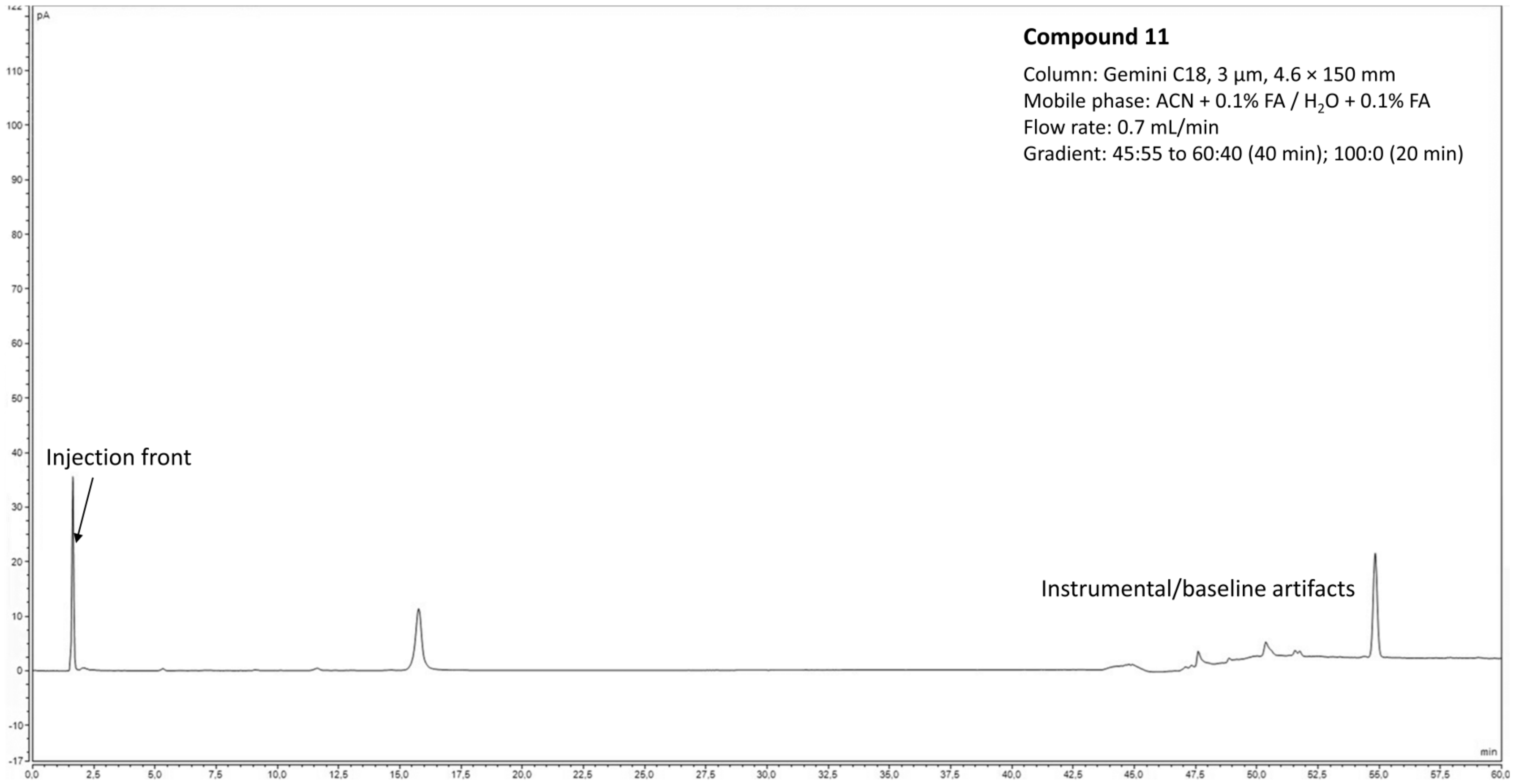

Figure S74. HPLC-CAD chromatogram of compound **11**.

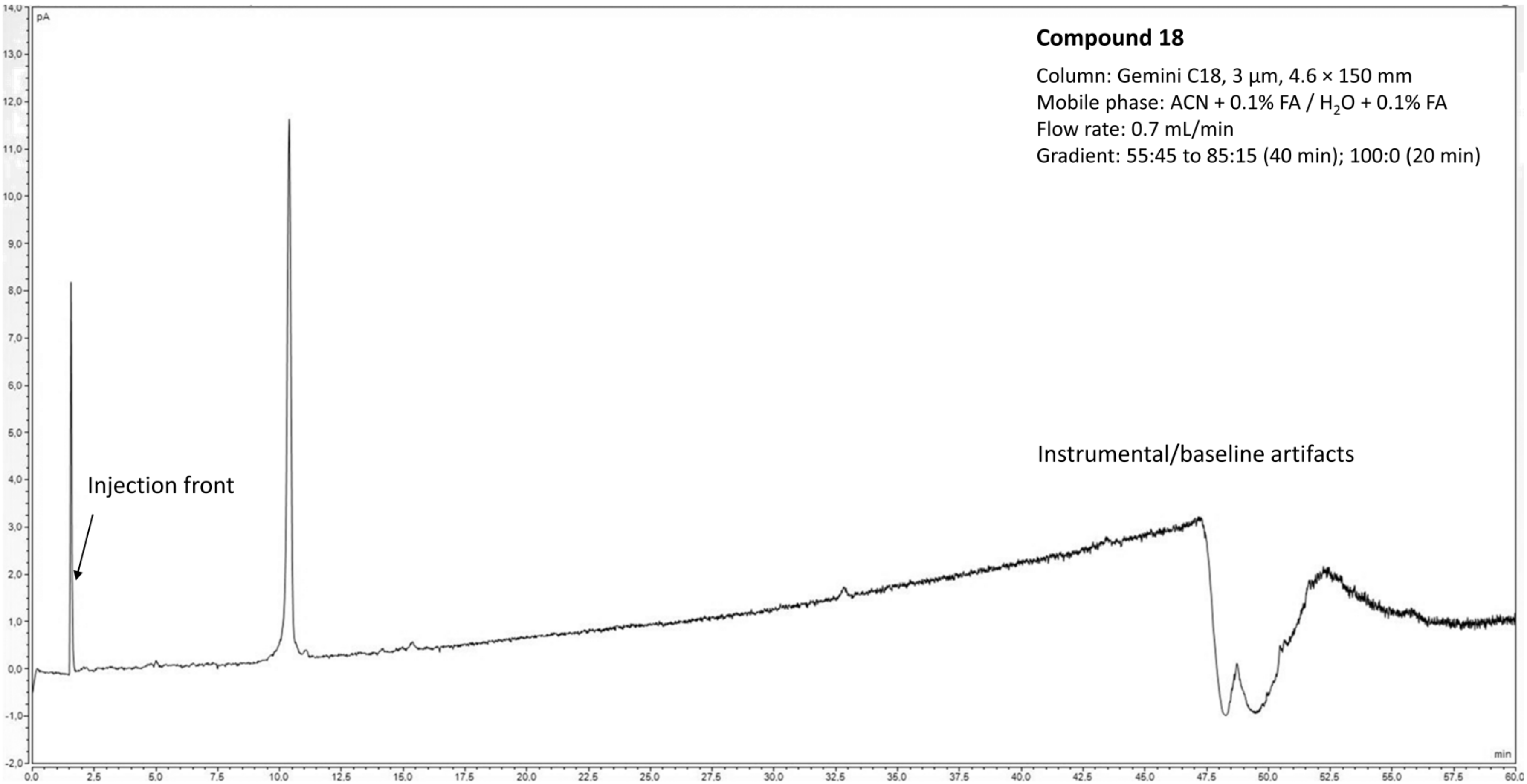

Figure S75. HPLC-CAD chromatogram of compound **18**.

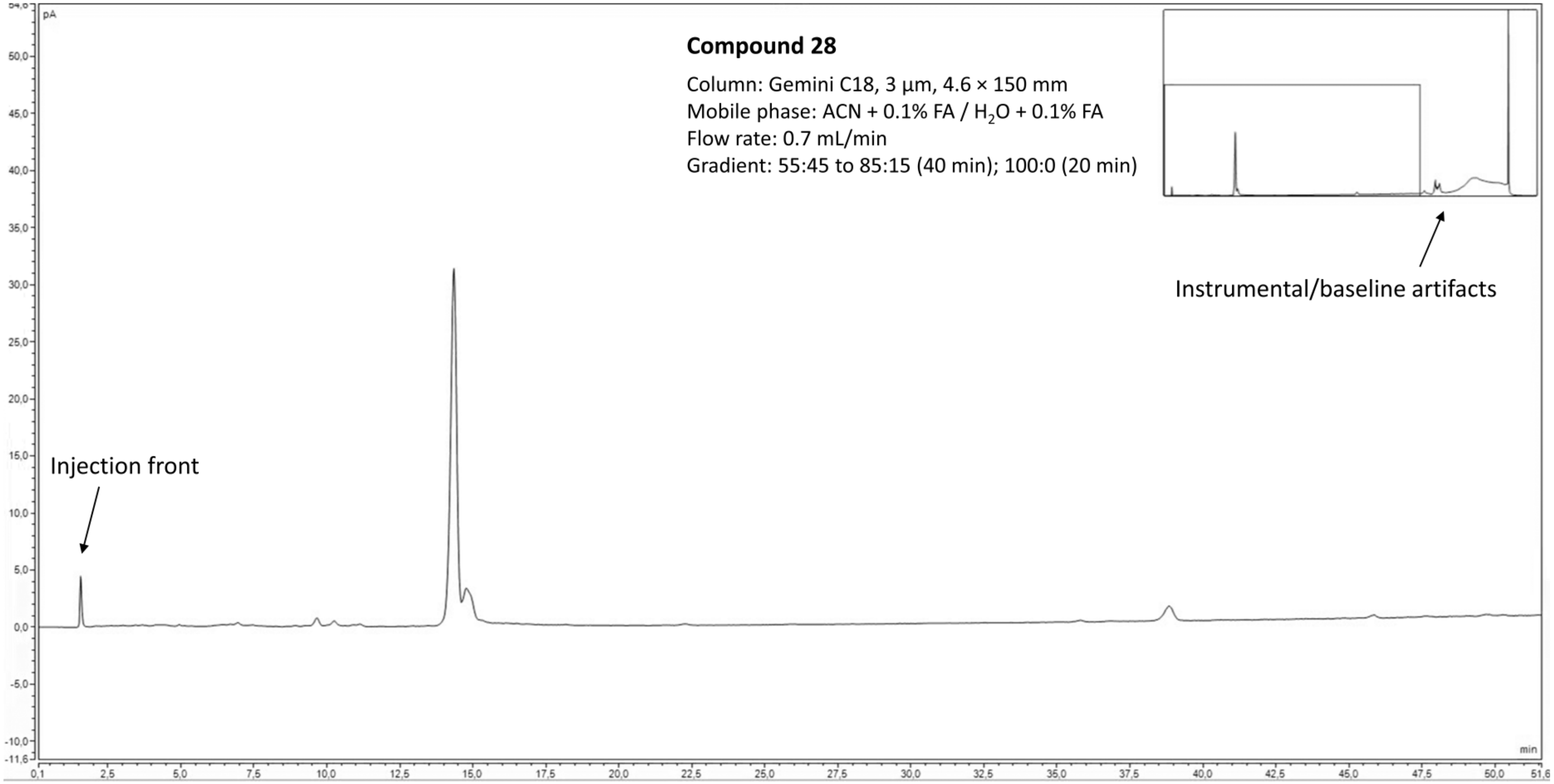

Figure S76. HPLC-CAD chromatogram of compound **28**.

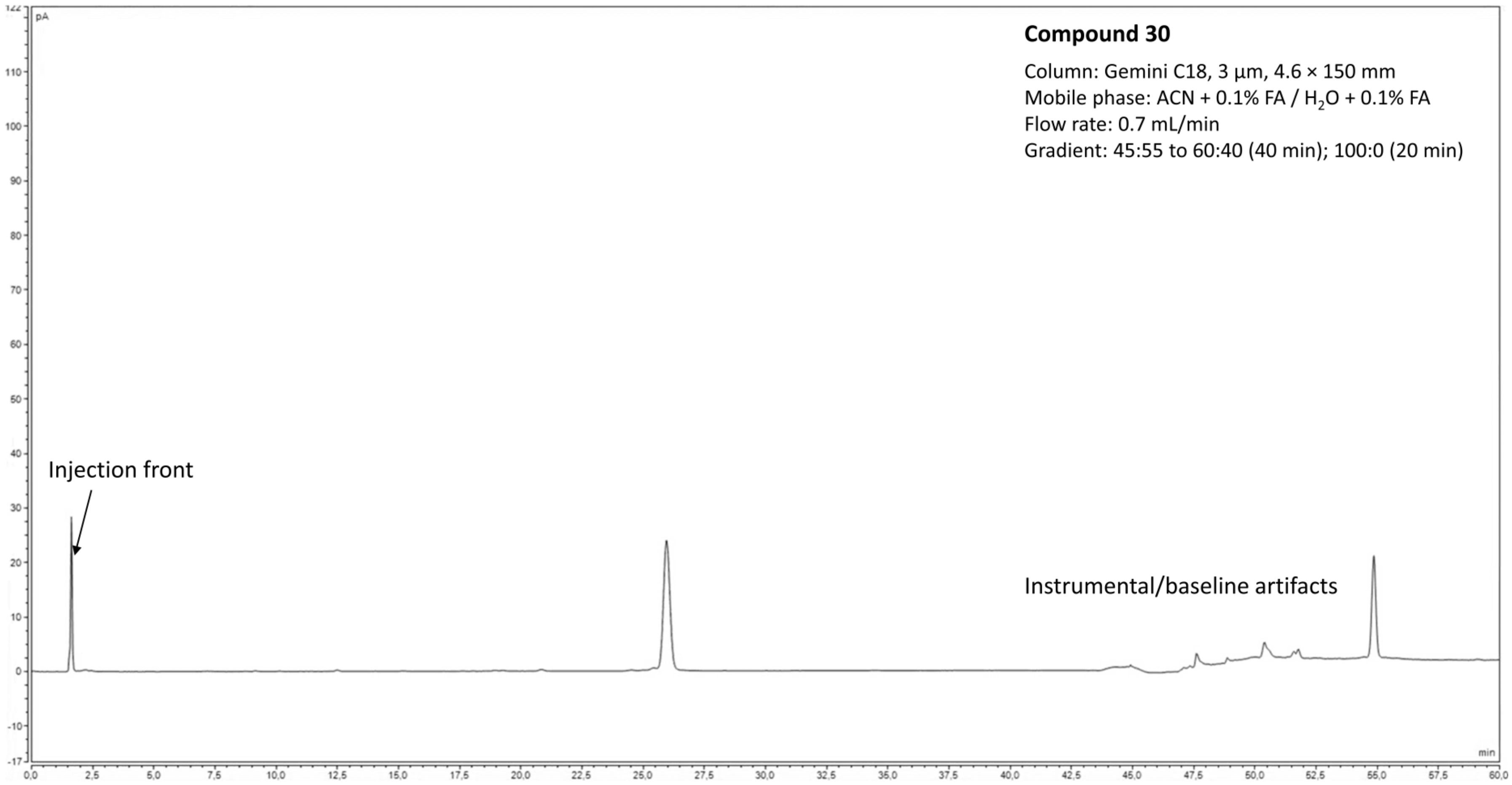

Figure S77. HPLC-CAD chromatogram of compound **30**.

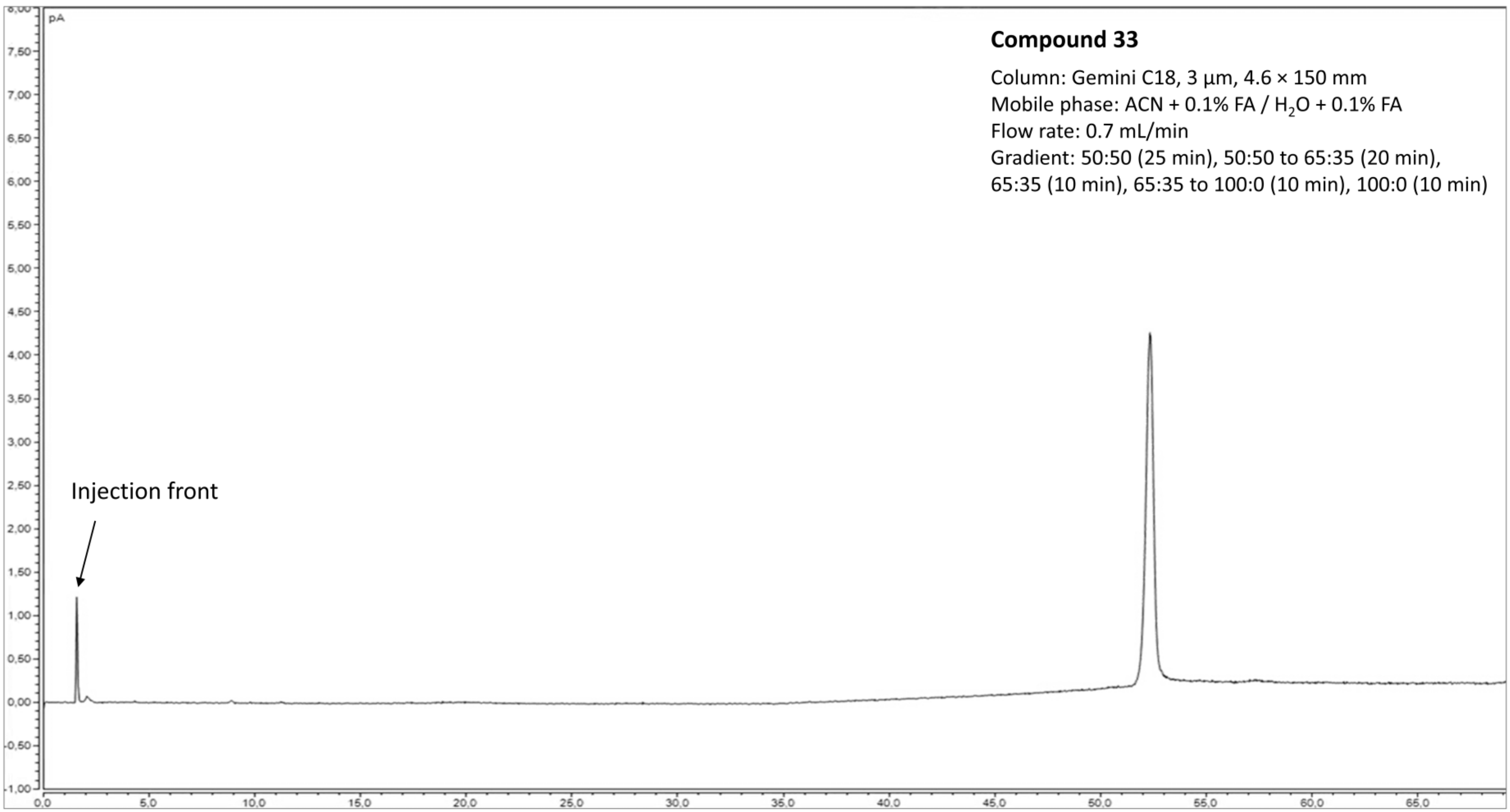

Figure S78. HPLC-CAD chromatogram of compound **33**.

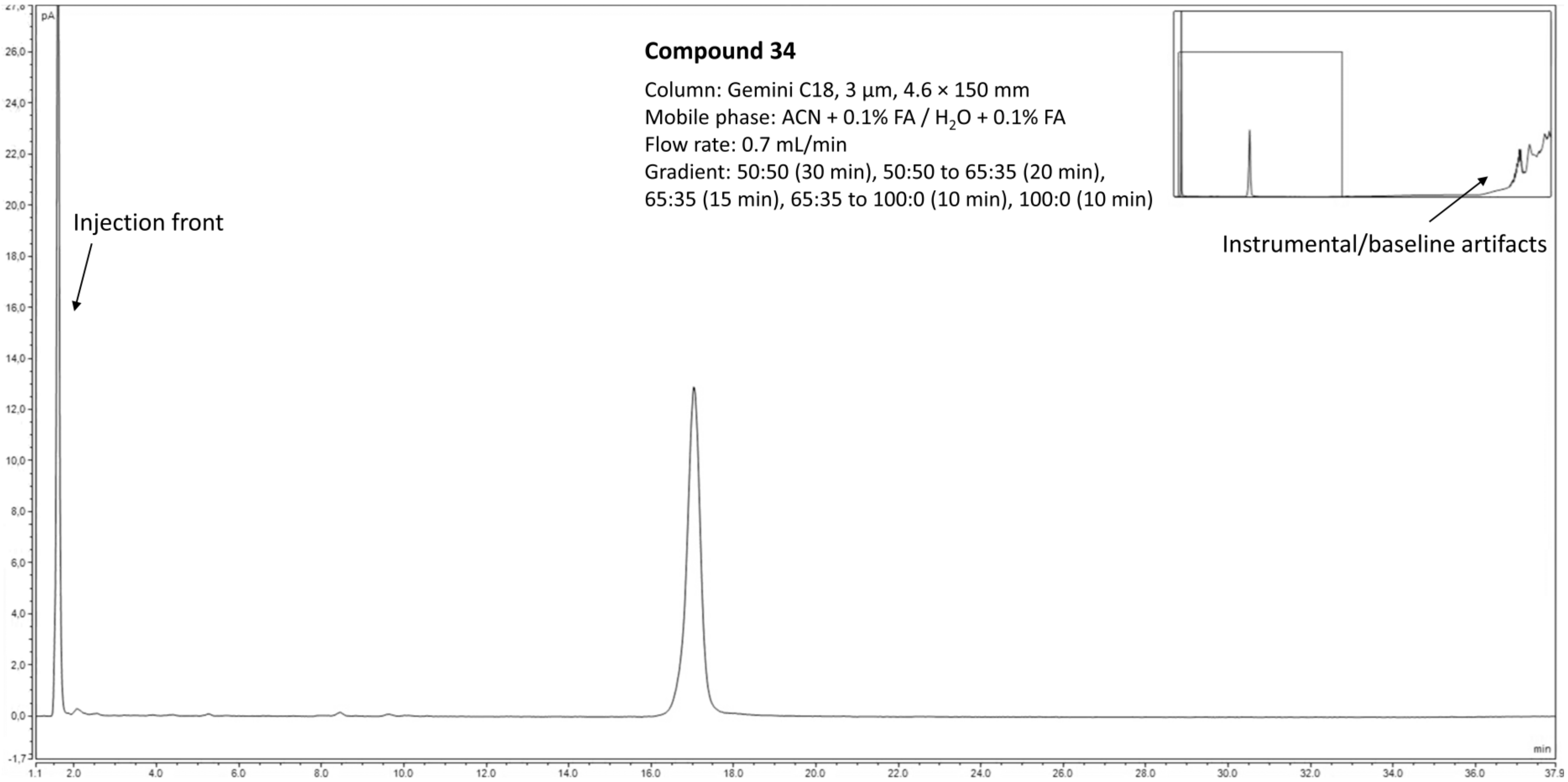

Figure S79. HPLC-CAD chromatogram of compound **34**.

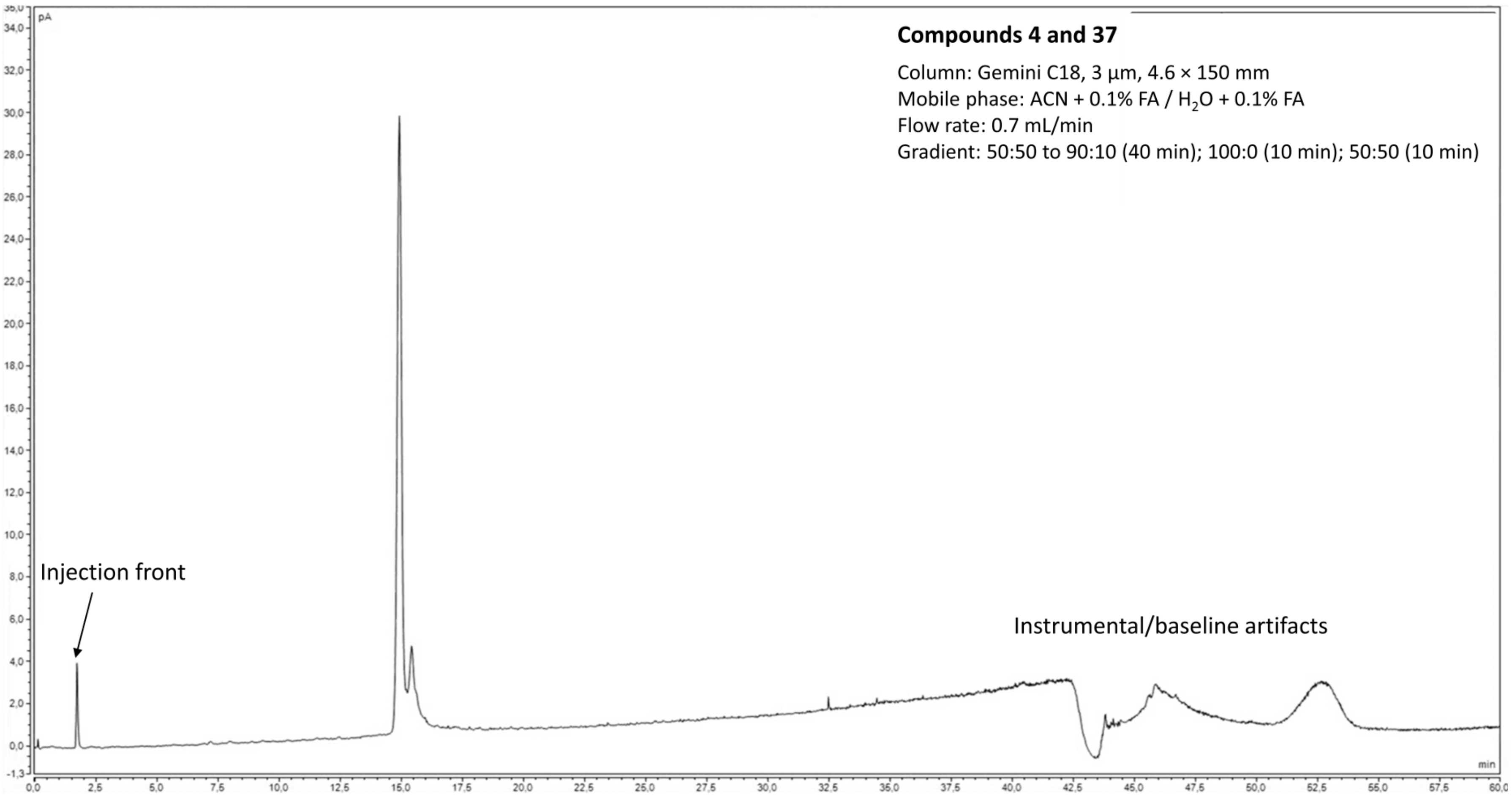

Figure S80. HPLC-CAD chromatogram of compounds **4** and **37**.

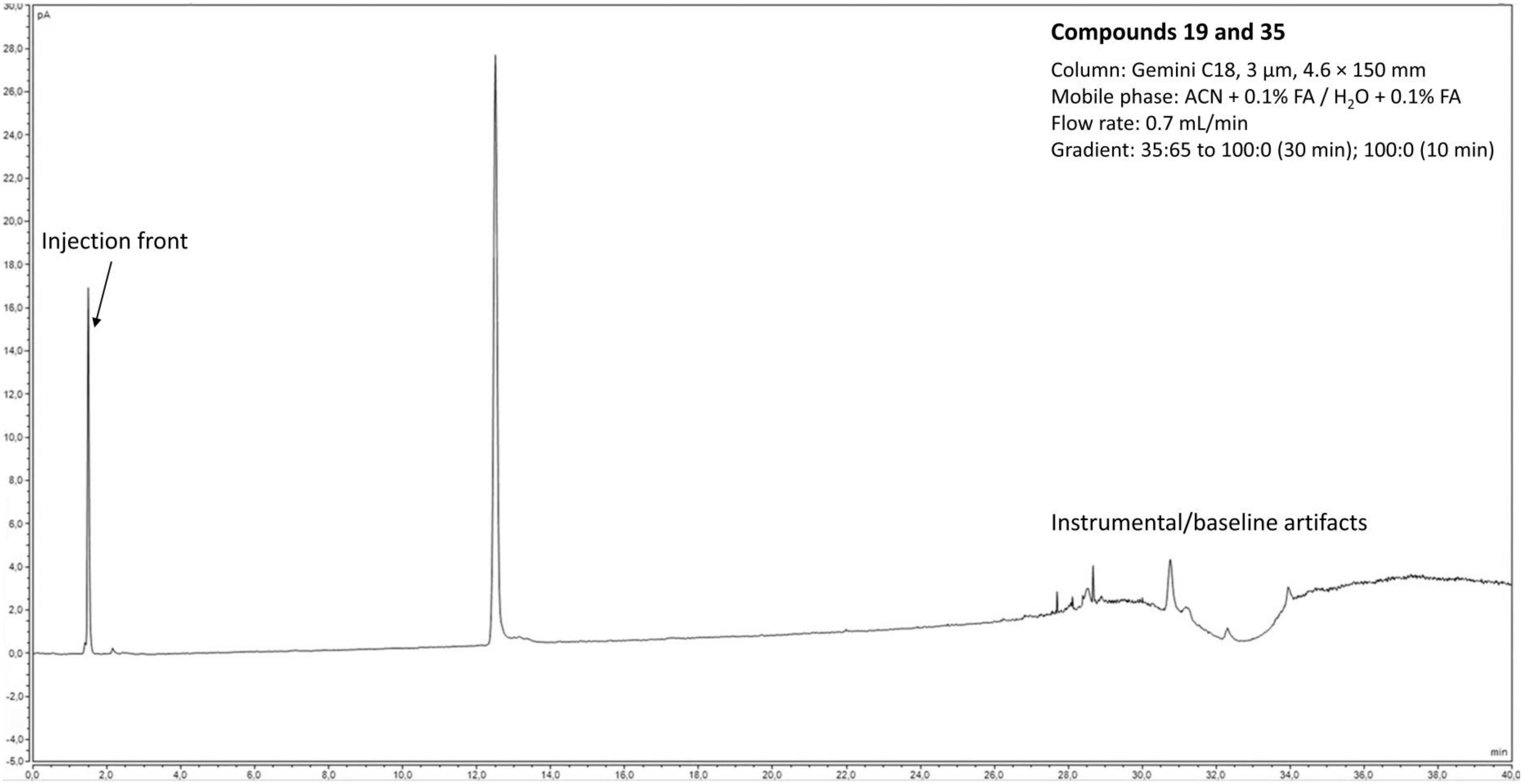

Figure S81. HPLC-CAD chromatogram of compounds **19** and **34**.

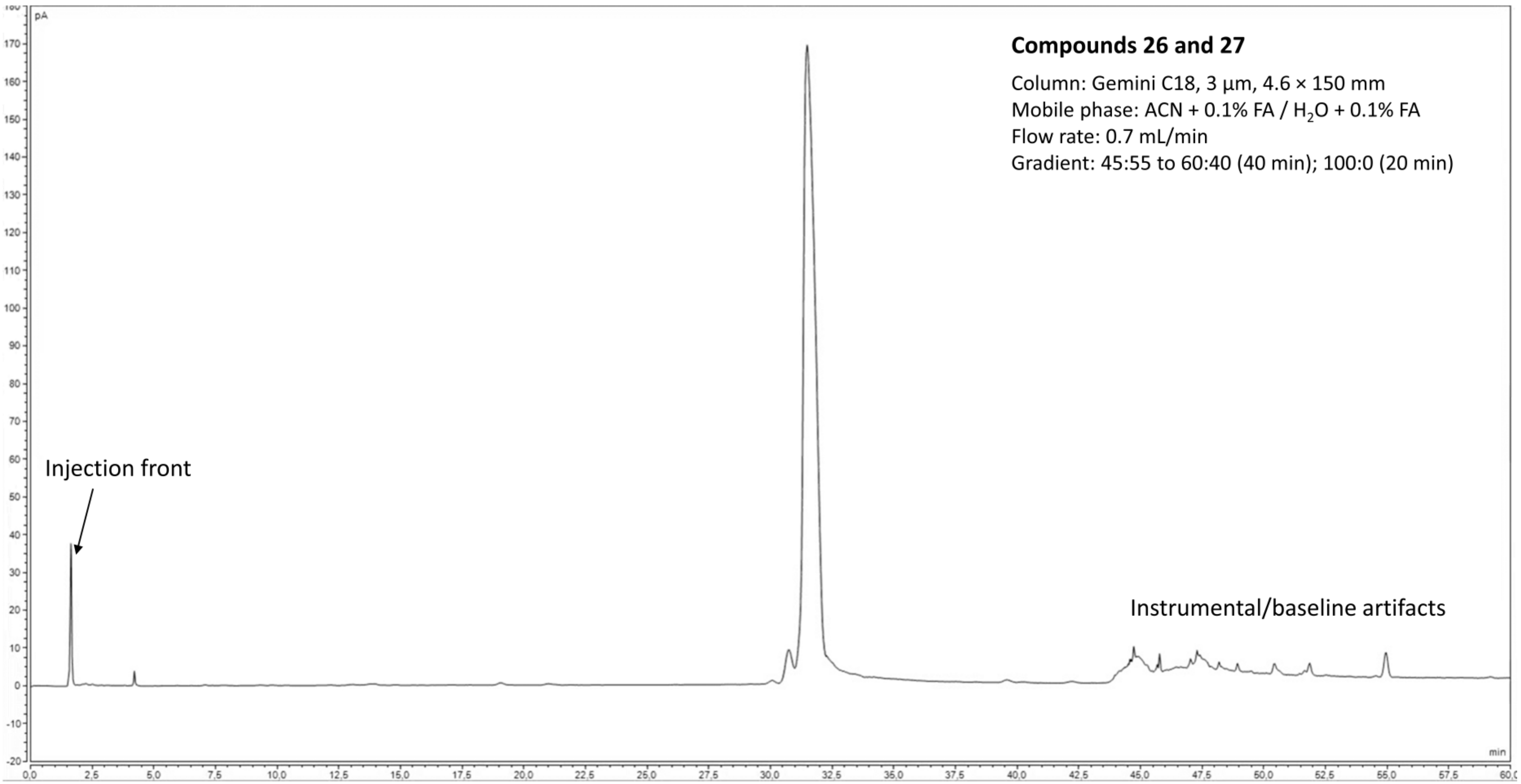

Figure S82. HPLC-CAD chromatogram of compounds **26** and **27**.

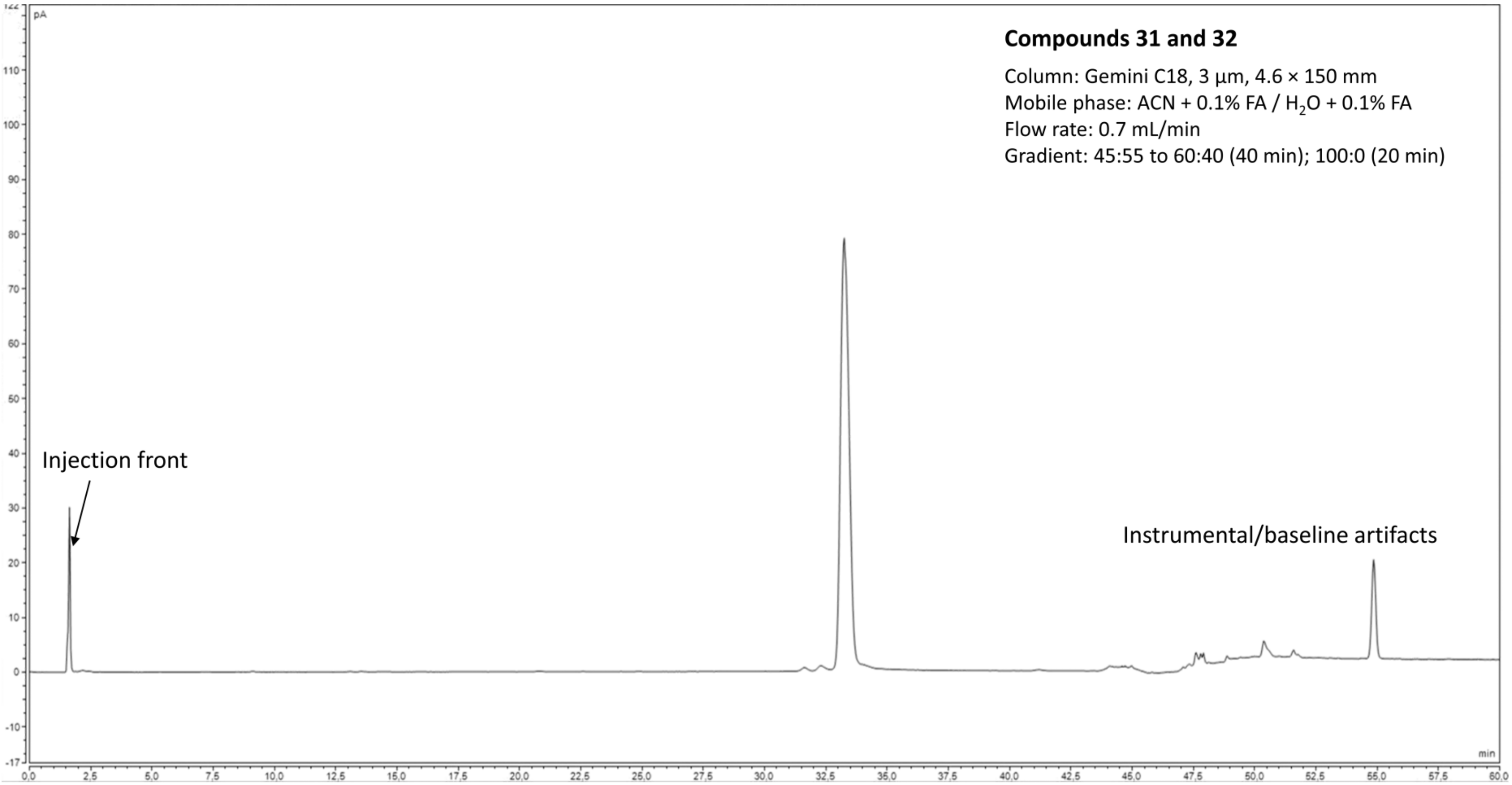

Figure S83. HPLC-CAD chromatogram of compounds **31** and **32**.

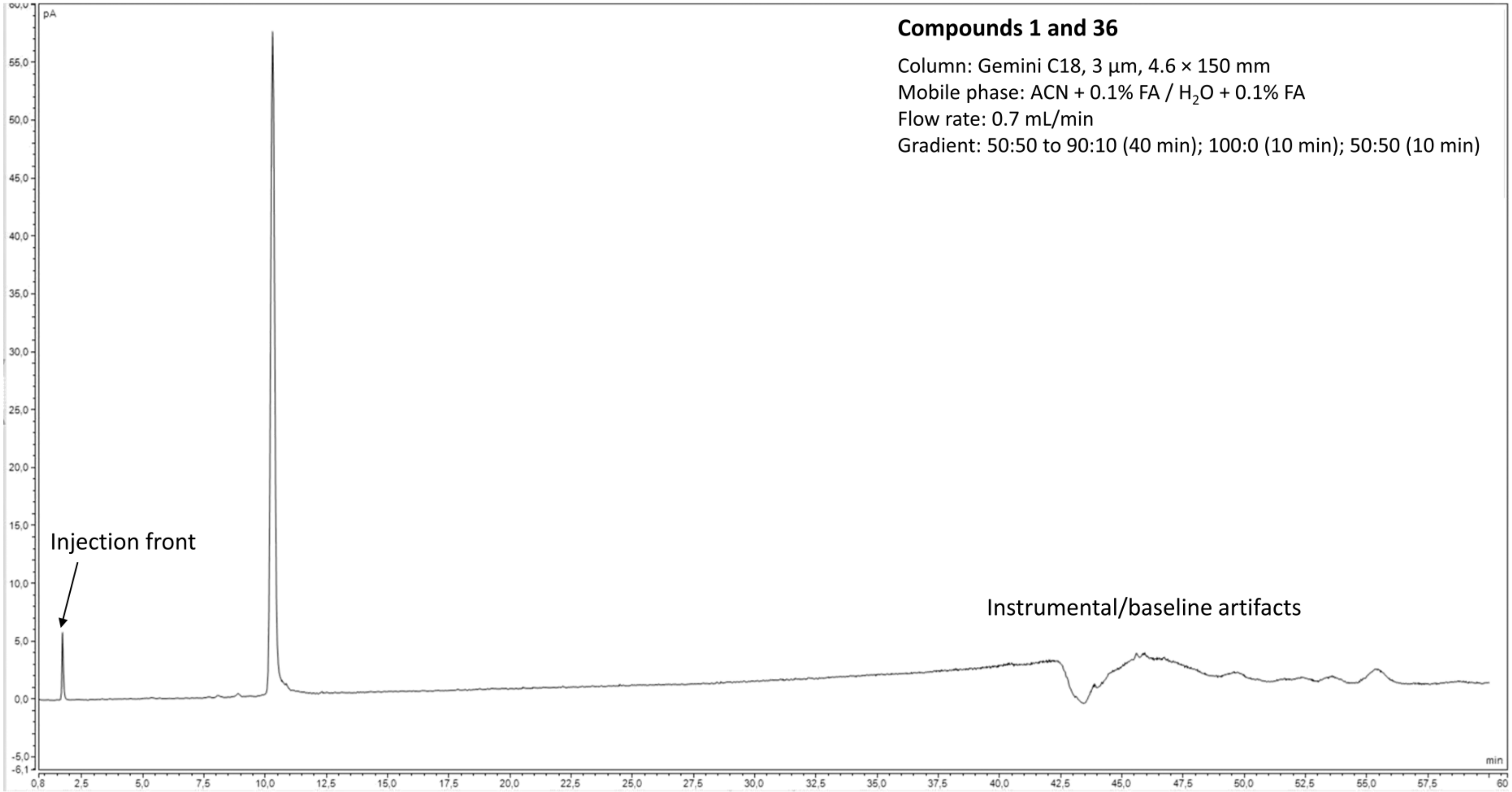

Figure S84. HPLC-CAD chromatogram of compounds **1** and **36**.

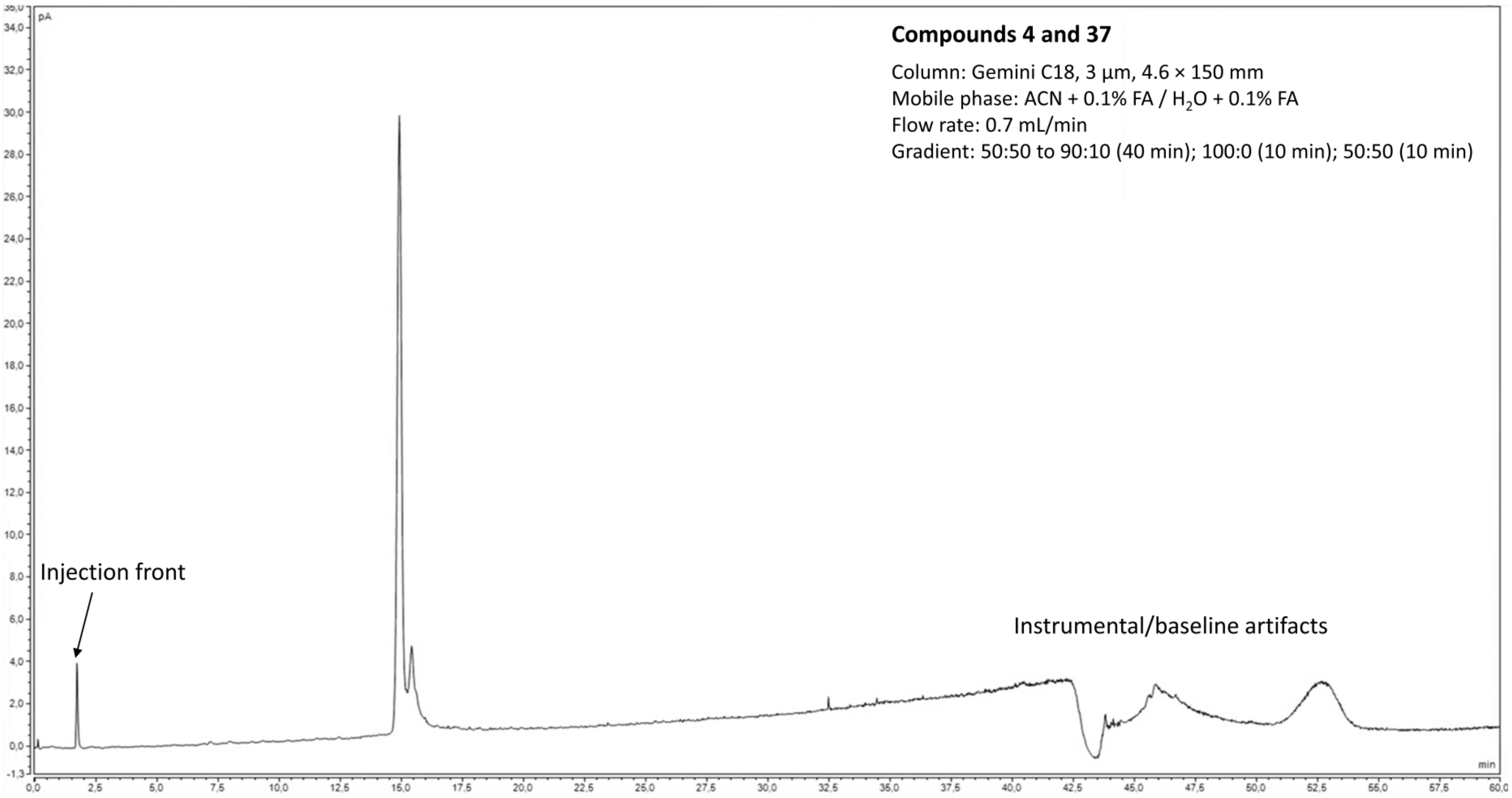

Figure S85. HPLC-CAD chromatogram of compounds **4** and **37**.

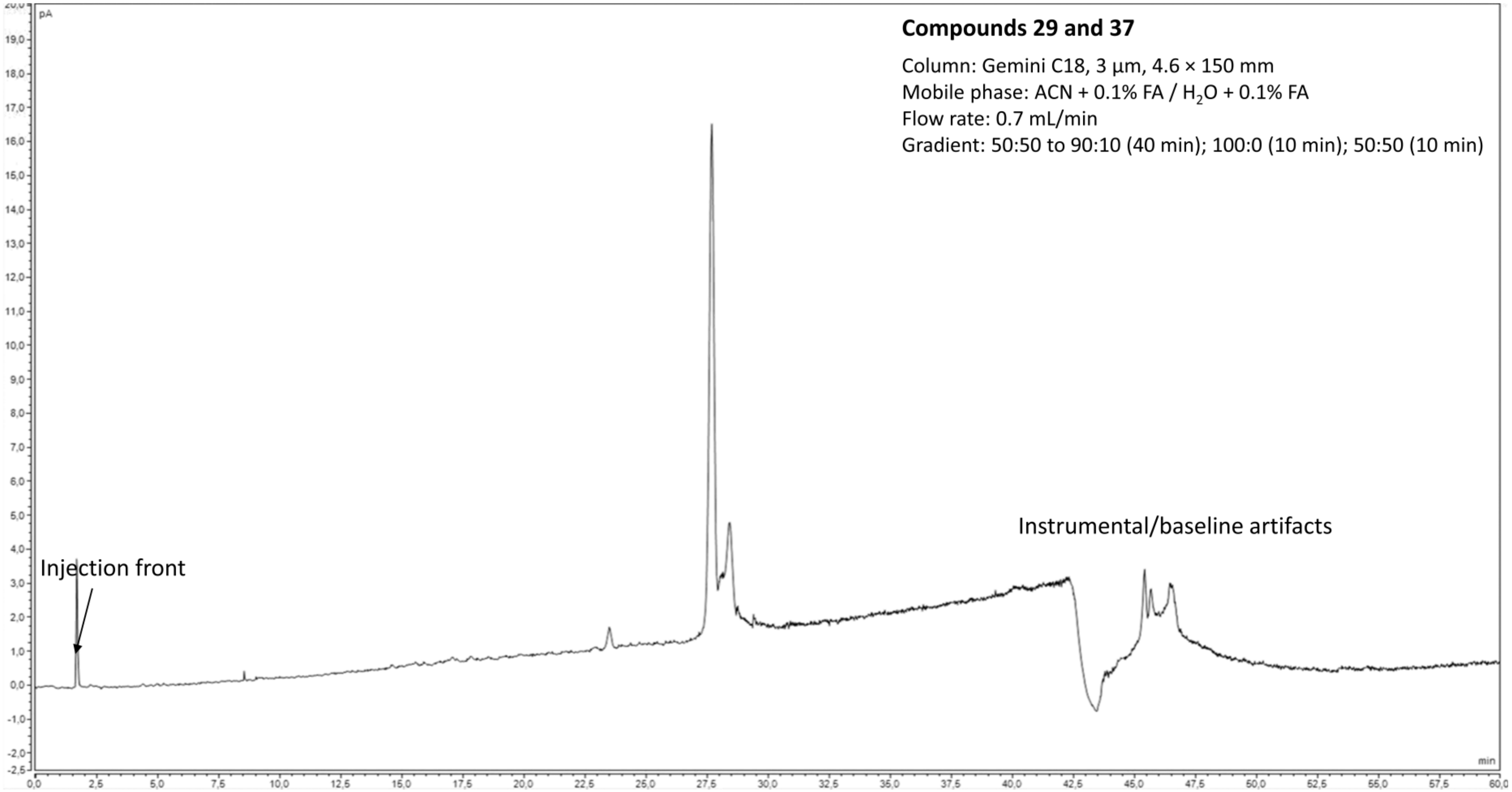

Figure S86. HPLC-CAD chromatogram of compounds **29** and **37**.

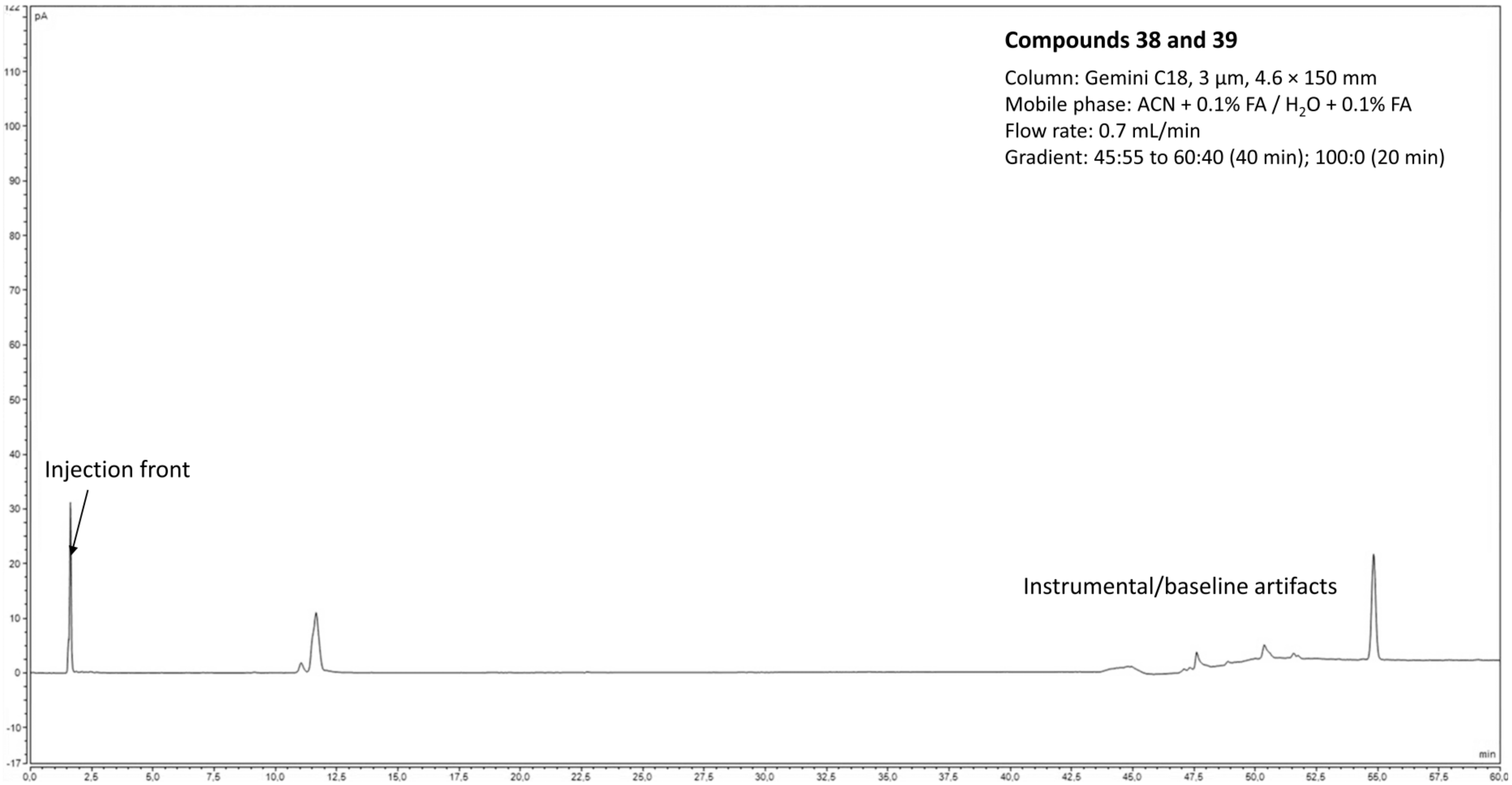

Figure S87. HPLC-CAD chromatogram of compounds **38** and **39**.
